# A mesoporous superparamagnetic iron oxide nanoparticle as a generic drug delivery system for tumor ferroptosis therapy

**DOI:** 10.1186/s12951-024-02457-w

**Published:** 2024-04-24

**Authors:** Jing Yang, Wei Xiong, Lin Huang, Zongheng Li, Qingdeng Fan, Fang Hu, Xiaopin Duan, Junbing Fan, Bo Li, Jie Feng, Yikai Xu, Xiaoyuan Chen, Zheyu Shen

**Affiliations:** 1 School of Biomedical Engineering, Southern Medical University, 1023 Shatai South Road, Guangzhou, 510515 Guangdong China; 2 Medical Imaging Center, Nanfang Hospital, Southern Medical University, 1023 Shatai South Road, Guangzhou, 510515 Guangdong China; 3 Cancer Research Institute, School of Basic Medical Sciences, Southern Medical University, 1023 Shatai South Road, Baiyun, Guangzhou, 510515 Guangdong China; 4 Departments of Diagnostic Radiology, Surgery, Chemical and Biomolecular Engineering, and Biomedical Engineering, Clinical Imaging Research Centre, Nanomedicine Translational Research Program, Yong Loo Lin School of Medicine and College of Design and Engineering, National University of Singapore, Singapore, 119228 Singapore; 5 Institute of Molecular and Cell Biology, Agency for Science, Technology, and Research (A*STAR), Singapore, 138673 Singapore

## Abstract

As a famous drug delivery system (DDS), mesoporous organosilica nanoparticles (MON) are degraded slowly in vivo and the degraded components are not useful for cell nutrition or cancer theranostics, and superparamagnetic iron oxide nanoparticles (SPION) are not mesoporous with low drug loading content (DLC). To overcome the problems of MON and SPION, we developed mesoporous SPIONs (MSPIONs) with an average diameter of 70 nm and pore size of 3.9 nm. Sorafenib (SFN) and/or brequinar (BQR) were loaded into the mesopores of MSPION, generating SFN@MSPION, BQR@MSPION and SFN/BQR@MSPION with high DLC of 11.5% (SFN), 10.1% (BQR) and 10.0% (SNF + BQR), demonstrating that our MSPION is a generic DDS. SFN/BQR@MSPION can be used for high performance ferroptosis therapy of tumors because: (1) the released Fe^2+/3+^ in tumor microenvironment (TME) can produce •OH *via* Fenton reaction; (2) the released SFN in TME can inhibit the cystine/glutamate reverse transporter, decrease the intracellular glutathione (GSH) and GSH peroxidase 4 levels, and thus enhance reactive oxygen species and lipid peroxide levels; (3) the released BQR in TME can further enhance the intracellular oxidative stress *via* dihydroorotate dehydrogenase inhibition. The ferroptosis therapeutic mechanism, efficacy and biosafety of MSPION-based DDS were verified on tumor cells and tumor-bearing mice.

**Supplementary Information:**

The online version contains supplementary material available at 10.1186/s12951-024-02457-w.

## Introduction

Mesoporous silica nanoparticles (MSN) as a drug delivery system (DDS) has attracted extensive attention due to their controllable mesoporous structure, large specific surface area, and easy surface modification [1]. Yu et al. designed openwork@dendritic mesoporous silica nanoparticles (ODMSN), which were assembled through a time-resolved lamellar growth mechanism to deliver LOX for cancer treatment [2]. Wang et al. developed a sophisticated intelligent biomimetic nanoplatform employing dendritic large-pore mesoporous silica nanoparticles (DLMSNs), capable of effectively suppressing metastatic triple-negative breast cancer through a synergistic approach involving photothermal ablation and immune remodeling [3]. Yang et al. designed a *N*-doped carbon dots/mesoporous silica nanoparticles (NCDs/MSN, ca. 40 nm) nanohybrid with peroxidase (POD)-like activity for synergistic photodynamic therapy (PDT) and enzyme-activity tumor treatment [4]. However, the non-degradability of MSN seriously limits their application as a generic DDS for the treatment of tumors.

To overcome the non-degradability problem of MSN, disulfide groups were incorporated into the framework of MSN, generating organic-inorganic hybrid mesoporous organosilica nanoparticles (MON) with the reduction-responsive degradability. Wang et al. synthesized deformable mesoporous organosilica nanoparticles (D-MON) to prolong blood circulation and enhance tumor imaging performance of the magnetic resonance imaging (MRI) contrast agent (CA) Gd-DTPA [5]. Ju et al. synthesized a sub-50 nm hollow-structured MON to prevent the potential leakage of cisplatin [6]. Nevertheless, the MON is degraded slowly in vivo due to the slow cleavage of disulfide bonds, and the degraded components are not useful for cell nutrition or cancer theranostics.

In addition, superparamagnetic iron oxide nanoparticles (SPION) were ever widely used as MRI contrast agents (CAs) and DDSs, due to their outstanding superparamagnetism, large specific surface area, easy surface modification, fast biodegradability in weakly acidic endosomes and tumor microenvironments (TME) [7]. According to the references of SPION as DDS, various chemotherapeutic drugs were directly loaded onto the surface of SPION, enabling targeted delivery to tumor sites [8]. However, the drug release is not easy to be controlled, and drug loading capacity is low. To overcome these drawbacks, the surface of SPION was coated with silica, polymers, and so on [9]. Drugs can then be loaded onto the surface coating of SPION with enhanced drug loading capacity. However, the complex structure and potential instability during in vivo delivery have limited their further applications [10]. Therefore, the development of iron oxide nanoparticles with high drug loading rates has become particularly important. Yavari et al. successfully engineered a magneto-thermal nanocarrier, wherein superparamagnetic iron oxide nanoparticles (SPIONs) were coated with a synergistic blend formulation of Pluronic F127 and F68 block copolymers on oleic acid (OA). This innovative nanocarrier system effectively loaded curcumin, a potent natural and chemical anti-cancer agent, with the aim of targeting and eradicating bone tumors [11]. Zhang et al. developed a MRI nanoprobe for differential diagnosis between benign and malignant tumors by assembling Fe^3+^-tannic acid on the surface of SPION [12]. Hou et al. developed a theranostic nanoprobe based on protein-corona-coated SPION and biomineralization in the corona to promote the ferroptosis/apoptosis of tumor cells [13]. However, these SPIONs are not mesoporous, whose drug loading contents (DLC) are much lower than the MONs.

To overcome the above-mentioned problems of MON and SPION, considering the complementary strengths of MON and SPION, a few studies have focused on the development of mesoporous SPION (MSPION). However, the reported MSPIONs have limited applications as DDSs due to their large sizes [14]. It is well-known that the particle sizes exceeding 150 nm are not conducive to long circulation in the blood and tumor accumulation because the nanoparticles with such large sizes are easy to accumulate in the reticuloendothelial system (RES), such as liver and spleen [15]. In addition, the application of the developed MSPIONs have also been limited by their complicated fabrication process [16], and unfriendly surfactants [17]. What’s more, the blood clearance profile and biodistribution properties of the reported MSPIONs have not been studied. In addition, the degraded components (Fe^2+^ and Fe^3+^) of MSPION are useful for tumor ferroptosis therapy because both Fe^2+^ and Fe^3+^ are Fenton reactants generating reactive oxygen species (ROS) [18]. However, so far, none of the MSPIONs have been investigated as DDS to promote ferroptosis of tumor cells. In this study, a novel type of MSPION with appropriate diameter (70 nm), uniform size, and good dispersibility was developed as a generic DDS for tumor ferroptosis therapy. Our MSPION was synthesized by utilizing a one-step method with bubble templates, offering a simple process with high potential for large scale production. Notably, the particle size of MSPION was adjusted by the solvent ratio of diglycol and ethylene glycol without any surfactants, which offers high biosafety. Furthermore, more biosafety and metabolism behaviors in vivo were investigated, such as blood circulation time and major organs distribution, which are previously unexplored in the existing research.

Our MSPION facilitates high drug loading content and pH-responsive drug release. This approach avoids the need for complex coating modification processes, thereby mitigating the potential instability associated with traditional SPION-based DDS and demonstrating greater potential for medical translation. The small molecular drug sorafenib (SFN) and/or brequinar (BQR) were loaded into the mesopores of MSPION, generating SFN@MSPION, BQR@MSPION and SFN/BQR@MSPION with high DLC of 11.5% (SFN), 10.1% (BQR) and 10.0% (SNF + BQR), which demonstrates that our MSPION is a generic DDS (Scheme 1a). Due to the favorable superparamagnetism of MSPION, SFN/BQR@MSPION is a good *T*_2_-weighted CA for MRI with a superhigh *r*_2_ value (246.4 mM^− 1^ s^− 1^ at 7.0 T, 186.9 mM^− 1^ s^− 1^ at 3.0 T), and very low *r*_1_ value (0.04 mM^− 1^ s^− 1^ at 7.0 T, 0.31 mM^− 1^ s^− 1^ at 3.0 T). Moreover, the SFN/BQR@MSPION can be used for high performance ferroptosis therapy of tumors because: (1) the released Fe^2+^ and Fe^3+^ in TME can produce highly toxic hydroxyl radicals (•OH) *via* Fenton reaction; (2) the released SFN in TME can inhibit the cystine/glutamate reverse transporter (System Xc^−^), decrease the intracellular cystine, glutathione (GSH) and GSH peroxidase 4 (GPX4) levels, and thus enhance the ROS and lipid peroxide (LPO) levels; (3) the released BQR in TME can further enhance the intracellular oxidative stress *via* dihydroorotate dehydrogenase (DHODH) inhibition (Scheme 1b). Furthermore, comprehensive studies on the blood clearance profile and biodistribution in organs were conducted, demonstrating the excellent biosecurity of our MSPION. In vivo antitumor experiments indicate the significant potential of our MSPION for tumor ferroptosis therapy.

**Sch. 1:**
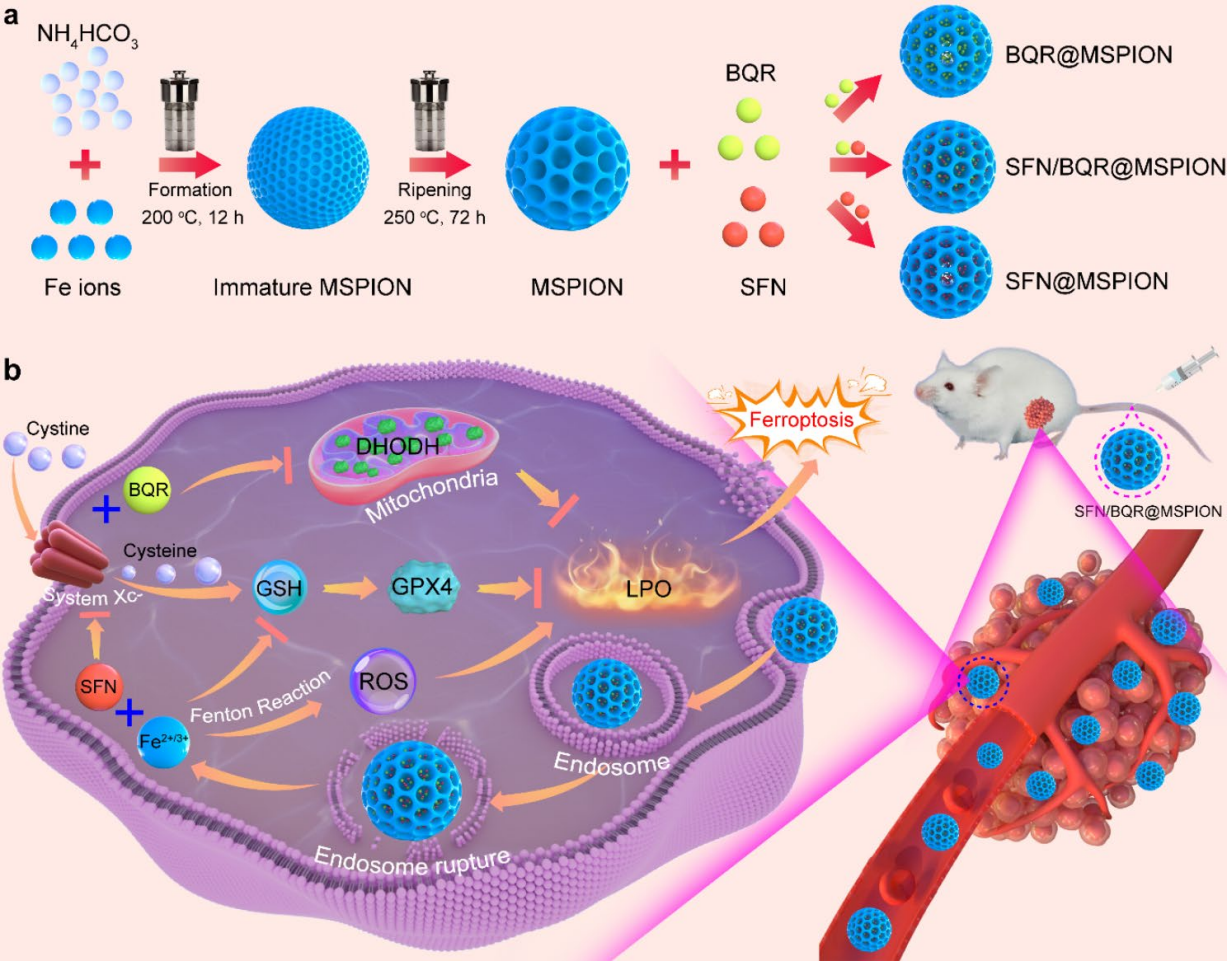
Schematic illustration for the synthesis of SFN/BQR@MSPION (**a**), and the mechanism of DHODH-mediated ferroptosis therapy (**b**)

## Results and discussion

### Preparation and characterization of MSPION

**Fig. 1:**
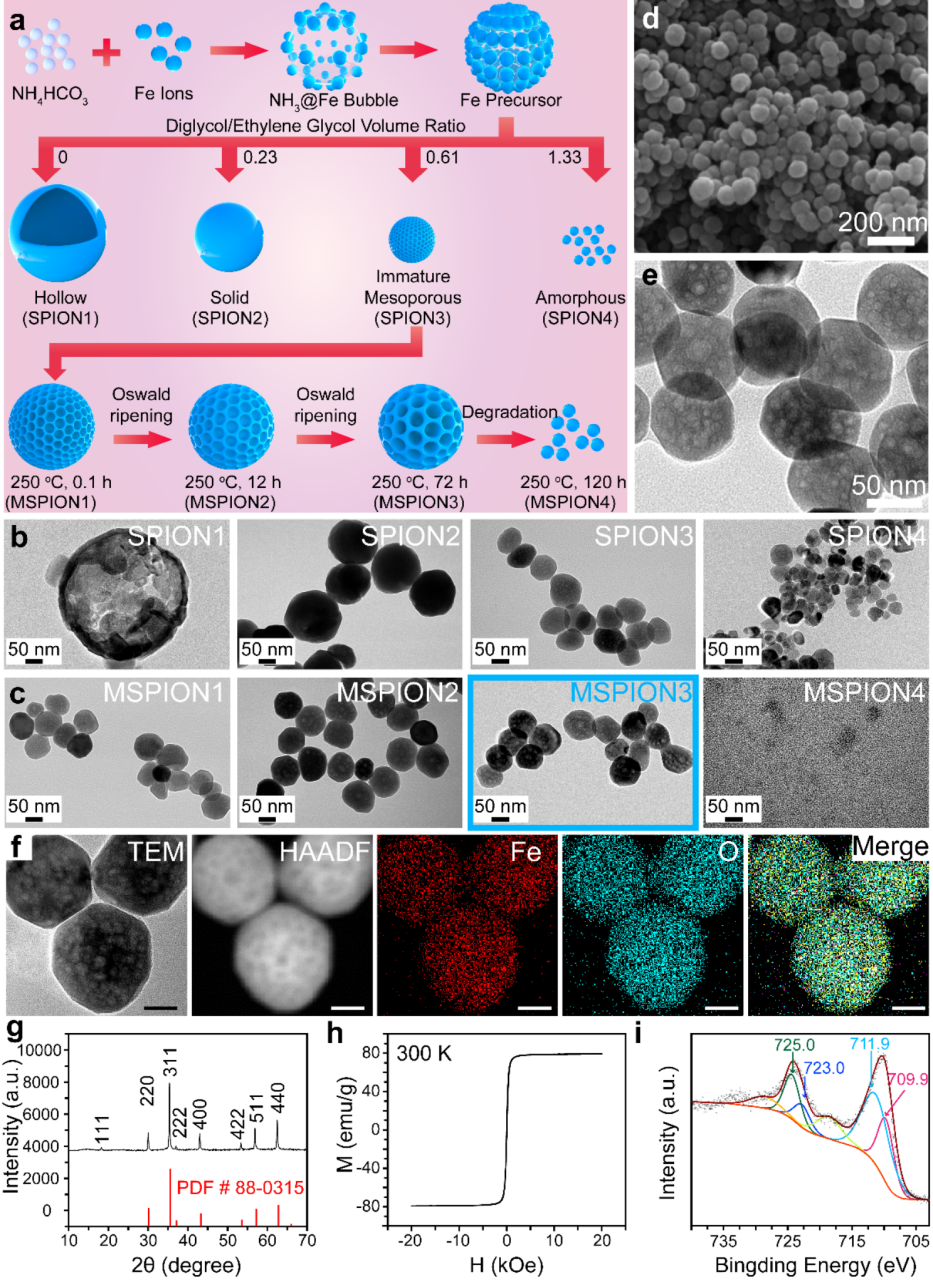
The synthesis of SPION1-4 and MSPION1-4 with physical characterization. **a**, Schematic illustration of the synthesis process of MSPION. **b, c**, TEM images of the SPION1-4 nanoparticles prepared in 30 mL mixed solvents of diglycol/ethylene glycol with the volume ratio of 0, 0.23, 0.61, or 1.33 (**b**), or the MSPION1-4 nanoparticles prepared at 250 ^o^C for 0.1 h, 12 h, 72 h, or 120 h based on SPION3 (**c**). **d, e**, SEM (**d**) and TEM (**e**) images of MSPION3 nanoparticles. **f-i**, Fe and O Elemental mapping (**f**), XRD spectrum (**g**), magnetization curve (**h**), and high-resolution XPS spectra of Fe2p (**i**) for MSPION3 without any surface coating or drug loading

The SPION and MSPION were synthesized through a bubble template method, the reaction mechanism of which is shown in the following Eqs. 1–4. Briefly, the MSPION was prepared *via* the reaction between FeCl_3_ and NH_4_HCO_3_ in the mixed solvents of diglycol and ethylene glycol with different volume ratios (Fig. 1a).

In the reaction process, the NH_4_HCO_3_ serves as a bubble producer and alkali source. After being heated to 200 °C, the NH_4_HCO_3_ begins to decompose into NH_3_·H_2_O (alkali) and CO_2_ (bubble) (Eq. 1). The NH_3_·H_2_O reacts with Fe^3+^ to form Fe(OH)_3_ colloids (Eq. 2), which further reacts with the solvent ethylene glycol (HOCH_2_CH_2_OH) to generate colloids with both Fe(OH)_3_ and Fe(OH)_2_ (Eq. 3). After heating at 200 °C for 12 h, the colloids containing both Fe(OH)_3_ and Fe(OH)_2_ were transformed into Fe_3_O_4_ nanoparticles (i.e., SPION1 in Fig. 1a, b) (Eq. 4).

1 $$N{H_4}HC{O_3} \to {\text{ }}C{O_2} + {\text{ }}N{H_3}\cdot{H_2}O$$

2 $$F{e^{3 + }} + {\text{ }}3N{H_3}\cdot{H_2}O \to {\text{ }}Fe{\left( {OH} \right)_{3{\text{ }} + }}3N{H_4}^ +$$

3 $$\eqalign{ HOC{H_2}C{H_2}OH{\text{ }} + {\text{ }}10Fe{\left( {OH} \right)_3} \to {\text{ }} & 10Fe{\left( {OH} \right)_2} \cr & + {\text{ }}2{H_2}C{O_3} + {\text{ }}6{H_2}O \cr}$$

4 $$2Fe{\left( {OH} \right)_3} + {\text{ }}Fe{\left( {OH} \right)_2} \to {\text{ }}F{e_3}{O_4} + {\text{ }}4{H_2}O$$

However, the particle size of SPION1 was larger than 200 nm (Fig. 1b), which is too large for drug delivery applications. Therefore, the diglycol was introduced into the ethylene glycol solvent to control the SPION size. SPION2-4 were synthesized with diglycol/ethylene glycol volume ratios of 0.23, 0.61, and 1.33, respectively (Fig. 1a). As shown in Fig. 1b, the size gradually decreased from SPION1 to SPION4. When the diglycol/ethylene glycol volume ratio was raised to 0.23, 0.61, and 1.33, the SPION showed a solid structure (∼182.4 nm, SPION2), mesoporous structure (∼70.8 nm, SPION3), and amorphous structure (∼37.5 nm, SPION4), respectively. That’s because the increased solvent viscosity brought by diglycol can restrict the size expansion. The synthesis conditions and results of SPION1-4 are shown in Table S1. It is noticeable that, SPION3 has a weak mesoporous structure after 12 h of reaction at 200 ^o^C. To enlarge the mesoporous structure of SPION3, the SPION3 was further heated at 250 ^o^C for 0.1–120 h (Table S2), generating MSPION1-4 (Fig. 1a). Supplementary Fig. 1 shows the photographs of the purified SPION1-4 and MSPION1-4 dispersed in ultrapure water, indicating good water dispersibility without precipitation. The surface modification of MSPION3 with polyethylene glycol (PEG) effectively enhances water dispersibility and prolongs in vivo circulation of the nanoparticles. PEG, as a surface modifier, exhibits the capability to improve the compatibility between the nanoparticles and water, thereby reducing the aggregation phenomenon and promoting water dispersibility. Moreover, the introduction of a PEG modification layer can form a stable hydration layer, which effectively prevents the direct interaction between the nanoparticles and plasma proteins, therefore reducing protein adsorption. Collectively, this strategy holds promising potential in prolonging the circulation time of the nanoparticles within the body. As shown in Fig. 1c, compared with MSPION1 and 2, MSPION3 shows apparent mesoporous structure (250 °C for 72 h at the second step of reaction) triggered by the Oswald ripening mechanism. MSPION4 shows minuteness structure (250 °C for 120 h at the second step of reaction). Thus, MSPION3 is the optimal sample among MSPION1-4 to be used as a generic DDS.

The SEM images (Fig. 1d, Supplementary Fig. 2) show that the as-prepared MSPION3 has a high quality with spherical morphologies and uniform particle sizes (∼;70.5 nm), which is good for subsequent drug delivery applications. The enlarged TEM image (Fig. 1e) shows the apparent mesoporous structure for MSPION3, which is favorable for adsorbing abundant drugs. To further verify the chemical composition of MSPION3, high-angle annular dark-field scanning transmission electron microscopy (HAADF-STEM) was used to visualize the key elements. As expected, Fe and O elements from Fe_3_O_4_ are found in Fig. 1f. The EDS spectrum in Supplementary Fig. 3 confirms the complete removal of diglycol/ethylene glycol and NH_4_HCO_3_ from MSPION3, as evidenced by the peaks corresponding to C, N, O, Fe, and Fe. From the X-ray diffraction (XRD) pattern of MSPION3 (Fig. 1g), the characteristic Fe_3_O_4_ peaks of (111), (220), (311), (222), (400), (422), (511), and (440) are found, which is in accordance with the previous reports^[18b, 19]^. As shown in Fig. 1h, the absence of a hysteresis loop in the field-dependent magnetization measurement provides clear evidence of the superparamagnetic nature of MSPION3 in its drug-free state. As shown in Supplementary Fig. 4a, MSPION3 shows a diminutive value of remanent magnetization (*M*r) of 3.0 emu/g and the coercivity (*H*cm) of 17 Oe, indicating a superparamagnetic property. In addition, the zero-field-cooled (ZFC) and field-cooled (FC) experiments measured at a magnetic field of 100 Oe is shown in Supplementary Fig. 4b. A bifurcation phenomenon between ZFC and FC starting at 400 K suggests the critical temperature (Tc) of MSPION3 powder above 400 K. As shown in the high-resolution X-ray photoelectron spectroscopy (XPS) spectra for MSPION3 (Fig. 1i), the peaks at 725.0 and 711.9 eV belong to 2P^1/2^ and 2P^3/2^ for Fe^3+^, and peaks at 723.0 and 709.9 eV are assignable to those of Fe^2+^ [19]. The proportion of trivalent iron to divalent iron in the MSPION is approximately 2:1, mirroring the ratio of trivalent to divalent iron in Fe_3_O_4_. This observation implies that the MSPION mostly consists of Fe_3_O_4_. In addition, the corresponding full-scale XPS spectra are shown in Supplementary Fig. 5, displaying the elements of Fe and O from MSPION3. Unlike the large diameter of the others mesoporous SPION [14], the as-prepared MSPION possess only ∼70 nm size, which is favor for its long circulation in vivo. Unlike the two-step acid etching processes used in obtaining mesoporous structures [16], the as-prepared MSPION was synthesized by a single step, where the reaction temperature and time are easily controlled. Unlike other systems that involve the addition of surfactants (such as CTAB) for the synthesis of SPIONs [17], the as-prepared MSPION was prepared without the use of any surfactants, which eliminates the potential toxicity and adverse reactions associated with surfactants.

### Catalytic activity of MSPION on fenton reaction

**Fig. 2:**
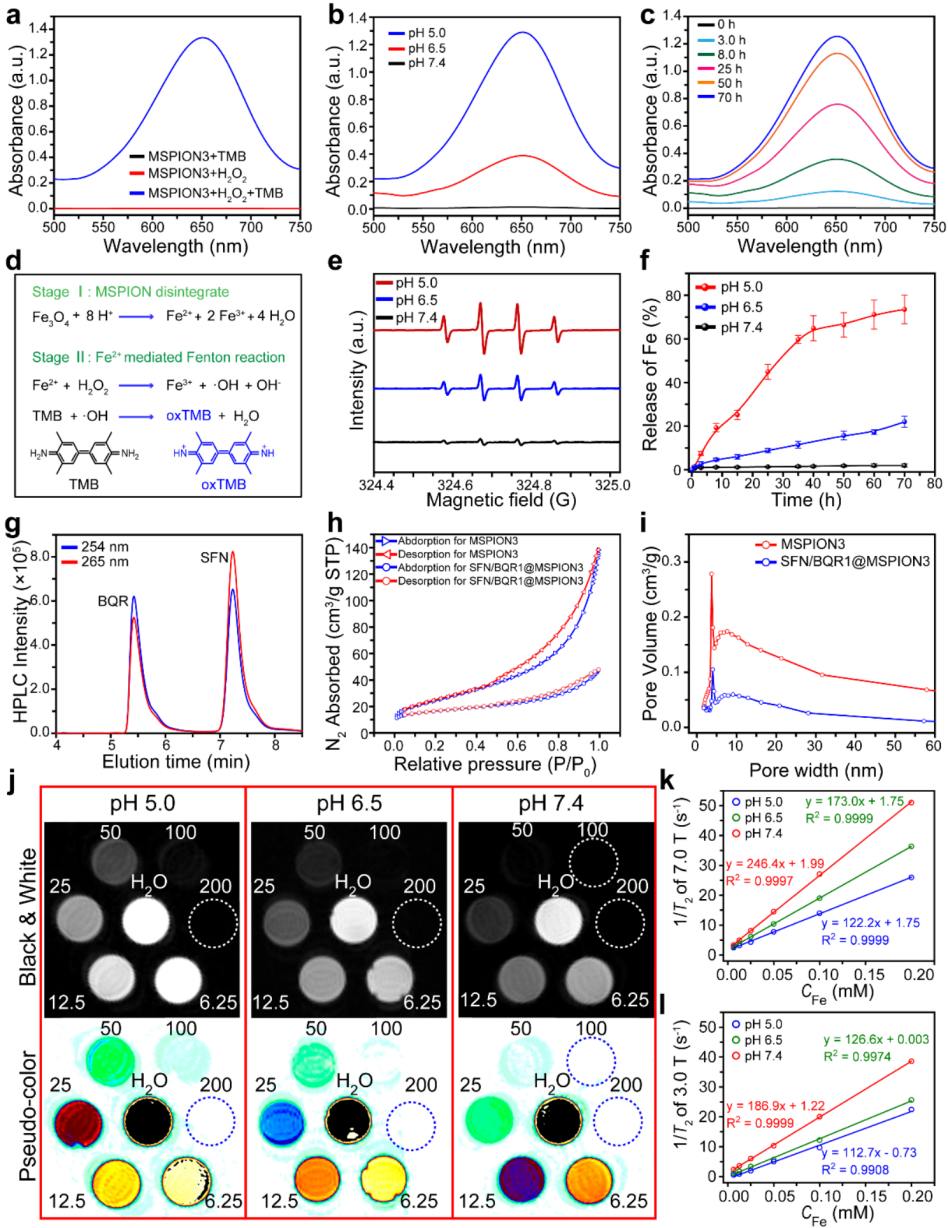
The Fenton reaction activity, drug loading, and *T*_2_-MRI performance. **a**, UV-vis spectra of the MSPION3 solutions at pH 5.0 with TMB and/or H_2_O_2_ to testify the •OH generation. **b, c**, UV-vis spectra of the MSPION3 solutions with TMB and H_2_O_2_ to measure the •OH generation at different pH values (pH 5.0, 6.5, or 7.4) for 70 h (**b**), or at pH 5.0 for different times (0, 3.0, 8.0, 25, 50, or 70 h) (**c**). **d**, Schematic illustration of the mechanism of the pH-activated Fe release from MSPION3 and the TMB colorimetric method for the measurement of •OH generation. **e**, ESR spectra measured by using 5,5-dimethyl-1-pyrroline-N-oxide (DMPO) as •OH trapping agent, indicating Fenton reaction induced •OH generation at pH 5.0, 6.5, or 7.4 for MSPION3. **f**, Fe ions release profiles (*n* = 3) in PBS solutions with pH 5.0, 6.5, or 7.4 from MSPION3. **g**, Representative HPLC chromatograms of the mixture of SFN and BQR, measured at 265 nm–254 nm. **h, i**, N_2_ adsorption-desorption isotherms (**h**), and pore-size distributions (**i**) of MSPION3 and SFN/BQR1@MSPION3 measured by the BET method. **j**, *T*_2_-weighted MR images of SFN/BQR1@MSPION3 solutions with various *C*_Fe_ (0 ∼200 µM) incubated at different pH values (5.0, 6.5, or 7.4) for 24 h observed by a 7.0 T MRI system. **k, l**, *T*_2_ relaxation rate (1/*T*_2_) plotted as a function of *C*_Fe_ for SFN/BQR1@MSPION3 in magnetic field of 7.0 T (**k**) and 3.0T (**l**). For *T*_2_ relaxation rates in magnetic field 7.0 T: TR = 6000 ms, TE = 120 ms. For *T*_2_ relaxation rates in magnetic field 3.0 T: TR = 5000 ms, TE = 80 ms

3,3’,5,5’-Tetramethylbenzidine (TMB) is a common probe of •OH, and has been widely utilized to prove the •OH generation [20], which can oxidize the TMB to be blue-green [21]. Figure 2a shows UV-vis spectra of the MSPION3 solutions at pH 5.0 with TMB and/or H_2_O_2_. No UV-vis absorption is found for MSPION3 with TMB or H_2_O_2_, but obvious UV-vis absorption with a peak at ∼652 nm appears for MSPION3 with TMB and H_2_O_2_. Once the Fe^2+/3+^ ions are released from MSPION3, the Fenton reaction is occurred immediately. Because Fe^2+^ exhibits a rapid Fenton reaction rate constant (k value) of 76 M^− 1^s^− 1^ [22]. The released Fe^3+^ can react with GSH to produce Fe^2+^ and GSSG, which can be demonstrated by the GSH consumption of MSPION3 detected using 5,5’-dithiobis (2-nitrobenzoic acid) (DTNB) assay (Supplementary Fig. 6). Therefore, the catalytic activity of MSPION on Fenton reaction should be stronger in the TME with high content of GSH.

Figure 2b shows that the amount of •OH increases with the decreasing pH value, indicating the pH dependence for the catalytic activity of MSPION3 on Fenton reaction, which is ascribed to the more rapid release of Fe^2+/3+^ from MSPION3 in more acidic environment. Figure 2c shows that the Fenton reaction continues during the period of 0–70 h, indicating the strong catalytic activity of MSPION3 for Fenton reaction in pH 5.0. This observation aligns with the pattern of iron ion release in acidic environment, supporting the notion that the •OH generation of MSPION3 is time-dependent in acidic environment. Figure 2d shows the mechanism of pH activated Fe^2+/3+^ release from MSPION3 for Fenton reaction to generate •OH, which oxidizes TMB into blue oxTMB.

Supplementary Fig. 7a shows a schematic illustration of the OPD colorimetric method for the measurement of •OH generation. Supplementary Fig. 7b shows UV-vis spectra of the MSPION3 solutions at pH 5.0 with OPD and/or H_2_O_2_. No UV-vis absorption is found for MSPION3 with OPD or H_2_O_2_, but obvious UV-vis absorption with a peak at ∼450 nm appears for MSPION3 with OPD and H_2_O_2_. Supplementary Fig. 7c shows that the amount of •OH increases with the decrease of pH value, indicating the pH dependent catalytic activity of MSPION3 on Fenton reaction.

Electron spin resonance (ESR) spectroscopy was further used to identify •OH production captured by 5,5-dimethyl-1-pyrroline N-oxide (DMPO). The ESR spectra showed the classic 1:2:2:1 signals for the •OH generation [23], with the strongest signal for the MSPION3 incubated at pH 5.0 and the weakest signal at pH 7.4 (Fig. 2e). These results demonstrate the strong catalytic activity of MSPION3 on Fenton reaction in acidic environments, which can be utilized to accelerate the Fenton reaction for high performance ferroptosis therapy.

### Drug loading performance of MSPION as a generic DDS

Figure 2f shows Fe^2+/3+^ release profiles in PBS solutions with pH 5.0, 6.5, or 7.4 from MSPION3. It can be seen that the Fe^2+/3+^ release is negligible at pH 7.4, suggesting that MSPION3 is stable in neutral environment. However, under acidic conditions (pH = 6.5 or 5.0), Fe^2+/3+^ can be released from MSPION3 due to the reaction between H^+^ and the main component Fe_3_O_4_ of MSPION3. Therefore, the MSPION3 is promising to be used as a generic DDS that can specifically release loaded drugs in acidic TME.

The high performance liquid chromatography (HPLC) chromatograms of the mixture of SFN and BQR measured at 265 nm–254 nm (Fig. 2g) indicate that the HPLC can be used to measure the concentrations of SFN at 265 nm, and BQR at 254 nm. Supplementary Fig. 8a, b show the chromatograms of SFN or BQR solutions with various concentrations, and Supplementary Fig. 8c, d show the corresponding standard curves of SFN or BQR constructed from the maximum HPLC intensity as a function of the SFN or BQR concentrations. The high SFN and/or BQR loading content (LC) and loading efficiency (LE) measured according to the standard curves for SFN1-3@MSPION3, BQR1-3@MSPION3, and SFN/BQR1-2@MSPION3 (Table S3) demonstrate that our MSPION3 can be used as a generic DDS.

According to previous reports, the redox system is responsible for ferroptosis [24]. We thus hypothesized that a combination of SFN and BQR may have synergistic effect on the enhancement of ferroptosis [25]. The cytotoxic effect on 4T1 cells was significantly enhanced by the combination of SFN and BQR (Supplementary Fig. 9a, b). The IC_50_ value of SFN plus BQR with molar ratio of 1.0 is 2.5 µM, which is significantly lower than that with molar ratio of 0.2, 0.5, 2.0 and 5.0, and that of the individual drug (101.4 µM for SFN, or 67.1 µM for BQR) (Supplementary Fig. 9c). These results indicate that SFN plus BQR at 1:1 molar ratio can provide synergistic effects at lower doses with a CI value of 0.06.

The SFN and BQR were simultaneously loaded into MSPION3 with different feeding drug amounts to obtain SFN/BQR1@MSPION3 and SFN/BQR2@MSPION3, with the loaded SFN to BQR ratios of 0.99 and 0.98, respectively. SFN/BQR1@MSPION3 is chosen as the optimal sample for the subsequent anti-cancer therapy because the loaded SFN to BQR molar ratio is closer to 1.0. The mechanism of loading SFN or BQR onto MSPION is based on the encapsulation effect of MSPION. Mesoporous nanoparticles possess highly ordered pore structures with numerous channels and voids, providing a large surface area and accommodating space. These channels and voids can adsorb and encapsulate hydrophobic drugs through capillary effects, effectively embedding the drugs within the carrier.

Figure 2h shows the N_2_ adsorption-desorption isotherms used to examine the mesoporous structure of MSPION3 and SFN/BQR1@MSPION3. BET surface areas of MSPION3 and SFN/BQR1@MSPION3 are measured to be 88.5 and 38.5 m^2^/g, respectively. Compared to MSPION3, the specific surface area of SFN/BQR1@MSPION3 is decreased, which further indicates that SFN and BQR are loaded into the mesopores of MSPION3. Figure 2i shows the pore size distribution curves of MSPION3 and SFN/BQR1@MSPION3, which indicates the presence of mesopores on the MSPION3 surface. The average pore size of MSPION3 is ∼3.9 nm, which is favorable for the efficient loading and sustained release of SFN and BQR.

Supplementary Fig. 10 shows negative zeta potentials (∼ -30 mV) of SPION1-4, MSPION1-4, SFN1@MSPION3, BQR1@MSPION3, and SFN/BQR1@MSPION3 in ultrapure water, because MSPION3 nanoparticles were synthesized using the polyol method. During the reaction process, ethylene glycol and diethylene glycol were used as capping agents to control the particle size and surface functionalities of the nanoparticles. Due to the presence of abundant hydroxyl groups in ethylene glycol, the as-prepared MSPION3 nanoparticles exhibit a high density of hydrophilic hydroxyl groups on their surface. Additionally, the surface of the synthesized MSPION3 nanoparticles carries a significant negative charge, approximately − 30 mV. This high negative charge contributes to strong electrostatic repulsion, preventing the aggregation of SPIONs in aqueous media and enhancing their dispersibility [26]. Dynamic light scattering (DLS) measurements indicate that the hydrodynamic diameters of MSPION3, SFN1@MSPION3, BQR1@MSPION3 and SFN/BQR1@MSPION3 are 146.7 nm, 156.5, 171.3 nm, and 217.2 nm, respectively (Supplementary Fig. 11). Considering that the environment of pure water differs from physiological conditions, the DLS size of MSPION3 under simulated physiological condition (DMEM + 10% FBS) is shown in Supplementary Fig. 12, which is approximately 232.5 nm. This increase in observed size may be attributed to the partial aggregation of nanoparticles in the presence of proteins and other molecules in the physiological environment. Supplementary Fig. 13 shows that SFN/BQR1@MSPION3 exhibits slow SFN and BQR release at pH = 7.4, reflecting negligible drug leakage in normal tissue environments. In addition, after 35 h of incubation at 37 ^o^C, ∼ 75% and ∼36% of SFN and BQR are released from SFN/BQR1@MSPION3 at pH 5.0 and 6.5, which indicates that acidic endosomes and tumor microenvironments (TME) can promote the drug release for tumor therapy. After incubation at pH 7.4 for 24 h, the release of SFN and BQR from SFN/BQR1@MSPION3 is less than 13%, which is rather low and does not induce significant toxicity to normal tissues.

### *T*_2_-Weighted MRI capability of SFN/BQR@MSPION

Figure 2j and Supplementary Fig. 14a respectively show *T*_2_-weighted MR images of SFN/BQR1@MSPION3 solutions with various *C*_Fe_ incubated at pH 5.0, 6.5 or 7.4 for 24 h observed by a 7.0 or 3.0 T MRI scanner system. The gradual concentration gradient of the MR images demonstrates the strong *T*_2_-weighted MRI performance of our SFN/BQR1@MSPION3. The pH-dependent *T*_2_-weighted MRI capability of SFN/BQR1@MSPION3 can be attributed to the pH-induced degradation of the MSPION3.

Figure 2k and Supplementary Fig. 14b show that SFN/BQR1@MSPION3 has a high *r*_2_ value (122.2 mM^− 1^ s^− 1^ at pH 5.0, 173.0 mM^− 1^ s^− 1^ at pH 6.5, and 246.4 mM^− 1^ s^− 1^ at pH 7.4) and very low *r*_1_ value (less than 0.11 mM^− 1^ s^− 1^) measured by a 7.0 T MRI scanner system. Figure 2l and Supplementary Fig. 14c also show that SFN/BQR1@MSPION3 exhibits a high *r*_2_ value (112.7 mM^− 1^ s^− 1^ at pH 5.0, 126.6 mM^− 1^ s^− 1^ at pH 6.5, and 186.9 mM^− 1^ s^− 1^ at pH 7.4) and very low *r*_1_ value (less than 0.50 mM^− 1^ s^− 1^) observed by a 3.0 T MRI scanner system. The *r*_2_ of SFN/BQR1@MSPION3 in neutral environment is higher than that of commercial MRI CAs, e.g., Resovist (143 mM^− 1^ s^− 1^) and Feridex (93 mM^− 1^ s^− 1^) [27]. This favorable *r*_2_ and high *r*_2/_*r*_1_ can be ascribed to the powerful superparamagnetic property [28]. All these results confirm that the SFN/BQR1@MSPION3 can be a promising candidate as a *T*_2_-weighted MRI CA.

### Cellular uptake and lysosome escape

**Fig. 3:**
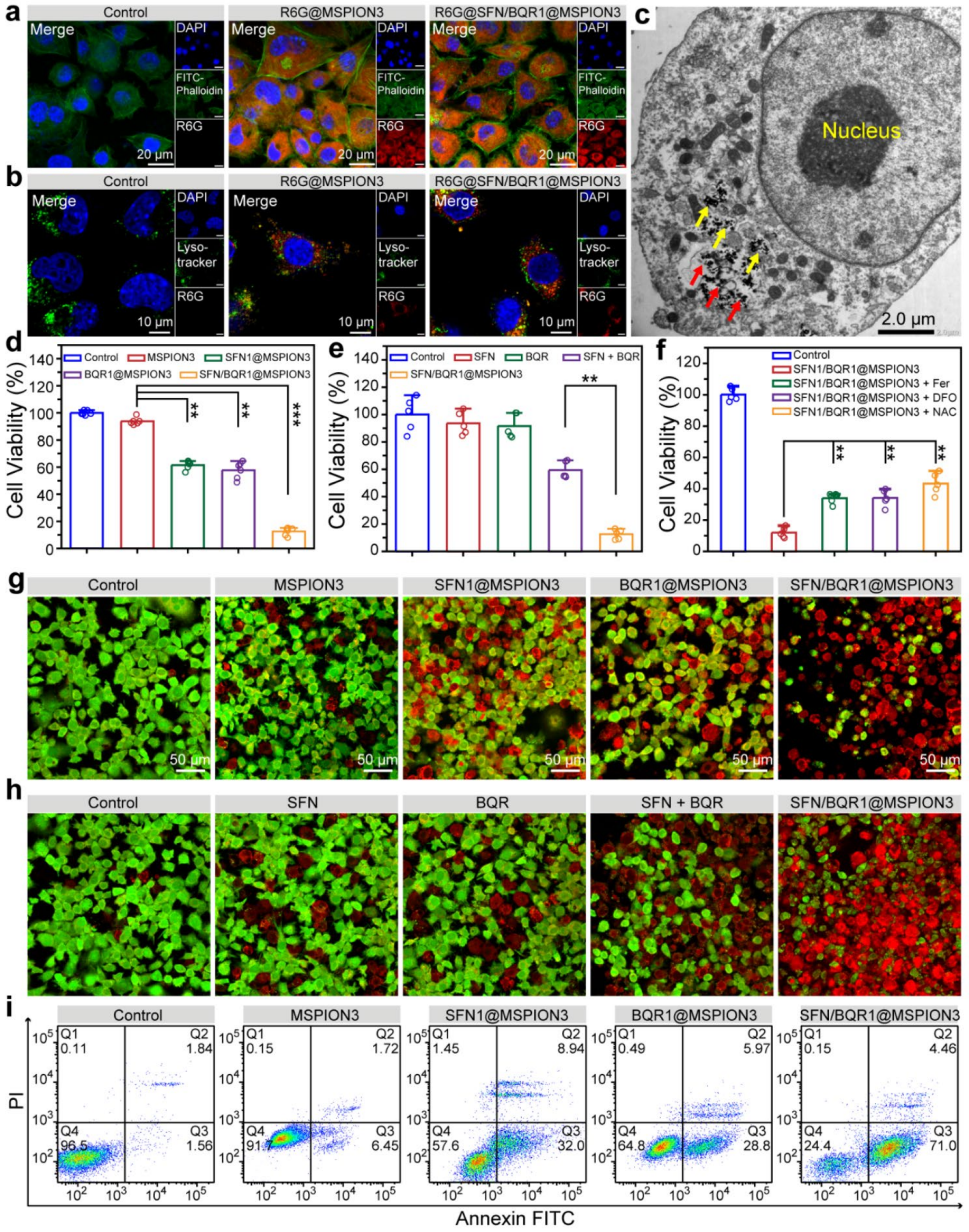
The cellular uptake, endosomal escape and cytotoxicity of SFN1/BQR1@MSPION3. **a**, CLSM images of 4T1 cells showing intracellular uptake of R6G@MSPION3 and R6G@SFN/BQR1@MSPION3 at 4.0 h time point. Green fluorescence: FITC-Phalloidin for cytoskeleton. Red fluorescence: R6G for nanoparticles. Blue fluorescence: DAPI for nuclei. **b**, CLSM images of 4T1 cells treated without or with R6G@MSPION3 or R6G@SFN/BQR1@MSPION3 for 4.0 h, showing lysosomal escape of the nanoparticles. Green fluorescence: Lysotracker Green for lysosomes. Red fluorescence: R6G for nanoparticles. Blue fluorescence: DAPI for nuclei. **c**, TEM images of 4T1 cells treated with SFN/BQR1@MSPION3 for 4.0 h, indicating escaped MSPION3 from lysosomes (red arrows) and remaining (yellow arrows) MSPION3 in lysosomes. **d**, 4T1 cell viabilities after incubation with PBS (control), MSPION3, SFN1@MSPION3, BQR1@MSPION3, or SFN/BQR1@MSPION3 (*C*_Fe_ = 10 µg/mL) for 24 h. **e**, 4T1 cell viabilities treated with PBS (control), SFN, BQR, SFN + BQR, or SFN/BQR1@MSPION3 (*C*_Fe_ = 10 µg/mL) for 24 h. **f**, Cell viabilities of 4T1 cells treated with SFN1/BQR1@MSPION3 (*C*_Fe_ = 10 µg/mL) without or with addition of ferroptosis inhibitors including Fer-1 (2.0 µM), DFO (100 µM), or NAC (2.0 mM). **g**, CLSM images of 4T1 cells with calcein-AM and PI staining incubated with PBS (control), MSPION3, SFN1@MSPION3, BQR1@MSPION3, or SFN/BQR1@MSPION3 (*C*_Fe_ = 10 µg/mL) for 24 h. **h**, CLSM images of 4T1 cells with calcein-AM and PI staining incubated with PBS (control), SFN, BQR, SFN + BQR, or SFN/BQR1@MSPION3 (*C*_Fe_ = 10 µg/mL) for 24 h. **i**, Apoptosis of 4T1 cells with Annexin V-FITC/PI double staining after various treatments for 24 h measured by flow cytometry. **P < 0.01

It is essential for the anticancer agent to be properly uptaken by tumor cells to exert its anticancer effects. Therefore, confocal laser scanning microscope (CLSM) and flow cytometry were used to study the cellular uptake behaviors of SFN/BQR1@MSPION3 after incubation with 4T1 cells. Figure 3a and Supplementary Fig. 15 shows CLSM images of 4T1 cells, whose cytoskeleton and nuclei are respectively stained with FITC-Phalloidin (green fluorescence) and DAPI (blue fluorescence). As a water-soluble cationic fluorescent dye, free R6G preferentially interacts with negatively charged biomolecules such as DNA upon entering cells, resulting in red signals in the cell nuclei. However, when R6G-labeled MSPIONs with a particle size of approximately 70 nm are taken up by cells, they cannot penetrate the nuclear pore and therefore exhibit abundant red signals in the cytoplasm. Hence, following cellular uptake of R6G-labeled MSPIONs, the cytoplasm emits red fluorescent signals, while the cell nuclei remain non-fluorescent. As shown in Supplementary Fig. 16a, b, 4T1 cells incubated with R6G@SFN/BQR1@MSPION3 exhibit increased red fluorescence intensity with increasing incubation time from 2.0 to 8.0 h. The CLSM and flow cytometry results reveal the robust cellular uptake of MSPION3 and SFN/BQR1@MSPION3.

Internalized nanoparticles can be quickly digested after being caught in the endo/lysosome. Therefore, escape from the endo/lysosome is crucial for drug-loaded nanoparticles [29]. To investigate the lysosome escape ability, the cell nuclei, lysosome and nanoparticles (i.e., MSPION3 and SFN/BQR1@MSPION3) were respectively stained with DAPI, Lysotracker Green and R6G (Fig. 3b). After incubation for 4, abundant R6G-SFN/BQR1@MSPION3 nanoparticles are located within the cytoplasm and lysosome, showing partial lysosomal escape (Fig. 3c). This partial lysosomal escape can be attributed to the release of Fe^2+^ from MSPION3 under acidic condition, which leads to the conversion of intracellular H_2_O_2_ into •OH through Fe^2+^ catalysis, resulting in membrane damage [30]. The results of cellular uptake and lysosome escape demonstrate that our MSPION as a generic DDS can deliver SFN and BQR into tumor cells for anti-cancer therapy.

### Ferroptosis therapeutic efficacy and mechanism evaluated on tumor cells

The relative cell viability of 4T1 tumor cells was determined using an MTT kit after incubation with various nanoparticles of MSPION3, SFN1@MSPION3, BQR1@MSPION3, and SFN/BQR1@MSPION3. It can be found that MSPION3 exhibits relatively low cytotoxicity (i.e., low ferroptosis) to 4T1 cells at 50 µg/mL of Fe concentration, which can be attributed to the well-established antioxidant mechanisms in tumor cells (Supplementary Fig. 17), indicating that MSPION can serve as a biocompatible drug carrier. Intriguingly, the viability of 4T1 tumor cells decreases mildly in response to SFN1@MSPION3 or BQR1@MSPION3 treatment but drops much more significantly in response to SFN/BQR1@MSPION3 treatment after incubation for 24 h (**P < 0.01) (Fig. 3d). Figure 3e shows that 4T1 cells possessed only 12.5% of cell viability after treatment with SFN/BQR1@MSPION3. However, the groups of SFN, BQR and SFN + BQR at equipotent drug concentrations showed much higher cell viabilities, which were 93.5%, 91.6% and 59.4%, respectively. Figure 3f shows the cell viabilities of 4T1 cells treated with SFN1/BQR1@MSPION3 (*C*_Fe_ = 10 µg/mL) without or with addition of ferroptosis inhibitors including Fer-1 (2.0 µM), DFO (100 µM), or NAC (2.0 mM). The cell viabilities of the groups with ferroptosis inhibitors are much higher than those without ferroptosis inhibitors, which indicates that the tumor cells death is ferroptosis without doubt. Additionally, calcein-AM and PI were used to label live and dead cells for CLSM imaging according to the previously reported method [31]. Figure 3g shows CLSM images of 4T1 cells with calcein-AM and PI staining. The strong green fluorescence signals in the control and MSPION3 groups indicate that the 4T1 cells are undamaged. The red signals start to occur in the SFN1@MSPION3 and BQR1@MSPION3 groups with decreased green signals, indicating that the cell death happens due to ferroptosis. Intense red fluorescence is observed in the SFN/BQR1@MSPION3 group with little green fluorescence, indicating a more significant cytotoxicity. Figure 3h showed CLSM images of 4T1 cells with calcein-AM and PI staining after the same treatments, which double confirmed the potent anticancer effect of SFN/BQR1@MSPION3 and low efficacies of SFN, BQR and SFN + BQR. Although SFN and BQR alone can reduce the antioxidant level of tumor cells, making them more sensitive to ROS and result in ferroptosis, the ferroptosis efficacy of SFN or/and BQR is limited due to the lack of ROS generation. Therefore, the enhanced anticancer efficacy of SFN/BQR1@MSPION3 manifested the synergistic ferroptosis effect of MSPION3, SFN and BQR. Figure 3i and Supplementary Fig. 18 shows the apoptosis of 4T1 cells with Annexin V-FITC/PI double staining after various treatments for 24 h measured by flow cytometry, and Supplementary Fig. 19 shows the corresponding early and total apoptosis statistics of 4T1 cells. The group treated with SFN/BQR1@MSPION3 has the highest percentage of apoptotic cancer cells compared to that treated with MSPION3, SFN1@MSPION3 or BQR1@MSPION3 (**P < 0.01), which is consistent with the MTT assay results and reinforces the synergistic ferroptosis effect of the MSPION3, SFN and BQR.

Supplementary Fig. 20 shows CLSM images of 4T1cells treated with PBS (control), MSPION3, SFN1@MSPION3, BQR1@MSPION3, or SFN/BQR1@MSPION3 (*C*_Fe_ = 10 µg/mL) for 24 h, in which the damaged DNA is stained with γ-H2AX showing green fluorescence. Obviously, the 4T1 cells treated with SFN/BQR1@MSPION3 show most severe DNA damage, compared with that treated with control, MSPION, SFN1@MSPION3 or BQR1@MSPION3. The severe DNA damage results from the strong ferroptosis generated by SFN/BQR1@MSPION3.

Supplementary Fig. 21 shows CLSM images of 4T1 cells treated without or with MSPION3 or SFN/BQR1@MSPION3 for 6.0 h, in which the green fluorescence from RhoNox-1 indicates intracellular Fe^2+^, the red fluorescence from LysoTracker means lysosomes, and the blue fluorescence from DAPI represents nuclei. Abundant green intracellular Fe^2+^ can be observed outside the red lysosomes and blue nuclei in the groups treated with MSPION3 or SFN/BQR1@MSPION3, indicating that the degraded Fe^2+^ ions can escape from the lysosomes and enter the cytoplasm. Therefore, both MSPION3 and SFN/BQR1@MSPION3 can enhance the intracellular Fe^2+^ concentration, which is easy to accelerate the Fenton reaction in 4T1 cells, generating highly toxic •OH.

**Fig. 4:**
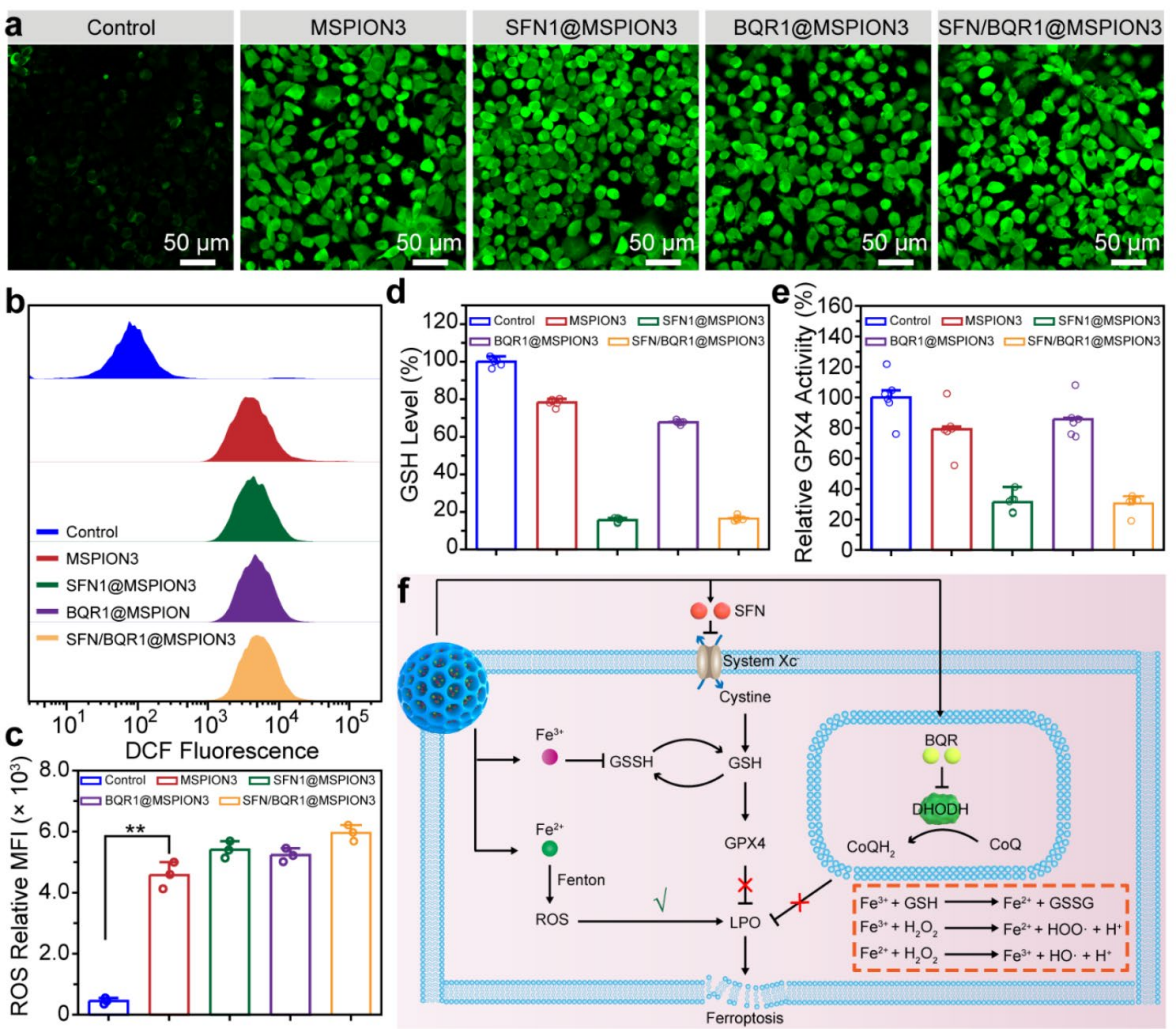
Ferroptosis therapeutic mechanism evaluated on tumor cells. **a**, CLSM images of 4T1 cells treated with PBS (control), MSPION3, SFN1@MSPION3, BQR1@MSPION3, or SFN/BQR1@MSPION3, and stained with DCFH-DA, showing intracellular ROS generation. **b, c**, ROS fluorescence distributions (**b**) and quantified ROS fluorescence intensities (**c**) of the 4T1 cells after various treatments evaluated by flow cytometry. **d, e**, The relative intracellular GSH level (**d**), and GPX4 level (**e**) in 4T1 cells after treatment with PBS (control), MSPION3, SFN1@MSPION3, BQR1@MSPION3, or SFN/BQR1@MSPION3. **f**, Schematic illustration of the underlying mechanism of the intracellular redox balance disrupted by SFN/BQR1@MSPION3. The SFN/BQR1@MSPION3 facilitates the Fenton reaction, attenuates GPX4 activity, and blocks the DHODH system, synergistically potentiating LPO accumulation and cancer cell ferroptosis

The intracellular generation of reactive oxygen species (ROS) in 4T1 tumor cells was subsequently verified using 2,7-dichlorofluorescein diacetate (DCFH-DA) as a fluorescent probe. This particular probe undergoes oxidation by ROS, resulting in the production of highly emissive DCF, thus confirming the presence of intracellular ROS [32]. Figure 4a shows CLSM images of 4T1 cells treated with PBS (control), MSPION3, SFN1@MSPION3, BQR1@MSPION3, or SFN/BQR1@MSPION3, and stained with DCFH-DA. Compared with the control group, strong green fluorescence emerges in the MSPION3, SFN1@MSPION3, and BQR1@MSPION3 treatment groups. That’s because the strong Fenton reaction is triggered by endogenous H_2_O_2_ and released Fe^2+/3+^ ions, suggesting that MSPION3 can serve as a ROS engine for ferroptosis-induced tumor therapy. Figure 4b, c show the ROS fluorescence distributions and quantified ROS fluorescence intensities of the 4T1 cells after various treatments evaluated by flow cytometry, which also verify the enhanced ROS generation for the MSPION3 group.

In order to comprehend the intracellular level of GSH affected by SFN/BQR1@MSPION3, the GSH level in 4T1 cells was investigated *via* DTNB assay [33]. Figure 4d shows the relative intracellular GSH level in 4T1 cells after treatment with PBS (control), MSPION3, SFN1@MSPION3, BQR1@MSPION3, or SFN/BQR1@MSPION3, and Supplementary Fig. 22 shows the corresponding image of 4T1 cell solutions after GSH assay. Supplementary Fig. 23 presents CLSM images of 4T1 cells following treatment and subsequent staining with Thiol Tracker Violet, revealing the presence of GSH through green fluorescence. From these data, it can be seen that the intracellular GSH is decreased by MSPION3 because the ferric ions (Fe^3+^) can deplete GSH through the catalyzed reductive reaction to produce ferrous ions (Fe^2+^) and oxidized glutathione (GSSG) (i.e., 2 Fe^3+^ + 2 GSH → 2 Fe^2+^ + GSSG + 2 H^+^) [34], especially at high levels of GSH in tumor cells. In addition, SFN1@MSPION3 and SFN/BQR1@MSPION3 groups have much lower GSH levels (∼16%) than that of MSPION3 (∼78%) and BQR1@MSPION3 (∼68%). The decrease of GPX4 expression is also one of the indicators for ferroptosis [35]. Figure 4e shows GPX4 level in 4T1 cells after treatment with PBS (control), MSPION3, SFN1@MSPION3, BQR1@MSPION3, or SFN/BQR1@MSPION3, measured by a ELISA kit. The SFN1@MSPION3 and SFN/BQR1@MSPION3 groups have much lower GPX4 levels (i.e., 31.4 and 30.5%) than that of MSPION3 (∼79%) and BQR1@MSPION3 (∼86%) after 24 h of incubation. The measurement results of intracellular GSH and GPX4 levels reinforce that SFN1@MSPION3 and SFN/BQR1@MSPION3 possess SFN-dependent abilities of GSH depletion and GPX4 inhibition for cancer therapy because SFN can inhibit the system X_C_^−^, decrease the intracellular cysteine and GSH levels, and downregulate the GPX4 expression [36].

Figure 4f shows a schematic illustration of the synergistic ferroptosis mechanism of MSPION3, SFN, and BQR released from SFN/BQR1@MSPION3: (1) The Fe^2+^ released from MSPION generates •OH through the Fenton reaction and further increases intracellular LPO, evoking the initial ferroptosis. (2) SFN downregulates GPX4 expression by inhibition of the system X_C_^−^, resulting in accumulation of intracellular LPO. (3) BQR restrains the DHODH-mediated tumor antioxidant system in mitochondria, leading to enhancement of LPO accumulation.

The decrease of mitochondrial membrane potential is also one of the indicators for ferroptosis [37]. Supplementary Fig. 24 shows the CLSM images of the 4T1 cells after treatment with PBS (control), MSPION3, SFN1@MSPION3, BQR1@MSPION3, or SFN/BQR1@MSPION3 for 24 h, indicating the changes of the mitochondrial membrane potential of 4T1 cells. The mitochondria treated with PBS or MSPION3 exhibits intense red fluorescence (JC-1 aggregates), demonstrating that the mitochondrial membrane potential is not altered. SFN1@MSPION3 and BQR1@MSPION3 groups exhibit significant green fluorescence (JC-1 monomer) and minimal red fluorescence, suggesting that the mitochondrial membrane has been damaged and exhibits a moderate potential. 4T1 cells treated with SFN/BQR1@MSPION3 exhibit the strongest green fluorescence and the weakest red fluorescence, indicating a highly synergistic action of Fe^2+/3+^ ions, SFN and BQR to damage the mitochondria thus decreasing the mitochondrial membrane potential.

**Fig. 5:**
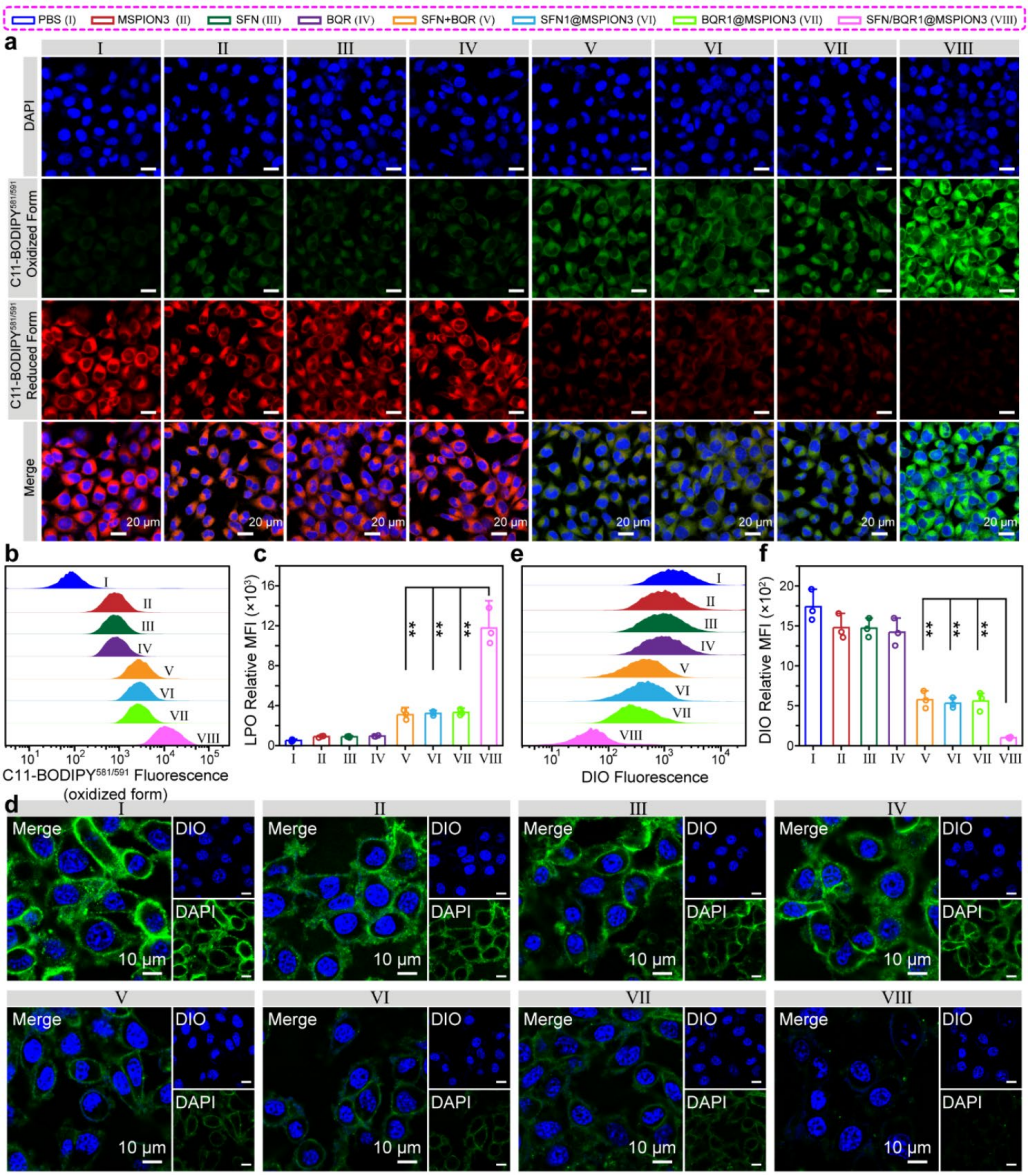
Ferroptosis parameters evaluated on tumor cells. a-c, CLSM images (**a**), C11-BODIPY^581/591^ (oxidized form) fluorescence distributions (**b**) and quantitative analysis of the C11-BODIPY^581/591^ (oxidized form) fluorescence intensities measured by flow cytometry (**c**) of the 4T1 cells after treatment with PBS (I), MSPION3 (II), SFN (III), BQR (IV), SFN + BQR (V), SFN1@MSPION3 (VI), BQR1@MSPION3 (VII), or SFN/BQR1@MSPION3 (VIII) for 24 h, and staining with C11-BODIPY^581/591^. The observed increase in green fluorescence (oxidized form) and decrease in red fluorescence (reduced form) indicate the generation of lipid peroxidation (LPO). **d-f**, CLSM images (**d**), green fluorescence distributions (**e**), and quantitative analysis of the DIO fluorescence intensities measured by flow cytometry (**f**) of 4T1 cells after different treatments for 24 h, showing the structural integrity of the cell membrane

To further explore the detailed synergetic mechanism of MSPION3, SFN and BQR to induce ferroptosis, C11-BODIPY^581/591^ was used to detect the intracellular levels of LPO, a crucial marker of ferroptosis [38]. Figure 5a-c show CLSM images, C11-BODIPY^581/591^ (oxidized form) fluorescence distributions, and quantitative analysis of the C11-BODIPY^581/591^ (oxidized form) fluorescence intensities measured by flow cytometry of the 4T1 cells after treatment with PBS, MSPION3, SFN, BQR, SFN + BQR, SFN1@MSPION3, BQR1@MSPION3, or SFN/BQR1@MSPION3 for 24 h. It can be seen that that 4T1 cells exhibit low level of LPO after treatment with PBS, MSPION3, SFN or BQR, moderate level of LPO after treatment with SFN + BQR, SFN1@MSPION3 or SFN1@MSPION3, and high level of LPO after treatment with SFN/BQR1@MSPION3 [39]. 

Given that malondialdehyde (MDA) serves as a reliable indicator for lipid peroxidation (LPO) levels in ferroptosis, the intracellular MDA level was assessed using an MDA kit, thus providing an insight into the extent of lipid peroxidation (Supplementary Fig. 25). The highest expression of MDA was observed for the cells treated with SFN/BQR1@MSPION3 compared with the other groups (** *P* < 0.01) (Supplementary Fig. 25), which was consistent with the LPO results (Fig. 5a-c). Figure 5d-f show CLSM images, green fluorescence distributions and quantitative analysis of the DIO fluorescence intensities measured by flow cytometry for 4T1 cells after different treatments, showing the structural integrity of the cell membrane. It can be seen that the 4T1 cell membranes stained with DIO were almost intact after treatment with PBS, MSPION3, SFN or BQR due to the negligible LPO accumulation (Fig. 5a-c). After treatment with SFN + BQR, SFN1@MSPION3 or BQR1@MSPION3, the membranes of 4T1 cells were broken down and the DIO fluorescence signals were weakened. The SFN/BQR1@MSPION3 treatment resulted in the most severe cell membrane rupture and the weakest DIO fluorescence, which manifested the strong synergistic effect of MSPION3, SFN and BQR on inducing LPO accumulation, i.e., the synergistic ferroptosis effect of MSPION3, SFN and BQR.

### Pharmacokinetics, biodistribution and *T*_2_-Weighted MRI in vivo

Supplementary Fig. 26 shows blood clearance profile of SFN/BQR1@MSPION3 in mice after *i.v.* injection (i.e., pharmacokinetics). The Fe concentration of the blood is steadily reduced over time. The relatively long half-life (t_1/2_ = 7.4 h) of SFN/BQR1@MSPION3 is beneficial for its passive targeting into the tumor location. Supplementary Fig. 27 shows biodistribution of Fe at 1.0, 6.0, 18, 24 and 48 h post-injection (*i.v.*) of SFN/BQR1@MSPION3. The Fe content in major organs (including the heart, liver, spleen, lung, and kidneys) continues to increase within 18 h, but decrease rapidly after 18 h, showing that the SFN/BQR1@MSPION3 is continuously eliminated from the organs. The highest Fe concentration is seen in the liver and spleen, showing that nanoparticles are collected by the reticuloendothelial system (RES) at an early stage. In addition, SFN/BQR1@MSPION3 is constantly enriched in the tumors, reaches its peak at 18 h, and is eliminated slowly from the tumors. This is attributed to the higher permeability and retention characteristics of solid tumor tissues compared to normal tissues, combined with the small particle size of MSPION3 nanoparticles (less than 100 nm). As a result, these nanoparticles can effectively accumulate at tumor sites by the passive targeting, i.e., enhanced permeability and retention (EPR) effect. These findings also lay the foundation for extensive anti-cancer research using MSPION-based DDS.

Supplementary Fig. 28 shows the axial orientation of *T*_2_-weighted MR images for 4T1 tumor-bearing mice pre- and post-injection of SFN/BQR1@MSPION3 (Fe dosage = 5.0 mg/kg) at different time intervals (6, 18, 24, and 48 h), and the corresponding quantitative analysis of the tumor MR images using ΔSNR [40]. The tumor sites show noticeably bright signal before administration of SFN/BQR1@MSPION3 and become dark gradually after the injection of SFN/BQR1@MSPION3. It is obvious that the dark signal of tumors reaches its strongest at 18 h post-injection of SFN/BQR1@MSPION3, which is in accordance with the results of pharmacokinetics and biodistribution. Furthermore, the corresponding quantitative MRI signal intensity at different time points after intravenous injection of SFN/BQR1@MSPION3 is shown in Supplementary Fig. 29. It can be seen that the MRI signal intensity (∼10.2%) at 18 h is significantly lower than that of the 6.0 h post­injection (∼52.7%), suggesting the potential for an ideal *T*_2_-MRI contrast agent due to high accumulation in the tumor sites. Compared with other DDS, nanocarriers with MR imaging capabilities have significant advantages, which allow real-time monitoring of drug accumulation and metabolism at tumor sites and other organs, providing guidance for dosage and injection schedules, and ultimately enhancing the efficacy of anticancer treatment. Unlike pure drugs or carriers without MR imaging functionality, our designed MSPION3 nanoparticles leverage their superparamagnetic properties to offer excellent *T*_2_ imaging capability. However, our MSPION3 only presents *T*_2_ imaging characteristics and lacks *T*_1_ imaging capability. In our future work, we can incorporate Gd or Mn ions to overcome this problem [41]. 

### In vivo anti-tumor efficacy and biosafety of SFN/BQR1@MSPION3

**Fig. 6:**
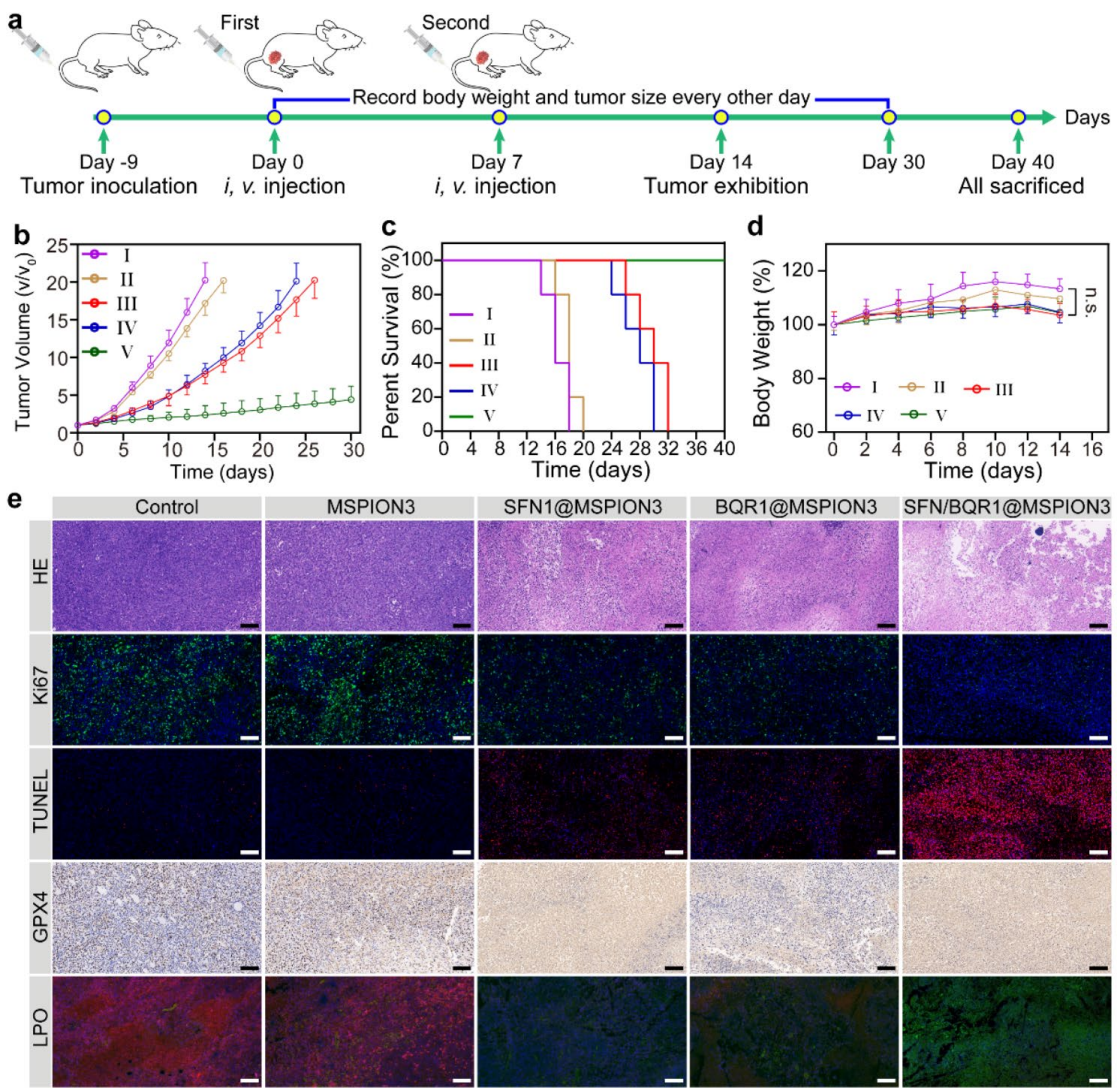
In Vivo Anti-Tumor Efficacy and Biosafety of SFN/BQR1@MSPION3. Anticancer efficacy measured on 4T1 tumor-bearing mice treated with PBS (I), MSPION3 (II), SFN1@MSPION3 (III), BQR1@MSPION3 (IV), and SFN/BQR1@MSPION3 (V) by intravenous injection on day 0 and 7. Fe dosage = 5.0 mg/kg. **a**, Schematic illustration of in vivo therapy for 4T1 subcutaneous tumor-bearing mice. **b, d**, The tumor growth curves (**b**), survival curves (**c**), and body weights (**d**) of 4T1 tumor-bearing mice from each group (*n* = 5). **e**, Representative optical microscopic pictures of sectioned tumor tissue (scale bar: 100 μm) from the five groups of 4T1 tumor-bearing mice after HE, Ki67, TUNEL, GPX4, or LPO staining. **P < 0.01

Encouraged by the anti-cancer potential of SFN/BQR1@MSPION3 nanoparticles in vitro, the tumor therapeutic efficacy in vivo was further assessed. Balb/c mice with 4T1 xenografts were divided into five groups (5 mice per group), and treated with PBS (I), MSPION3 (II), SFN1@MSPION3 (III), BQR1@MSPION3 (IV), and SFN/BQR1@MSPION3 (V), ensuring that each mouse received equivalent Fe dose of 5.0 mg/kg. Figure 6a provides a clear and concise schematic illustration outlining the schedule of tumor inoculation and injection for easy reference and understanding. The tumor growth curves (Fig. 6b) show that free MSPION3 group (II) has a negligible anti-tumor effect, and SFN1@MSPION3 (III) and BQR1@MSPION3 (IV) groups display a moderate tumor growth inhibition efficiency. However, SFN/BQR1@MSPION3 group (V) has superior tumor therapeutic efficacy. The tumor inhibition ratio of group V reaches 88.4% (Supplementary Fig. 30), which is much higher than those of groups I-IV. The PBS and MSPION3 groups resulted in similar tumor growth, which demonstrated the good biocompatibility of our MSPION3 as a generic drug delivery system. Although the Fenton reaction induced by MSPION3 generates toxic •OH, the lipid peroxidation (LPO) generation is inhibited due to the presence of the endogenous antioxidant systems, e.g., the system Xc^−^ and DHODH redox system. Therefore, it is reasonable that MSPION3 alone does not generate ferroptosis of tumor cells. Furthermore, SFN/BQR1@MSPION3 can significantly enhance the survival rate of mice (Fig. 6c). The first mouse death happens at days 14, 16, 26 and 24 for groups I-IV, respectively. However, all the mice survive for group V. The excellent tumor therapeutic efficacy of SFN/BQR1@MSPION3 can be attributed to the synergistic ferroptosis effect of MSPION3, SFN, and BQR. Figure 6d reveals that MSPION3, SFN1@MSPION3, BQR1@MSPION3, and SFN/BQR1@MSPION3 do not cause significant body weight changes compared with group I, indicating the safety of MSPION-based therapy.

The GSH expression in tumors was evaluated by the GSH assay kit at day 14 post-injection (Supplementary Fig. 31). It can be seen that SFN/BQR1@MSPION3 group shows the most exhaustive GSH scavenging efficiency due to the synergistic effect of Fe^3+^ ions, SFN and BQR. At the conclusion of day 14 after injection, tumor tissues from each group were collected and subjected to hematoxylin and eosin (H&E) staining to evaluate the histological changes occurring within the tumors (Fig. 6e). No visible damage in tumor tissues is observed in the group I and II, which varying degrees of damage are found in the groups III and IV, and the most severe damage is observed in the group V, which confirms the excellent tumor therapeutic efficacy of SFN/BQR1@MSPION3.

Figure 6e also shows the representative optical microscopic pictures of sectioned tumor tissue from the five groups of 4T1 tumor-bearing mice after Ki67, TUNEL, GPX4, or LPO staining. Little expression of Ki67 is seen in tumors treated with SFN/BQR1@MSPION3, showing that SFN/BQR1@MSPION3 significantly inhibits the proliferation and differentiation of tumor cells. The evaluation by terminal deoxynucleotidyl transferase (TdT) dUTP Nick-End Labeling (TUNEL) assay further validates that an abundance of apoptotic cells is found in the SFN/BQR1@MSPION3 treated group. In addition, the SFN/BQR1@MSPION3 group shows the most apparent downregulation of GPX4 expression, further revealing the enhanced ferroptosis effect by blocking the ferroptosis reduction system. The tumor tissue after treatment with MSPION3 showed bright ROS fluorescence (Supplementary Fig. 32), indicating the strong Fenton reaction activity of MSPION3. The tumor tissue after treatment with MSPION3 showed weak LPO fluorescence (Fig. 6e) due to the strong endogenous antioxidant systems. However, the tumor tissues after treatment with SFN1@MSPION3 or BQR1@MSPION3 exhibited increased level of LPO, and that after treatment with SFN/BQR1@MSPION3 presented the highest level of LPO. These in vivo results reinforced the synergistic ferroptosis effect of MSPION3, SFN and BQR on LPO accumulation. These results on evaluations of in vivo GSH, GPX4, ROS and LPO reinforce the ferroptosis mechanisms, which are consistent with that evaluated on tumor cells in vitro.

In the 4T1 tumor model, pulmonary metastasis commonly occurs. 4T1 cells are highly metastatic breast cancer cells, known for their ability to generate distant metastases, particularly in the lungs, in mouse models. Therefore, the occurrence of pulmonary metastasis is frequently observed in experiments involving the 4T1 tumor model. In Supplementary Fig. 33, significant amount of tumor metastasis was observed in the lungs of mice in the control group, while mice treated with SFN/BQR1@MSPION3 had no tumor lung metastasis, as SFN/BQR1@MSPION3 inhibits tumor cell proliferation with high ferroptosis efficacy, reducing the risk of lung metastasis.

Supplementary Fig. 34 shows the hemolysis phenomenon, and qualification of the hemolysis rates. Compared with ultrapure water (100%), the hemolysis ratios of MSPION3, SFN1@MSPION3, BQR1@MSPION3, and SFN/BQR1@MSPION3 with the Fe concentration of 300 µg/mL are all less than 4.0%, comparable to saline, demonstrating that the MSPION3-based DDS has outstanding biocompatibility and intravenous injectability.

Various blood biochemical indicators, including alanine aminotransferase (ALT), aspartate aminotransferase (AST), blood urea nitrogen (BUN), creatinine (CRE), and creatine kinase isoenzyme (CK-MB) were determined through biochemical analysis (Supplementary Fig. 35). At two concentrations (i.e., Fe dosage = 5 mg/kg or 15 mg/kg), there is no significant difference between the SFN/BQR1@MSPION3 treated group and the control group. This result demonstrates that SFN/BQR1@MSPION3 has no discernible deleterious impact on the liver, kidney, and heart function of mice even at the Fe dose of 15 mg/kg.

Standard hematological parameters were assessed, including white blood cell count (WBC), red blood cell count (RBC), platelets count (PLT), average red blood cell volume (MCV), mean hemoglobin volume concentration (MCHC), average hemoglobin volume (MCH), hemoglobin concentration (HGB), and hematocrit (HCT) (Supplementary Fig. 36). Compared with the control group, all parameters are normal even at the Fe concentration of 15 mg/kg in the treated group, suggesting that SFN/BQR1@MSPION3 does not cause significant inflammation and infection in the treated mice. Furthermore, upon examination of the major organs (heart, liver, spleen, lung, and kidneys) through hematoxylin and eosin (H&E) staining, no signs of inflammatory lesions or tissue damage were observed in the mice sacrificed after in vivo antitumor therapy (Supplementary Fig. 37), indicating no obvious adverse effects of the tumor therapies utilizing MSPION-based DDS. The low toxicity of MSPION DDS in normal tissues is possibly due to the following two reasons. On the one hand, in the normal physiological environment, the pH of the extracellular matrix is maintained around 7.4, the release of iron ions under such condition is very low, unable to induce effective Fenton reaction to generate cytotoxic ROS to damage normal tissue. On the other hand, the release of SFN and BQR from SFN/BQR1@MSPION3 at neutral pH is also marginal over time. Limited leakage of SFN and BQR causes insignificant toxicity to normal tissues. Furthermore, the clinical translation of SPION-based DDS needs to overcome the following key bottle necks: (1) The production of SPION should be large scale. In this work, MSPION was synthesized using a one-step method with bubble templates, offering a simple process with high potential for large scale production. (2) The drug loading content of SPION should be high. In this study, sorafenib (SFN) and/or brequinar (BQR) were loaded into the MSPION, generating SFN@MSPION, BQR@MSPION and SFN/BQR@MSPION with high drug loading content of 11.5% (SFN), 10.1% (BQR) and 10.0% (SNF + BQR), which demonstrates that our MSPION is a generic DDS for loading of most small molecule drugs (Supplementary Table S3). (3) The biocompatibility of SPION and drug-loaded SPION should be comprehensively evaluated by in vitro and in vivo experiments. In this study, various experiments, such as MTT assay (Fig. S18), AM-PI staining assay (Fig. 3e), apoptosis assay (Fig. 3f), DNA damage assay (Fig. 3g) and blood compatibility assay (Fig. S33), were conducted to demonstrate the excellent in vitro biocompatibility of our MSPION. Additionally, in vivo experiments, including mouse weight change (Fig. 6b), blood biochemistry analysis (Fig. S34), hematology analysis (Fig. S35) and H&E slice of main organs (Fig. S36), also confirmed the good in vivo biocompatibility of our MSPION after loading SFN and/or BQR. (4) The metabolism of SPION in the body must be clearly studied. Blood half-life experiments (Fig. S25) show that our MSPION have a half-life of 7.4 h in the bloodstream. Organ distribution studies (Fig. S26) reveal that MSPION mainly accumulate in the liver and spleen, reaching peak levels at 18 h after intravenous injection. Over time, MSPION can be gradually metabolized, indicating good in vivo biocompatibility.

## Conclusions

SPION have garnered significant attention as MRI contrast agents and DDSs due to their excellent superparamagnetism, large specific surface area, facile surface modification, and fast biodegradability in weakly acidic endosomes and tumor microenvironment (TME). However, direct loading of chemotherapeutic drugs onto the surface of SPION for targeted delivery to tumor site faces the challenge of low drug loading capacity. There have been efforts to develop mesoporous SPION (MSPION) to overcome this limitation. The potential applications of these developed mesoporous SPION for drug loading are limited by their large particle size [14] complicated synthesis process [16], and use of unfriendly surfactants [17]. In this study, we addressed several critical issues. First, proper particle size is crucial. A novel MSPION as a generic DDS was developed in this study. The synthesis of MSPION utilizes a one-step method with bubble template, enabling a simple process with high potential for large-scale production. Remarkably, the size of the SPION can be adjusted by controlling the solvent ratio of diglycol and ethylene glycol without additional surfactants. Second, achieving high drug loading content is essential. SFN and/or BQR were loaded into MSPION, resulting in SFN@MSPION, BQR@MSPION, and SFN/BQR@MSPION with high drug loading contents of 11.5% (SFN), 10.1% (BQR), and 10.0% (SFN + BQR), which demonstrated the versatility of our MSPION as a generic DDS for loading most small molecule drugs. Third, comprehensive evaluation of the biocompatibility through in vitro and in vivo experiments is also a critical aspect. In our study, various experiments such as MTT assay, AM-PI staining assay, apoptosis assay, DNA damage assay, and blood compatibility assay were conducted to demonstrate the excellent in vitro biocompatibility of our MSPION. Furthermore, in vivo experiments including mouse weight change, blood biochemistry analysis, hematology analysis, and histological examination of major organs confirmed the favorable in vivo biocompatibility of our MSPION after loading of SFN and/or BQR. Finally, an understanding of the nanoparticle metabolism in the body is necessary. Blood half-life experiments showed that our MSPION has a half-life of 7.4 h in the bloodstream. Biodistribution studies revealed that our MSPION primarily accumulates in the liver, spleen, and tumors, reaching peak levels at 18 h after intravenous injection. Over time, MSPION can be gradually metabolized, indicating good in vivo biocompatibility. Overall, our MSPION can be used as a generic DDS for tumor therapy.

Finally, to understand the mechanism on the synergistic ferroptosis of MSPION3, SFN and BQR, it is important to comprehend the specific roles of each component. First, MSPION3 can release Fe^2+/3+^ ions in the acidic TME and participate in Fenton reactions to generate a large amount of reactive oxygen species (ROS) (Fig. 4a-c; Supplementary Fig. 32), which serves as the initial driving force for LPO generation. However, the LPO accumulation can be significantly suppressed by the endogenous antioxidant systems, e.g., the system Xc^−^ and DHODH redox system. Second, the released SFN from SFN/BQR1@MSPION3 in the tumor microenvironment (TME) can inhibit the system Xc^−^, and decrease intracellular cystine, GSH and GSH peroxidase 4 (GPX4) levels, thereby enhancing LPO accumulation. Additionally, the released BQR in the TME can further enhance intracellular oxidative stress through dihydroorotate dehydrogenase (DHODH) inhibition to amplify the LPO accumulation. Therefore, the mechanism of synergistic ferroptosis includes the ROS generation from the Fenton reaction induced by MSPION3, and the inhibition of the endogenous antioxidant systems by SFN and BQR.

In addition, the successful synthesis of the optimal MSPION3 was demonstrated by TEM, SEM, DLS, BET, XRD, magnetization curve, XPS, 3.0 and 7.0 T MRI. The strong catalytic activity of MSPION3 on Fenton reaction in acidic environment is reinforced by the TMB assay, DTNB assay, and ESR spectra. The loaded SFN and BQR in MSPION with 1:1 molar ratio can provide synergistic effects with the CI value of 0.06. The release behaviors of SFN and BQR indicate negligible drug leakage in normal tissue environment, and fast drug release for tumor therapy in acidic endosomes and TME. Due to the favorable superparamagnetism of MSPION, SFN/BQR1@MSPION3 is a good *T*_2_-weighted MR contrast agent with a high *r*_2_ values (246.4 mM^− 1^ s^− 1^ at 7.0 T, 186.9 mM^− 1^ s^− 1^ at 3.0 T) and low *r*_1_ values (0.04 mM^− 1^ s^− 1^ at 7.0 T, 0.31 mM^− 1^ s^− 1^ at 3.0 T). The CLSM and flow cytometry results revealed robust cellular uptake and lysosome escape of MSPION3 and SFN/BQR1@MSPION3. The SFN/BQR1@MSPION3 can be used for high performance ferroptosis therapy of tumors because: (1) the released Fe^2+^ and Fe^3+^ in TME can produce highly toxic •OH *via* Fenton reaction; (2) the released SFN in TME can inhibit the System Xc^−^, decrease the intracellular cystine, GSH and GPX4 levels, and thus enhance ROS and LPO levels; (3) the released BQR in TME can further enhance the intracellular oxidative stress *via* DHODH inhibition. The ferroptosis therapeutic mechanism and efficacy are verified on tumor cells in vitro and tumor-bearing mice in vivo. The body weight change after treatments, hemolysis analysis, blood biochemical indicators, standard hematological parameters, and histological analysis by H&E staining all demonstrate the biosafety of MSPION-based DDS.

## Experimental section

### Synthesis of SPION and MSPION

The SPION and MSPION were synthesized through a bubble template method. Typically, iron chloride hexahydrate (FeCl_3_·6H_2_O, 405 mg) and ammonium bicarbonate (NH_4_HCO_3_, 1.185 g) were dissolved into 30 mL of diglycol/ethylene glycol mixed solvents with the volume ratio of 0, 0.23, 0.61, or 1.33. The solutions were dissolved after ultrasound mixing for 10 min, transferred into 50 mL of Teflon-lined autoclave, and then heated at 200 °C for 12 h to obtain SPION1-4. After that, the SPION3 was heated at 250 °C for another 0.1, 12, 72, or 120 h in the Teflon-lined autoclave to obtain MSPION1-4. The black products were collected by centrifugation and washed with ultrapure water and ethanol. The products were finally dried by lyophilization for further use.

### Loading of SFN and BQR into MSPION3

20 mL of MSPION3 dispersion in acetone (1.0 mg/mL) was mixed with 0.50 mL of drug solutions in acetone at different concentrations (SFN: 5.0, 10, or 15 mg/mL, BQR: 4.15, 8.30, or 12.45 mg/mL) under magnetic stirring for 3.0 h. The SFN and/or BQR loaded nanoparticles were then centrifuged and washed two times with Milli-Q water. To measure the drug loading contents and drug loading efficiencies, the drug loaded MSPION3 nanoparticles (SFN@MSPION3, BQR@MSPION3, or SFN/BQR@MSPION3) were dispersed in methanol, followed by ultrasonication for 20 min and centrifuged at 10,000 ×g for 10 min. After that, the supernatants were measured by reverse-phase high-performance liquid chromatography (HPLC, DGU-20 A, SHIMADZU, Japan). The HPLC mobile phase was 500mL mixture of phosphoric acid solution (0.10 M) (250 mL) and acetonitrile (250 mL). The mobile phase was set to flow at a rate of 2.0 mL/min, while the column temperature was carefully maintained at 25 °C throughout the experiment. The SFN was monitored at 265 nm, and the BQR was monitored at 254 nm.

### In vitro fe release from MSPION3

5.0 mL of MSPION3 (2.0 mg/mL) dissolved in PBS at pH 5.0, 6.5, or 7.4 was transferred into a dialysis bag (MwCO: 3500 Da), which was immersed in PBS with the same pH value. The Fe release was conducted under gently shaking (80 rpm) at 37 °C. 1.0 mL of the external solution was collected at 0, 1.0, 3.0, 8.0, 15, 25, 35, 40, 50, 60, or 70 h, and 1.0 mL of fresh PBS was supplemented. The released iron ions in the PBS were measured by an inductively coupled plasma optical emission spectrometry (ICP-OES, iCAP PRO, ThermoFisher Scientific, US).

### Release of SFN and BQR from SFN/BQR1@MSPION3

10 mL of SFN/BQR1@MSPION3 (5.0 mg/mL) dissolved in PBS at pH 5.0, 6.5, or 7.4 was placed into a dialysis bag (MwCO: 10,000 Da), which was immersed in PBS the same pH value. The drug release was conducted under gently shaking (80 rpm) at 37 °C. 1.0 mL of the external solution was collected at 0, 1.0, 3.0, 5.0, 10, 15, 20, 25, 35, 45, 60–70 h, and 1.0 mL of fresh PBS was supplemented. The released SFN or BQR was finally measured by HPLC.

### Cell culture

4T1 cells were cultured in a humidified atmosphere with 5.0% CO_2_ at 37 °C in DMEM medium supplemented with 10 wt% fetal bovine serum (FBS) and 100 units/mL penicillin and streptomycin, ensuring optimal conditions for cell growth.

### Tumor model

All procedures involving animals were conducted following the ethical guidelines outlined in the Guidelines for Care and Use of Laboratory Animals of Southern Medical University, and were approved by the Animal Ethics Committee of Southern Medical University. The study was assigned the approval/accreditation number SYXK(YUE)2021 − 0167.

To establish the xenograft tumor models, female Balb/c mice (5-weeks old, 15–20 g) were subcutaneously implanted with 4.0 × 10^6^ of 4T1 cells in the right back side. The size of tumors was measured every other day with a vernier caliper, and the tumor volumes were calculated as follows: tumor volume (mm^3^) = width^2^ × length / 2. Regarding the subsequent treatment plan, which includes tumor growth curve, mouse survival curve, and pulmonary metastasis assessment, “Day 0” refers to the day of tumor inoculation.

### Electronic supplementary material

Below is the link to the electronic supplementary material.

Supplementary Material 1

## Data Availability

The data that support the findings of this study are available from the corresponding author upon reasonable request.
