# Supplementary material for: A mesoporous superparamagnetic iron oxide nanoparticle as a generic drug delivery system for tumor ferroptosis therapy
Source: J Nanobiotechnology. 2024 Apr 24;22:204. doi: 10.1186/s12951-024-02457-w (PMC11044424; doi:10.1186/s12951-024-02457-w)
Supplement: Supplementary file 1 — Supplementary Material 1 [file 12951_2024_2457_MOESM1_ESM.docx]

Supporting Information

**A Mesoporous Superparamagnetic Iron Oxide Nanoparticle as a Generic Drug Delivery System for Tumor Ferroptosis Therapy**

Jing Yang, Wei Xiong, Lin Huang, Zongheng Li, Qingdeng Fan, Fang Hu, Xiaopin Duan, Junbing Fan, Bo Li, Jie Feng, Yikai Xu, Xiaoyuan Chen***, Zheyu Shen*

**This file includes:**

Experimental Section.

Table S1. Synthesis conditions of SPION1-4.

Table S2. Synthesis conditions of MSPION1-4.

Table S3. Synthesis conditions and characterization results of SFN1-3@MSPION3, BQR1-3@MSPION3, and SFN/BQR1-2@MSPION3.

Figure S1. Photographs of the purified SPION1-4 and MSPION1-4.

Figure S2. The SEM image of MSPION3.

Figure S3. Energy Dispersive X-ray Spectroscopy (EDS) spectrum of MSPION3.

Figure S4. Magnetization Vs Magnetic Field M-H plot or ZFC/FC curve of MSPION3.

Figure S5. The X-ray photoelectron spectroscopy (XPS) spectrum of MSPION3.

Figure S6. UV-vis spectra of the DTNB solutions to testify the GSH consumption capacities.

Figure S7. OPD colorimetric method for the measurement of •OH generation.

Figure S8. High performance liquid chromatography (HPLC) measurement for SFN, or BQR.

Figure S9. 4T1 cell viability treated by SFN or SFN plus BQR.

Figure S10. Zeta potentials of various nanoparticles.

Figure S11. Dynamic light scattering (DLS) size distributions of various nanoparticles.

Figure S12. Dynamic light scattering (DLS) size distributions of MSPION3 in DMEM+10% FBS solution.

Figure S13. Release curves of SFN or BQR.

Figure S14. *T*_2_ -weighted MR image, and *T*_1_ relaxation rate of SFN1/BQR1@MSPION3.

Figure S15. CLSM images of 4T1 cells showing intracellular uptake of pure R6G.

Figure S16. Fluorescence distributions and quantitative analysis of 4T1 cells treated with R6G@SFN/BQR1@MSPION3.

Figure S17. 4T1 cell viabilities incubated with various concentration of MSPION3.

Figure S18. SSC/FSC strategy applied in 4T1 cells apoptosis detection.

Figure S19. The early and total apoptosis statistics of 4T1 cells.

Figure S20. CLSM images of 4T1 cells about DNA damage.

Figure S21. CLSM images of 4T1 cells, showing intracellular Fe^2+^ outside lysosomes.

Figure S22. The GSH level in 4T1 cells measured by DTNB method.

Figure S23. CLSM images of 4T1 cells, showing intracellular GSH level.

Figure S24. CLSM images of 4T1 cells, showing mitochondrial membrane potential change.

Figure S25. Relative MDA level of 4T1 cells after various treatments.

Figure S26. Blood clearance profile of SFN/BQR1@MSPION3.

Figure S27. Biodistribution of Fe post-injection (*i.v.*).

Figure S28. *T*_2_-weighted MR images of 4T1 tumor-bearing mice pre- and post-injection of SFN/BQR1@MSPION3.

Figure S29. The corresponding quantitative analysis of the tumor MR images in Fig. S26.

Figure S30. The tumor inhibition ratio for the 4T1 tumor-bearing mice.

Figure S31. Evaluations of the GSH level in tumors on day 14 after treatments.

Figure S32. Fluorescence pictures of sectioned tumor tissues after ROS staining with DCFH-DA.

Figure S33. Representative lung photographs in 4T1 tumor-bearing mice on day 14 post-treatment.

Figure S34. Hemolysis photo and qualification of the hemolysis rates for various nanoparticles.

Figure S35. Blood biochemistry analysis of SFN/BQR1@MSPION3.

Figure S36. Hematology analysis of SFN/BQR1@MSPION3.

Figure S37. The H&E-stained main organs (liver, heart, spleen, lung and kidney).

**EXPERIMENTAL SECTION**

**Materials and reagents:** NH_4_HCO_3_, FeCl_3_·6H_2_O, ethylene glycol (EG), hydrogen peroxide (H_2_O_2_, 30%), phosphoric acid, o-phenylenediamine (OPD), and Rhodamine (R6G) were purchased from Sigma-Aldrich (USA). Fe^2+^ indicator (RhoNox-1) was purchased from Shanghai Maokang Biotechnology Co., Ltd. 3-(4,5-dimethylthiazol-2-yl)-2,5-diphenyltetrazolium bromide (MTT) and ferrostatin-1 (Fer-1) were purchased from Sigma-Aldrich. 3,3’,5,5’-Tetramethylbenzidine (TMB) was bought from Shanghai Macklin Biochemical Co., Ltd. 5,5’-Dithiobis (2-nitrobenzoic acid) (DTNB) was obtained from Shanghai Dibai Chemicals Technology Co., Ltd. (Shanghai, China). Glutathione (GSH) was purchased from Shanghai Reagent Chemical Co. (Shanghai, China). Dulbecco’s modified Eagle’s medium (DMEM), penicillin-streptomycin, fetal bovine serum (FBS), and trypsin were purchased from Invitrogen. DCFH-DA, Endo/Lyso-Tracker Green, Endo/Lyso-Tracker Red, FITC-Phalloidin, cell plasma membrane staining probe (DIO), calcein acetoxymethyl ester (Calcein-AM)/Propidium iodide (PI) Assay Kit, 5,5’,6,6’-tetrachloro-1,1’,3,3’-tetraethylbenzimidazolylcarbocyanine iodide (JC-1) Assay Kit, Annexin V-FITC/Propidium Iodide (PI) Apoptosis Detection Kit, GSH Assay Kit, malondialdehyde (MDA) assay, and glutathione peroxidase 4 (GPX4) ELISA kit were purchased from Beyotime Biotechnology Co. (Shanghai, China). C11 BODIPY^581/591^ was purchased from Shanghai Mao Kang Biotechnology Co., Ltd.

**Characterizations:** Transmission electron microscope (TEM) and scanning electron microscope (SEM) were used to observe the morphology and structure of the nanoparticles. X-ray diffraction (XRD) patterns were obtained with the parameters set as Cu Kα, 40 mA, and 40 kV. X-ray photoelectron spectroscopy (XPS) was utilized to determine the valence states of elements for the nanoparticles. The hydrodynamic sizes and surface charges of the nanoparticles were measured using a Zeta Potential analyzer (Brookhaven, 900 Plus Zeta). N_2_ adsorption-desorption isotherms were obtained from a Micromeritics ASAP 2020M automated sorption analyzer to measure the specific surface area and pore sizes. The Fe contents of the nanoparticles was determined by inductively coupled plasma (ICP) analysis. The hysteresis loop and the zero-field cooling (ZFC) and field cooling (FC) experiments were conducted utilizing the Physical Property Measurement System (PPMS, PPMS-EverCool, Quantum Design, USA). The hysteresis loop experiment was performed at a temperature of 300 K for the nanoparticles powder. The ZFC and FC experiments were conducted on the nanoparticles powder with a heating rate of 12 K/min under an applied magnetic field of 100 Oe.

**Fenton reaction monitoring for MSPION3:** A 3,3,5,5-tetramethylbenzidine (TMB) colorimetric method is used to monitor the Fenton reaction. Briefly, 5.0 μg/mL MSPION3 was incubated with 0.40 mM of TMB and/or 20 mM of H_2_O_2_ at pH 5.0, 6.5, or 7.4 for 0, 3.0, 8.0, 25, 50, or 70 h, respectively. After that, UV-vis spectroscopy was used to monitor the Fenton reaction at different conditions. To eliminate the influence of UV-vis absorption by MSPION3 solution across the entire spectrum, it is necessary to centrifuge and remove the MSPION3 nanoparticles under the same conditions when measuring the absorption peak of the TMB solution.

An o-phenylenediamine (OPD) colorimetric method is used to monitor the Fenton reaction. Briefly, 5.0 μg/mL MSPION3 was incubated with 0.2 mM of OPD and/or 20 mM of H_2_O_2_ at pH 5.0, 6.5, or 7.4 for 70 h, respectively. After that, UV-vis spectroscopy was used to monitor the Fenton reaction at different conditions.

Moreover, the electron spin resonance (ESR) measurement was conducted to confirm the generation of •OH using 5,5-dimethyl-1-pyrrolineNoxide (DMPO) as the trapping agent. 10 μL of DMPO was mixed with 100 μL of MSPION3 (0.010 mM [Fe]), which was dispersed in PBS at pH 5.0, 6.5, or 7.4 for 70 h. After culture, the PBS containing 1.0 mM of H_2_O_2_ was added. After 30 min of reaction, the ESR measurements was carried out.

***In vitro* MRI performance:** The MRI contrast potential of SFN/BQR1@MSPION3 was explored on MRI scanner systems (3.0 T, Philips, Ingenia, NL; 7.0 T, Bruker, US). SFN/BQR1@MSPION3 was dispersed in PBS solutions (pH 7.4, 6.5, or 5.0) with different Fe concentrations (200, 100, 50.0, 25.0, 12.5, or 6.25 μM). The dispersions were incubated for 24 h at 37 ^o^C, and then were placed in Eppendorf tubes (1.0 cm in diameter) for MRI test. The relaxivity value of *r*_2_ or *r*_1_ was obtained from the slope of the linear curve of relaxation rate (1/*T*_2_ or 1/*T*_1_, s^-1^) versus Fe concentration (mM). For *T*_2_ relaxation rates at magnetic field of 7.0 T, TR is 6000 ms, and TE is 120 ms. For *T*_2_ relaxation rates at magnetic field of 3.0 T: TR is 5000 ms, and TE is 80 ms. For *T*_1_ relaxation rates at magnetic field of 7.0 T: TR is 75.8 ms, and TE is 6.0 ms. For *T*_1_ relaxation rates at magnetic field of 3.0 T: TR is 200 ms, TE is 8.2 ms.

**GSH consumption by SFN/BQR1@MSPION3:** For evaluation of GSH depletion capability, 50 µL of SFN/BQR1@MSPION3 (10 mg/mL) was mixed with 5.0 mL of PBS containing 1.0 mM of GSH at pH 7.4, 6.5, or 5.0. The mixed solutions were maintained at 37 °C under magnetic stirring for 6.0, 12, or 24 h. The above mixtures were then centrifuged at 10000 g for 5.0 min. The obtained supernatants were further mixed with 50 μL of DTNB (10 mg/mL), and incubated for 15 min. After that, the solutions were measured by UV-vis spectrophotometer at the wavelength of 412 nm.

**MTT assay:** Cytotoxicities were evaluated using the methyl thiazolyl tetrazolium (MTT) assay in 4T1 cells. Typically, 4T1 cells were seeded in a 96-well culture plate at a density of 1.0 × 10^4^ cells per well. To verify the optimal ratio of SFN and BQR, the drugs with a range of different ratios were added into the culture medium. Subsequently, the PBS, MSPION3 (*C*_Fe_ = 10 μg/mL), SFN1@MSPION3 (*C*_Fe_ = 10 μg/mL), BQR1@MSPION3 (*C*_Fe_ = 10 μg/mL), or SFN/BQR1@MSPION3 (*C*_Fe_ = 10 μg/mL) in 100 μL of culture media were used to replace the previous medium. After 24 h of incubation, 100 μL of fresh culture media was used to replace the previous culture media, followed by addition of 10 μL MTT (5.0 mg/mL in PBS). After 4.0 h, the culture media were replaced with 150 μL of DMSO per well to dissolve the formazan. Finally, a multi-mode microplate reader (Synergy H1, BioTek Instruments, USA) was applied to monitor the absorbance at 495 nm.

Furthermore, to test the synergistic effect of MSPION3, SFN, and BQR, the PBS, MSPION3 (*C*_Fe_ = 10 μg/mL), SFN (equivalent concentration of 0.70 μg/mL), BQR (equivalent concentration of 0.57 μg/mL), SFN + BQR (0.7 μg/mL of SFN + 0.57 μg/mL of BQR), or SFN/BQR1@MSPION3 (*C*_Fe_ = 10 μg/mL) in 100 μL of culture media were used to replace the previous medium. The cells were then incubated for 24 h. The other steps were same with the above-mentioned procedures for MTT assay.

To verify the ferroptosis inducibility of SFN/BQR1@MSPION3, the 4T1 cells were treated with SFN/BQR1@MSPION3 (*C*_Fe_ = 10 μg/mL). After 12 h of incubation, the culture medium containing nanoparticles was replaced by 100 μL of complete culture medium or culture DMEM medium containing Fer-1 (2.0 μM), DFO (100 μM), or NAC (2.0 mM). The cells were then incubated for another 12 h. The other steps were same with the above-mentioned procedures for MTT assay.

**R6G Labeling of Nanoparticles:** 4.0 mL of MSPION3, or SFN/BQR1@MSPION3 (*C*_Fe_ = 3.0 mM) were mixed with 0.70 mL of Rhodamine 6G (10 μM) under magnetic stirring at room temperature. After 24 h, the R6G@ MSPION3 or R6G@SFN/BQR1@MSPION3 was obtained by centrifugation (15000 × g, 10 min) and washing with pure water for further use.

**Cellular uptake:** For confocal laser scanning microscope (CLSM) analysis, 4T1 cells (1.0 × 10^5^ cells) were first seeded into the confocal dishes, and incubated overnight to achieve adherence. Next, the growth media were replaced with DMEM medium (0.50 mL) containing R6G@MSPION3 or R6G@SFN/BQR1@MSPION3 (*C*_Fe_ = 5.0 μg/mL), and the cells were incubated for 4.0 h. The treated cells were washed with PBS, fixed with 4.0 % of paraformaldehyde for 30 min, permeabilized with 0.10 % Triton X-100 for 5.0 min, blocked with 1.0 % BSA for 30 min. The cells were then stained with DAPI and FITC-Phalloidin for 30 min. Finally, the cells were observed by CLSM (Nikon ECLIPSE Ti2).

For flow cytometry analysis, 4T1 cells were first seeded into 6-well plates (5.0 × 10^5^ cells/well) and incubated overnight to achieve adherence. Then, the culture medium was replaced with DMEM medium (1.0 mL) containing R6G@MSPION3 or R6G@SFN/BQR1@MSPION3 (*C*_Fe_ = 5.0 μg/mL). After incubation for further 2.0, 4.0, or 8.0 h, the cells were washed twice with cold PBS, and harvested by trypsinization and centrifugation (1000 × g, 5.0 min). The obtained cells were re-suspended in PBS, and analyzed using flow cytometry.

**Observation of lysosomal escape:** 4T1 cells (1.0 × 10^5^ cells/well) were seeded into the confocal dishes and cultured overnight. The culture media were then replaced with fresh media (1.0 mL) without or with R6G@ MSPION3, or R6G@SFN/BQR1@MSPION3 (*C*_Fe_ = 5.0 μg/mL) for 4.0 h. The treated cells were washed with PBS, fixed with 4.0 % of paraformaldehyde for 30 min, permeabilized with 0.10 % Triton X-100 for 5.0 min, and blocked with 1.0 % BSA for 30 min. The cells were then washed with PBS twice, stained with DAPI for 30 min and Lysotracker Green for 1.0 h. Finally, the cells were observed by CLSM (Nikon ECLIPSE Ti2).

**Calcein-AM/PI assay:** 4T1 cells (1.0 × 10^5^ cells) were first seeded into the confocal dishes and incubated overnight to achieve adherence. The culture media were then replaced with fresh one without or with MSPION3 (*C*_Fe_ = 10 μg/mL), SFN1@MSPION3 (*C*_Fe_ = 10 μg/mL), BQR1@MSPION3 (*C*_Fe_ = 10 μg/mL), or SFN/BQR1@MSPION3 (*C*_Fe_ = 10 μg/mL). After 24 h of culture, the cells were washed several times with PBS, stained with Calcein-AM (final concentration: 2.0 μM) and PI (final concentration: 3.0 μg/mL) at 37 ^o^C for 30 min. The cells were finally observed by CLSM.

To test the synergistic effect of MSPION3, SFN, and BQR, the culture media were then replaced with fresh one without or with MSPION3 (*C*_Fe_ = 10 μg/mL), SFN (equivalent concentration of 0.70 μg/mL), BQR (equivalent concentration of 0.57 μg/mL), SFN + BQR (0.7 μg/mL of SFN + 0.57 μg/mL of BQR), or SFN/BQR1@MSPION3 (*C*_Fe_ = 10 μg/mL). The cells were then incubated for 24 h. The other steps were same with the above-mentioned procedures for calcein-AM/PI assay.

**Cell apoptosis assay:** 4T1 cells were first seeded into 6-well plates (5.0 × 10^5^ cells/well), and incubated overnight to achieve adherence. The culture media were then replaced with fresh one without or with MSPION3 (*C*_Fe_ = 10 μg/mL), SFN1@MSPION3 (*C*_Fe_ = 10 μg/mL), BQR1@MSPION3 (*C*_Fe_ = 10 μg/mL), or SFN/BQR1@MSPION3 (*C*_Fe_ = 10 μg/mL). After 24 h of culture, the cells were harvested by trypsinization and centrifugation (1000 × g, 5.0 min). The cells were treated using an Annexin V-FICT/PI apoptosis detection kit (C1062S-2, Beyotime, China) following the manufacturer’s protocol. Finally, the cell apoptosis rate was measured by flow cytometry. The SSC (Side Scatter)/FSC (Forward Scatter) gating condition was used to select intact cells and exclude debris and cell aggregates. The SSC/FSC plot was typically used to define a region that contains the population of intact cells. Each run of the assay included all formulations. This approach ensured that all groups were tested under the same experimental conditions, allowing for accurate comparisons and analysis. Voltage adjustments were typically made to optimize the separation of populations and achieve an appropriate signal-to-noise ratio.

**Detection of intracellular ferrous ions:** 4T1 cells were seeded into confocal dishes with a density of 1.0 × 10^5^ cells per dish. After adherence, the culture media were replaced with fresh one without or with MSPION3 (*C*_Fe_ = 10 μg/mL), SFN1@MSPION3 (*C*_Fe_ = 10 μg/mL), BQR1@MSPION3 (*C*_Fe_ = 10 μg/mL), or SFN/BQR1@MSPION3 (*C*_Fe_ = 10 μg/mL). After 6.0 h of culture, the cells were washed with PBS, fixed with 4.0 % of paraformaldehyde for 30 min, permeabilized with 0.10 % Triton X-100 for 5.0 min, blocked with 1.0 % BSA for 30 min. The cells were then stained with DAPI for 30 min, Endo/Lyso-Tracker Red for 30 min and RhoNox-1 (5.0 μM) for 30 min. After that, the cells were washed by PBS and observed by CLSM.

**Evaluation of intracellular ROS:** ROS probe DCFH-DA was utilized to evaluate intracellular ROS levels. For CLSM analysis, 4T1 cells (1.0 × 10^5^ cells) were first seeded into the confocal dishes and incubated overnight to achieve adherence. Next, the growth media were replaced with fresh one without or with MSPION3 (*C*_Fe_ = 10 μg/mL), SFN1@MSPION3 (*C*_Fe_ = 10 μg/mL), BQR1@MSPION3 (*C*_Fe_ = 10 μg/mL), or SFN/BQR1@MSPION3 (*C*_Fe_ = 10 μg/mL). After 4.0 h of incubation, the cells were washed with PBS, and incubated in the culture media containing DCFH-DA (10 μM). After further incubation for 30 min, the cells were washed with PBS and observed by CLSM.

For flow cytometry analysis, 4T1 cells were first seeded into 6-well plates (5.0 × 10^5^ cells/well) and incubated overnight to achieve adherence. Then, the culture medium was replaced with fresh one without or with MSPION3 (*C*_Fe_ = 10 μg/mL), SFN1@MSPION3 (*C*_Fe_ = 10 μg/mL), BQR1@MSPION3 (*C*_Fe_ = 10 μg/mL), or SFN/BQR1@MSPION3 (*C*_Fe_ = 10 μg/mL). After 4.0 h of incubation, the cells were washed with PBS, and incubated in the culture media containing DCFH-DA (10 μM). After further incubation for 30 min, the cells were washed twice with cold PBS and harvested by trypsinization and centrifugation (1000 × g, 5.0 min). The obtained cells were finally analyzed using flow cytometry.

**Detection of intracellular GSH:** 4T1 cells were seeded in a 6-well plate (5.0 × 10^5^ cells/well) and cultured overnight to achieve adherence. The culture medium was replaced with fresh one without or with MSPION3 (*C*_Fe_ = 10 μg/mL), SFN1@MSPION3 (*C*_Fe_ = 10 μg/mL), BQR1@MSPION3 (*C*_Fe_ = 10 μg/mL), or SFN/BQR1@MSPION3 (*C*_Fe_ = 10 μg/mL). After 24 h of incubation, the cells were washed with cold PBS and collected with a cell scraper. Subsequently, the cells were lysed for 20 min with Triton X-100 lysis buffer, and centrifuged for 10 min (1000 × g, 4.0 ^o^C). The supernatant (50 μL) was mixed with 200 μL of DTNB (200 μM) in a 96-well plate. After 30 min of reaction at 37 ^o^C, the absorbance was measured by a multi-mode microplate reader (Synergy H1, BioTek Instruments, USA) at 412 nm.

For CLSM analysis, 4T1 cells (1.0 × 10^5^ cells) were first seeded into confocal dishes and incubated overnight to achieve adherence. The culture medium was replaced with fresh one without or with MSPION3 (*C*_Fe_ = 10 μg/mL), SFN1@MSPION3 (*C*_Fe_ = 10 μg/mL), BQR1@MSPION3 (*C*_Fe_ = 10 μg/mL), or SFN/BQR1@MSPION3 (*C*_Fe_ = 10 μg/mL). After 24 h of incubation, the treated cells were washed with PBS, fixed with 4.0 % of paraformaldehyde for 30 min, permeabilized with 0.10 % Triton X-100 for 5.0 min, blocked with 1.0 % BSA for 30 min. The cells were then stained with DAPI for 30 min, and Thiol Tracker Violet kit (10 μM) for 30 min. Finally, the cells were observed by CLSM (Nikon ECLIPSE Ti2).

**Measurement of intracellular GPX4 activity:** The GPX4 activity was measured by the enzyme-linked immunosorbent assay (ELISA) kit (S0058, Beyotime, China). Briefly, 4T1 cells were seeded in 6-well plates at a density of 5.0 × 10^5^ cells/well to achieve adherence. The culture medium was replaced with fresh one without or with MSPION3 (*C*_Fe_ = 10 μg/mL), SFN1@MSPION3 (*C*_Fe_ = 10 μg/mL), BQR1@MSPION3 (*C*_Fe_ = 10 μg/mL), or SFN/BQR1@MSPION3 (*C*_Fe_ = 10 μg/mL). After 24 h of incubation, the GPX4 activity was measured according to the manufacturer’s protocol using a multi-mode microplate reader (Synergy H1, BioTek Instruments, USA).

**Detection of mitochondrial membrane potential:** The mitochondrial membrane potential was detected using JC-1 probe (C2006-2, Beyotime, China). Briefly, 4T1 were seeded in a confocal dish with 1.0 × 10^5^ cells/dish to achieve adherence. The culture medium was replaced with fresh one without or with MSPION3 (*C*_Fe_ = 10 μg/mL), SFN1@MSPION3 (*C*_Fe_ = 10 μg/mL), BQR1@MSPION3 (*C*_Fe_ = 10 μg/mL), or SFN/BQR1@MSPION3 (*C*_Fe_ = 10 μg/mL). After 24 h of incubation, the cells were washed with PBS, and stained with the JC-1 (10 μg/mL) probe for 30 min. Then cells were then rinsed thrice with PBS, and observed by CLSM. A green channel with a 488 nm of excitation wavelength was utilized for JC-1 monomers, while a red channel with a 546 nm of excitation wavelength was utilized for JC-1 aggregates.

**Evaluation of intracellular lipid peroxides (LPO) level:** For CLSM analysis, 4T1 cells (1.0 × 10^5^ cells) were first seeded into confocal dishes, and incubated overnight to achieve adherence. Next, the culture media were replaced with fresh one without or with MSPION3 (*C*_Fe_ = 10 μg/mL), SFN (equivalent concentration of 0.70 μg/mL), BQR (equivalent concentration of 0.57 μg/mL), SFN + BQR@MSPION3 (0.7 μg/mL of SFN + 0.57 μg/mL of BQR), SFN1@MSPION3 (*C*_Fe_ = 10 μg/mL), BQR1@MSPION3 (*C*_Fe_ = 10 μg/mL), , SFN/BQR1@MSPION3 (*C*_Fe_ = 10 μg/mL). After 24 h of incubation, the cells were washed with PBS, fixed with 4.0 % of paraformaldehyde for 30 min, permeabilized with 0.10 % Triton X-100 for 5.0 min, blocked with 1.0 % BSA for 30 min. The cells were then stained with DAPI for 30 min, and BODIPY-C11^581/591^ (5.0 μM) for 30 min. Finally, the cells were observed by CLSM (Nikon ECLIPSE Ti2).

For flow cytometry analysis, 4T1 cells were first seeded into 6-well plates (5.0 × 10^5^ cells/well), and incubated overnight to achieve adherence. Then, the culture media were replaced with fresh one without or with MSPION3 (*C*_Fe_ = 10 μg/mL), SFN (equivalent concentration of 0.70 μg/mL), BQR (equivalent concentration of 0.57 μg/mL), SFN + BQR@MSPION3 (0.7 μg/mL of SFN + 0.57 μg/mL of BQR), SFN1@MSPION3 (*C*_Fe_ = 10 μg/mL), BQR1@MSPION3 (*C*_Fe_ = 10 μg/mL), , SFN/BQR1@MSPION3 (*C*_Fe_ = 10 μg/mL). After 24 h of incubation, the cells were then stained with BODIPY-C11^581/591^ (5.0 μM) for 30 min. The cells were washed twice with PBS, and harvested by trypsinization and centrifugation (1000 × g, 5.0 min). The obtained cells were finally re-suspended in PBS, and analyzed using flow cytometry.

**Measurement of intracellular MDA expression:** 4T1 cells were seeded into 6-well plates (5.0 × 10^5^ cells/well) and incubated overnight to achieve adherence. The culture media were replaced with fresh one without or with MSPION3 (*C*_Fe_ = 10 μg/mL), SFN (equivalent concentration of 0.70 μg/mL), BQR (equivalent concentration of 0.57 μg/mL), SFN + BQR@MSPION3 (0.7 μg/mL of SFN + 0.57 μg/mL of BQR), SFN1@MSPION3 (*C*_Fe_ = 10 μg/mL), BQR1@MSPION3 (*C*_Fe_ = 10 μg/mL), , SFN/BQR1@MSPION3 (*C*_Fe_ = 10 μg/mL). After 24 h of incubation, the cells were washed with Dulbecco’s Phosphate Buffered Saline (DPBS), digested, and disrupted with ultrasonication. The supernatants were collected by centrifugation at 10000 rpm for 10 min at 4.0 ^o^C. The absorbance of the supernatant was measured at 450, 532, or 600 nm, respectively, by a microplate reader for the calculation of MDA level according to the manufacturer’s protocol (Beyotime).

**Observation of cell membrane integrity:** 4T1 cells were seeded into confocal dishes (5.0×10^5^ cells/well) and incubated for 24 h. The culture media were replaced with fresh one without or with MSPION3 (*C*_Fe_ = 10 μg/mL), SFN (equivalent concentration of 0.70 μg/mL), BQR (equivalent concentration of 0.57 μg/mL), SFN + BQR@MSPION3 (0.7 μg/mL of SFN + 0.57 μg/mL of BQR), SFN1@MSPION3 (*C*_Fe_ = 10 μg/mL), BQR1@MSPION3 (*C*_Fe_ = 10 μg/mL), , SFN/BQR1@MSPION3 (*C*_Fe_ = 10 μg/mL). After 24 h of incubation, the cells were washed with PBS, fixed with 4.0 % of paraformaldehyde for 30 min, permeabilized with 0.10 % Triton X-100 for 5.0 min, blocked with 1.0 % BSA for 30 min. The cells were then stained with DAPI for 30 min, and DIO (1.0 mM) for 30 min. Finally, the cells were observed by CLSM (Nikon ECLIPSE Ti2).

For flow cytometry analysis, 4T1 cells were seeded into 6-well plates (5.0×10^5^ cells/well), and incubated for 24 h. The culture media were replaced with fresh one without or with MSPION3 (*C*_Fe_ = 10 μg/mL), SFN (equivalent concentration of 0.70 μg/mL), BQR (equivalent concentration of 0.57 μg/mL), SFN + BQR@MSPION3 (0.7 μg/mL of SFN + 0.57 μg/mL of BQR), SFN1@MSPION3 (*C*_Fe_ = 10 μg/mL), BQR1@MSPION3 (*C*_Fe_ = 10 μg/mL), , SFN/BQR1@MSPION3 (*C*_Fe_ = 10 μg/mL). After 24 h of incubation, the cells were cultured with DIO (1.0 mM) for 30 min. The cells were washed twice with PBS, and harvested by trypsinization and centrifugation (1000 × g, 5.0 min). The obtained cells were re-suspended in PBS, and analyzed using flow cytometry.

***In vivo* MRI performance:** For MRI of tumors, 4T1-bearing mice were anesthetized and injected with SFN/BQR1@MSPION3 suspension (Fe dosage = 5.0 mg/kg) *via* the tail vein. The *T*_2_-weighted MR images were acquired by using a 7.0 T Bruker MRI scanner at the determined time points (0, 6.0, 18, 24, or 48 h) after injection. Meanwhile, the signal intensities of tumors were measured on the Image J software. The signal-to-noise ratio (SNR) and relative intensity values were calculated according to the equation (1) and (2).

SNR = SI_mean_/SD_noise_ (1)

Relative intensity = SNR_post_/SNR_pre_ × 100 % (2)

***In vivo* biodistribution:** When the tumor volume of 4T1 tumor-bearing mice grew up to 100-150 mm^3^, the mice were randomly divided into five groups (n = 3 per group) and then intravenously injected with 0.10 mL of SFN/BQR1@MSPION3 at Fe dosage of 5.0 mg/kg. At 1.0, 6.0, 18, 24, or 48 h post-injection, the mice were sacrificed. Subsequently, their blood, major organs (heart, liver, spleen, lung, and kidney) and tumors were collected and completely digested with concentrated nitric acid. Fe contents were determined by ICP-MS, and expressed as a percentage of the injected Fe dose per gram of tissue.

***In Vivo* Pharmacokinetics:** For the pharmacokinetic analysis, 4T1 tumor-bearing mice were used. Before injection of SFN/BQR1@MSPION3 nanoparticles, 0.1 mL of blood (as a control to eliminate the signal of iron ions inside blood) was collected from each mouse, and an equal volume of saline solution was immediately injected *via* the tail vein. Subsequently, 0.1 mL SFN/BQR1@MSPION3 nanoparticles (with an Fe dosage of 5.0 mg/kg) were intravenously injected *via* the tail vein. Following injection, venous blood samples (0.1 mL each time) were collected from the orbital region at different time points, and an equal volume of saline solution was immediately supplemented *via* the tail vein after each blood drawing. Finally, the samples were analyzed for Fe concentration using inductively coupled plasma optical emission spectrometry (ICP-OES).

**Tumor therapy performance:** To investigate the efficacy of SFN/BQR1@MSPION3 mediated ferroptosis therapy *in vivo*, 4T1 cancer-bearing mice were randomly divided into five groups for the following treatments when the tumor volume reached to about 100 mm^3^: I) PBS; II) MSPION3; III) SFN1@MSPION3; IV) BQR1@MSPION3; V) SFN/BQR1@MSPION3. 100 μL of PBS, MSPION3 (Fe dosage = 5.0 mg/kg), SFN1@MSPION3 (Fe dosage = 5.0 mg/kg), BQR1@MSPION3 (Fe dosage = 5.0 mg/kg), or SFN/BQR1@MSPION3 (Fe dosage = 5.0 mg/kg) were intravenously injected into the 4T1 tumor-bearing mice at the day of 0 and 7, respectively. Body weights and tumor volumes of mice in each group were recorded every other day during the treatment (the total tumor volume should be smaller than 2000 mm^3^).

In another parallel study, mice were sacrificed on day 14 following the treatments. Tumor weight was measured, histological analysis of the tumors was conducted, and the lungs were collected for observation. The tumor tissues of each group were excised, fixed in 4.0 % of paraformaldehyde, embedded in paraffin, sliced and stained with hematoxylin and eosin (H&E). Tumor sections were also stained with TUNEL apoptosis, Ki67 detection kit assay, C11 BODIPY^581/591^, and DCFH-DA reagent, respectively. In addition, the tumor issues were also stained by GPX4 primary and secondary antibodies using the immunohistochemical method. Finally, those tumor sections were observed by CLSM. As for GSH detection, tumors were harvested on day 14 after treatments, and analyzed by the guidance of the commercial GSH assay kits.

**Hemolysis assay:** The hemocompatibility of MSPION3, SFN1@MSPION3, BQR1@MSPION3, and SFN/BQR1@MSPION3 was also studied. First, blood (2.0 mL) was collected from the healthy BALB/c mice through the eyeball. Afterward, the fibrinogen was removed. The defibrillated blood constituents were diluted with PBS, and then centrifuged (400 g) for 10 min to collect the erythrocytes. Subsequently, erythrocytes were resuspended in PBS, and then mixed with different samples, including PBS (negative control), pure water (positive control), MSPION3 (*C*_Fe_ = 300 μg/mL), SFN1@MSPION3 (*C*_Fe_ = 300 μg/mL), BQR1@MSPION3 (*C*_Fe_ = 300 μg/mL), or SFN/BQR1@MSPION3 (*C*_Fe_ = 300 μg/mL). The mixtures were incubated for 3.0 h, and then centrifuged (400 g) for 10 min. The absorbance of the supernatants was recorded by a multiplate reader at 540 nm.

**Biosafety evaluation of SFN/BQR1@MSPION3 on tumor-bearing mice:** The healthy Balb/c mice were intravenously injected with 0.10 mL of PBS or SFN/BQR1@MSPION3 at Fe dosage of 5.0 mg/kg or 15 mg/kg. After 24 h, the mice were sacrificed, and blood samples were collected. The blood samples were centrifuged at 3000 rpm for 15 min, and 300 μL of upper serum was taken to determine the blood biochemistry indicators for the major organs and blood routine indicators. Liver: Alanine aminotransferase (ALT) and aspartate transferase (AST), kidney: blood urea nitrogen (BUN) and creatinine (CR), heart: creatine kinase isoenzyme (CK-MB), white blood cell count (WBC), red blood cell count (RBC), platelets count (PLT), average red blood cell volume (MCV), mean hemoglobin volume concentration (MCHC), average hemoglobin volume (MCH), hemoglobin concentration (HGB), and haematocrit (HCT).

For the major organs toxicity detection, the healthy Balb/c mice were intravenously injected with 0.10 mL of SFN/BQR1@MSPION3 at Fe dosage of 5.0 mg/kg. After 72 h, the mice were sacrificed, and major organs (heart, liver, spleen, lung, and kindey) were collected. Then, the H&E staining method was used to observe and analyze the sections of major organs.

**Statistical analysis:** All values are presented as mean ± standard deviations (SD). Measurements were taken from distinct samples. Statistical analysis was carried out using the Student’s t-test for two groups, as well as a one-way analysis of variance for more than two groups. The significance level was fixed as * P < 0.05, ** P < 0.01, *** P < 0.001, or **** P < 0.0001.

**Table S1**. Synthesis conditions of the SPION1-4 by a bubble template method.

| Samples | FeCl_3_·6H_2_O (mg) | NH_4_HCO_3_ (g) | Ethylene Glycol (mL) | Diglycol (mL) | Diglycol/Ethylene Glycol Volume Ratio | Temperature (^o^C) | Reaction Time (h) | Structure |
| --- | --- | --- | --- | --- | --- | --- | --- | --- |
| SPION1 | 405 | 1.185 | 30.0 | 0 | 0 | 200 | 12 | Hollow |
| SPION2 | 405 | 1.185 | 24.3 | 5.7 | 0.23 | 200 | 12 | Solid |
| SPION3 | 405 | 1.185 | 18.6 | 11.4 | 0.61 | 200 | 12 | Immature Mesoporous |
| SPION4 | 405 | 1.185 | 12.9 | 17.1 | 1.33 | 200 | 12 | Amorphous |

**Table S2**. Synthesis conditions of the MSPION1-4.

| Samples | FeCl_3_·6H_2_O (mg) | NH_4_HCO_3_ (g) | ethylene glycol (mL) | Diglycol (mL) | 1^st^ Step of Reaction | 2^nd^ Step of Reaction |
| --- | --- | --- | --- | --- | --- | --- |
| MSPION1 | 405 | 1.185 | 18.6 | 11.4 | 200 ^o^C for 12 h | 250 ^o^C for 0.1 h |
| MSPION2 | 405 | 1.185 | 18.6 | 11.4 | 200 ^o^C for 12 h | 250 ^o^C for 12 h |
| MSPION3 | 405 | 1.185 | 18.6 | 11.4 | 200 ^o^C for 12 h | 250 ^o^C for 72 h |
| MSPION4 | 405 | 1.185 | 18.6 | 11.4 | 200 ^o^C for 12 h | 250 ^o^C for 120 h |

**Table S3**. Synthesis conditions and characterization results of the SFN1-3@MSPION3, BQR1-3@MSPION3, and SFN/BQR1-2@MSPION3.

| Sample  Nomenclature | MSPION3  (mg/mL) *^a^* | SFN  (mg/mL) *^a^* | BQR  (mg/mL) *^a^* | SFN  LC (%) *^b^* | SFN  LE (%) *^c^* | BQR  LC (%) *^b^* | BQR  LE (%) *^c^* | SFN/BQR Molar Ratio *^d^* |
| --- | --- | --- | --- | --- | --- | --- | --- | --- |
| SFN1@MSPION3 | 1.0 | 5.0 | - | 4.9±0.5 | 39.1±3.8 | - | - | - |
| SFN2@MSPION3 | 1.0 | 10.0 | - | 9.8±0.6 | 39.0±2.3 | - | - | - |
| SFN3@MSPION3 | 1.0 | 15.0 | - | 11.5±0.5 | 30.7±1.3 | - | - | - |
| BQR1@MSPION3 | 1.0 | - | 4.15 | - | - | 4.1±0.6 | 39.7±5.4 | - |
| BQR2@MSPION3 | 1.0 | - | 8.30 | - | - | 8.0±0.6 | 38.7±3.0 | - |
| BQR3@MSPION3 | 1.0 | - | 12.45 | - | - | 10.1±0.9 | 32.5±2.8 | - |
| SFN/BQR1@MSPION3 | 1.0 | 5.0 | 4.15 | 5.1±0.4 | 40.9±3.0 | 4.1±0.2 | 39.1±2.0 | 0.99 |
| SFN/BQR2@MSPION3 | 1.0 | 10.0 | 8.30 | 5.5±0.4 | 22.0±1.7 | 4.5±0.4 | 21.9±1.9 | 0.98 |

*^a^* Feeding volume of MSPION, SFN, and BQR is 20, 0.5, and 0.5 mL, respectively.

*^b^* SFN or BQR loading content: calculated from the mass percentage of the loaded SFN or BQR to the HMON nanoparticles.

*^c^* SFN or BQR loading efficiency: calculated from the mass percentage of the loaded SFN or BQR to the feeding SFN or BQR.

*^d^* Calculated from the molar ratio of the loaded SFN to the loaded BQR.


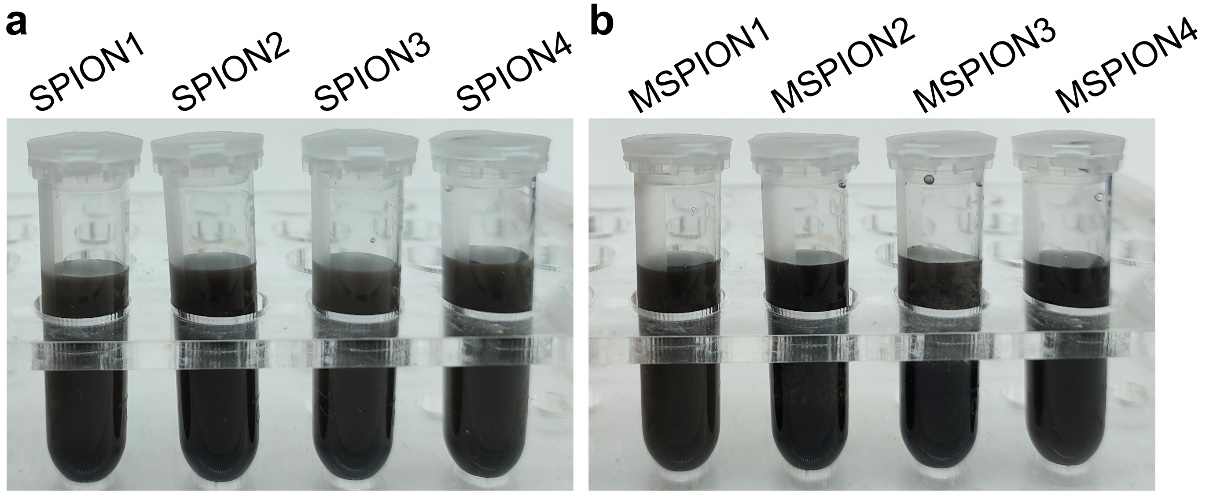


**Fig. S1.** (A, B): Photographs of the purified SPION1-4 (A), and MSPION1-4 (B) dispersed in ultrapure water.


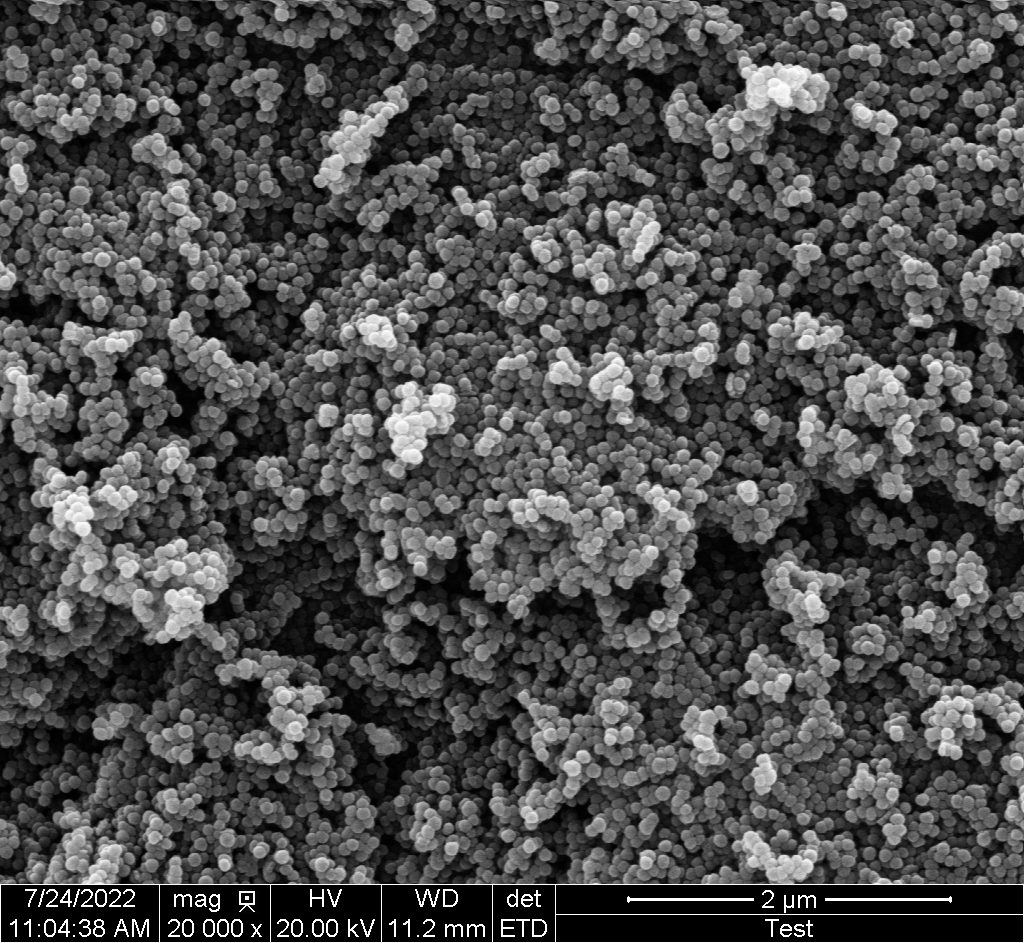


**Fig. S2.** The SEM image of MSPION3 in a wide field of view.


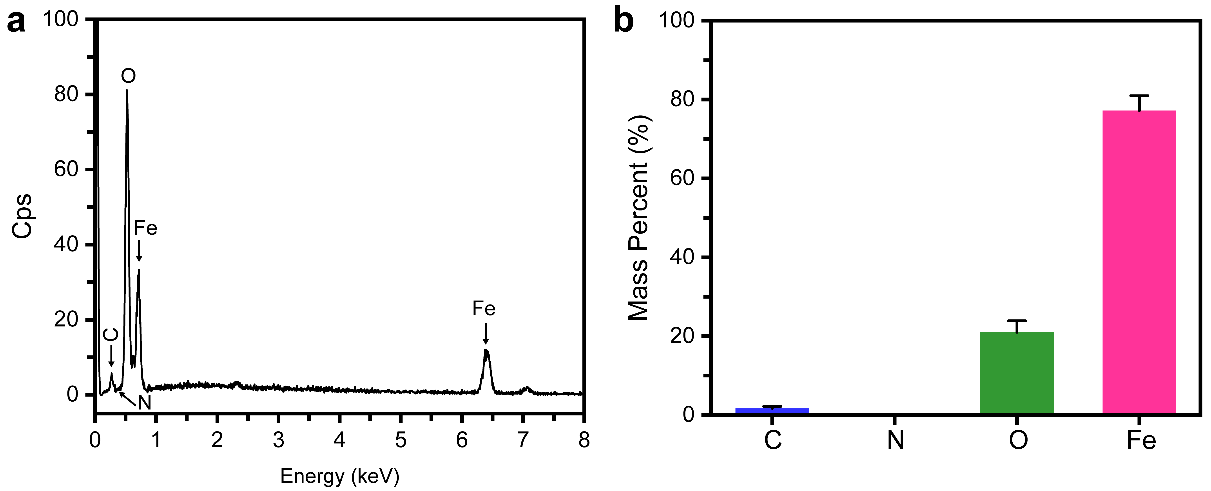


**Fig. S3.** Energy dispersive X-ray spectroscopy (EDS) spectrum of MSPION3 with the peaks of C, N, O, Fe, and Fe (A) and the corresponding mass percent (B).


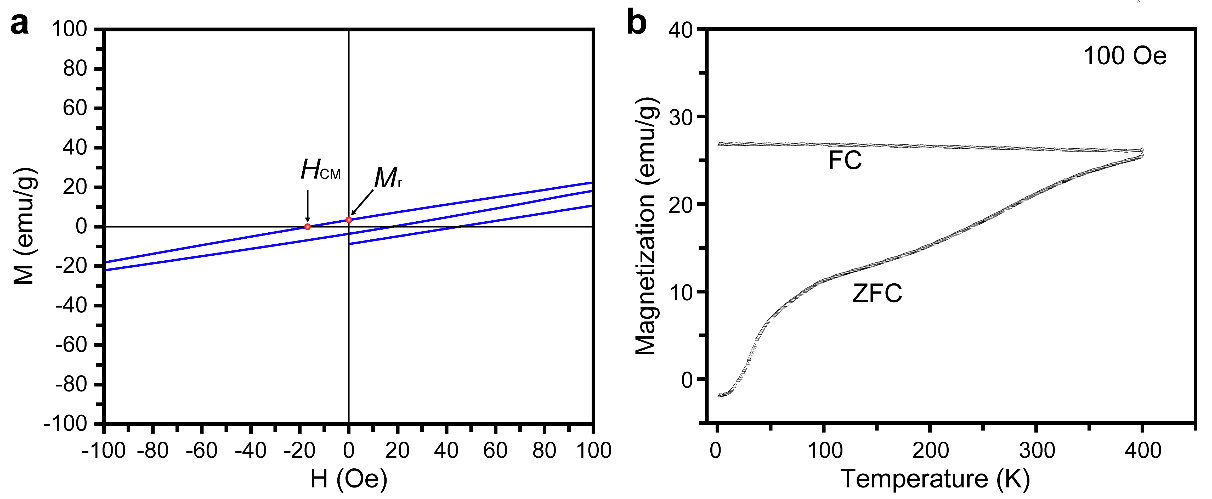


**Fig. S4.** (A): Magnetization *vs.* Magnetic Field M-H plot of pure MSPION3 measured at 300 K for the enlarged hysteresis loop. (B): ZFC (Zero Field Cooled) and FC (Field Cooled) temperature dependent magnetization M-T measurement of pure MSPION3 at applied magnetic field of 100 Oe.


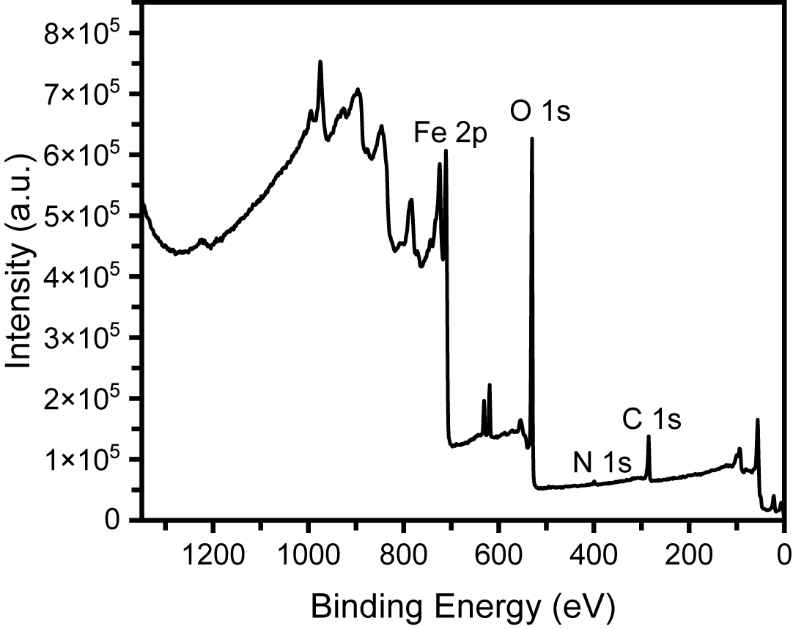


**Fig. S5**. The X-ray photoelectron spectroscopy (XPS) spectrum of MSPION3 with the peaks of Fe2p, O1s, N1s, and C1s.


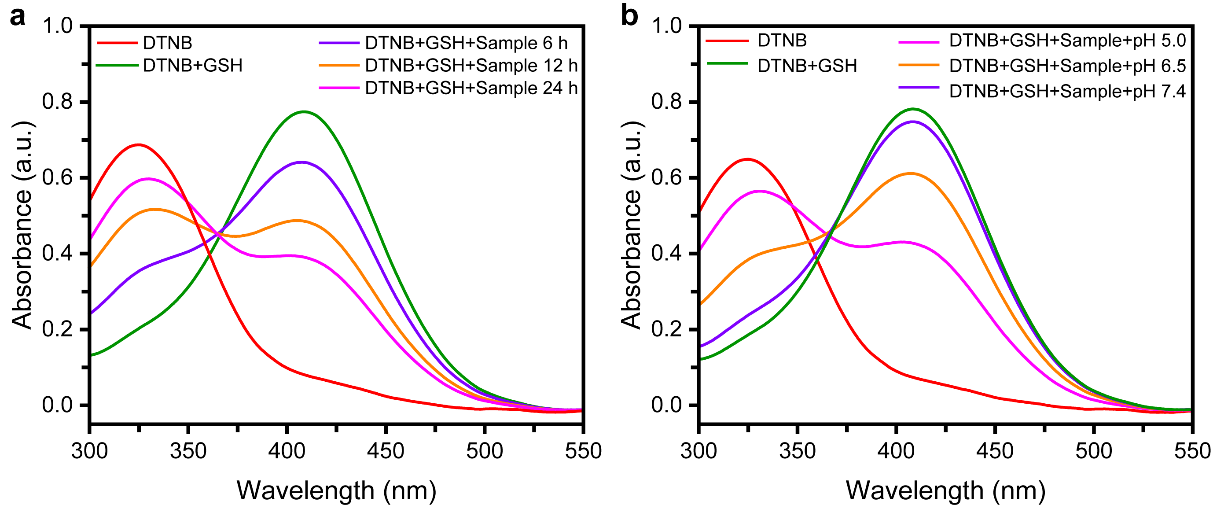


**Fig. S6.** (A, B): UV-vis spectra of the DTNB solutions incubated with or without GSH and/or MSPION3 for 6, 12, or 24 h at pH 5.0 (A), or for 24 h at pH 5.0, 6.5, or 7.4 (B) to testify the GSH consumption capacities.


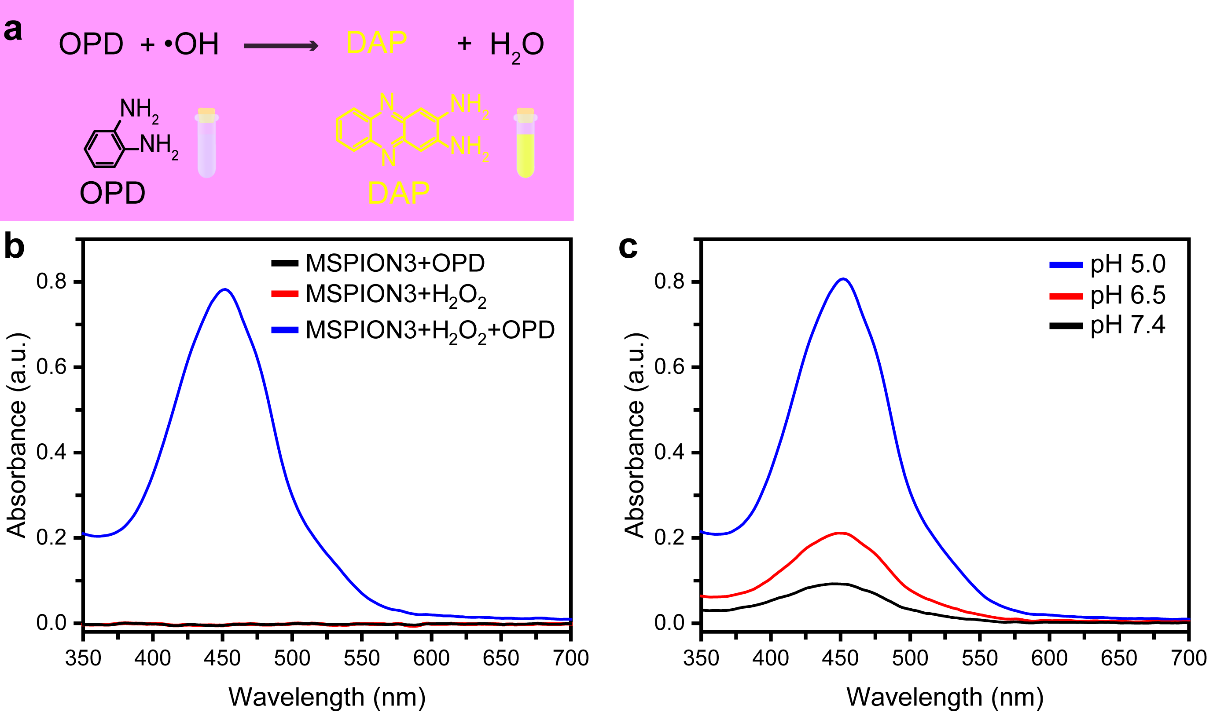


**Fig. S7.** (A): Schematic illustration of the OPD colorimetric method for the measurement of •OH generation. (B): UV-vis spectra of the MSPION3 solutions at pH 5.0 with OPD and/or H_2_O_2_ to testify the •OH generation. (C): UV-vis spectra of the MSPION3 solutions with TMB and H_2_O_2_ to testify the •OH generation at different pH values (pH 5.0, 6.5, or 7.4) for 70 h.


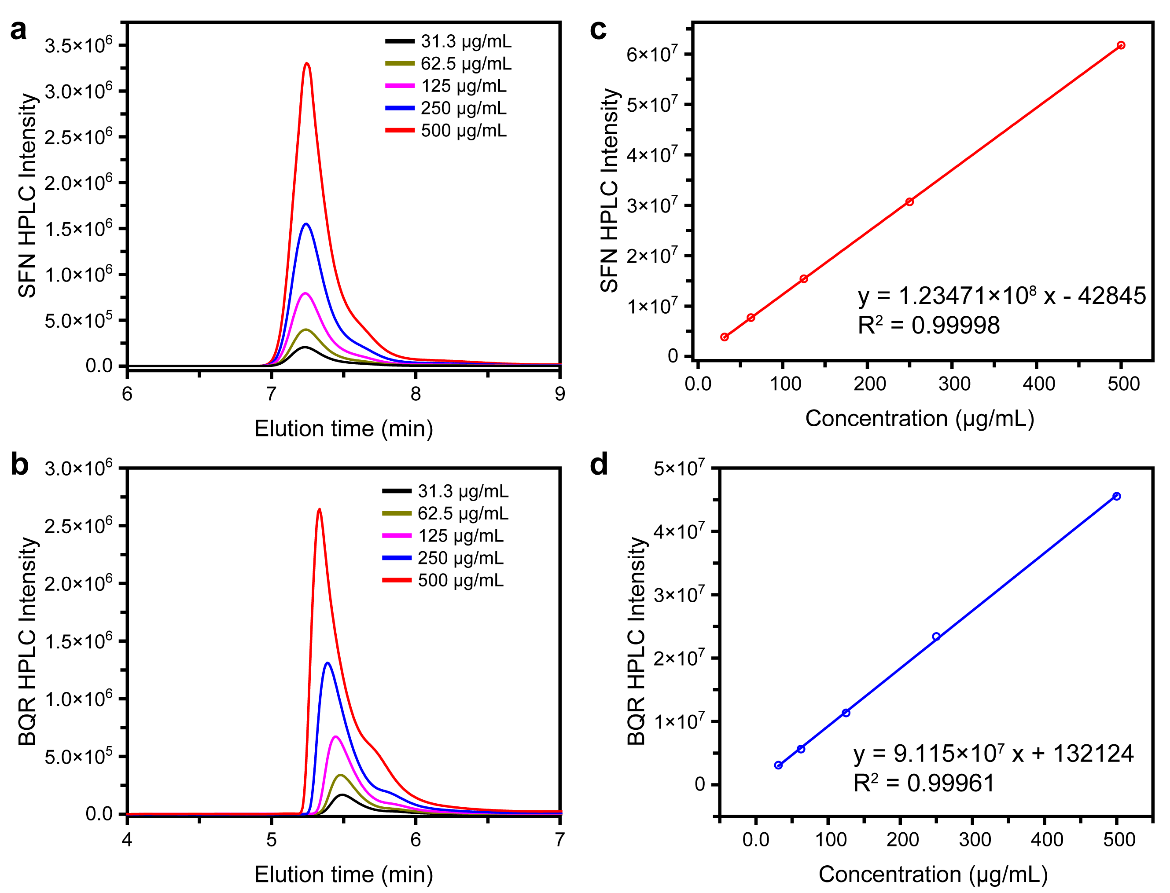


**Fig. S8**. (A, B): Chromatogram of SFN (A), or BQR (B) solutions with various concentrations (31.3-500 µg/mL) measured by high performance liquid chromatography (HPLC) at 265, and 254 nm, respectively. (C, D): Standard curve of SFN (C), or BQR (D) constructed from the maximum HPLC intensity as a function of the SFN or BQR concentrations.


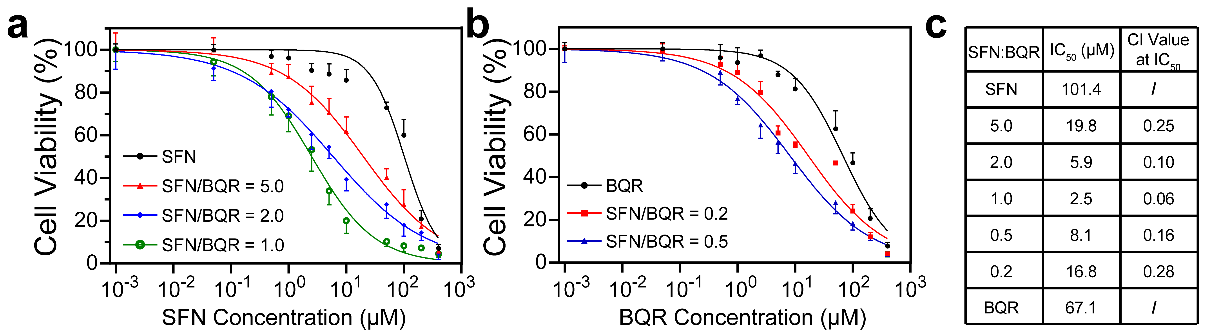


**Fig. S9.** (A, B): 4T1 cell viability treated by SFN or SFN plus BQR with the molar ratio of 5.0, 2.0, or 1.0 (A), or treated by BQR or SFN plus BQR with the molar ratio of 0.2, or 0.5 (B) for 24 h. (C): The IC_50_ and corresponding CI values of SFN, BQR, and SFN/BQR combination calculated from Fig. A and B.


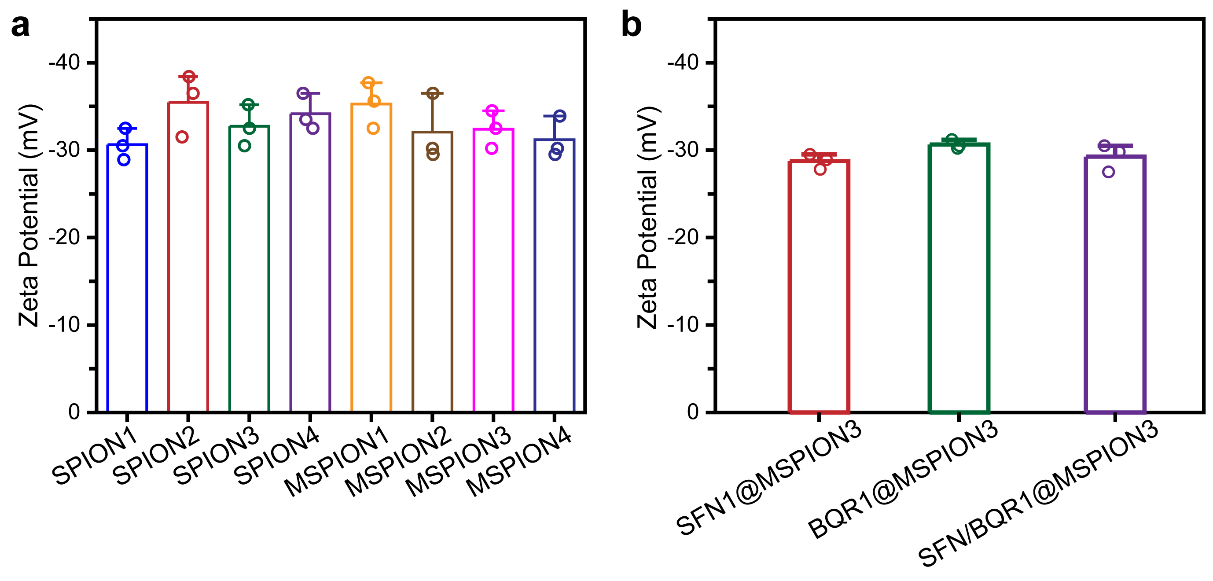


**Fig. S10.** (A, B): Zeta potentials of SPION1-4, MSPION1-4 (A), and SFN1@MSPION3, BQR1@MSPION3, and SFN/BQR1@MSPION3 (B) in ultrapure water.


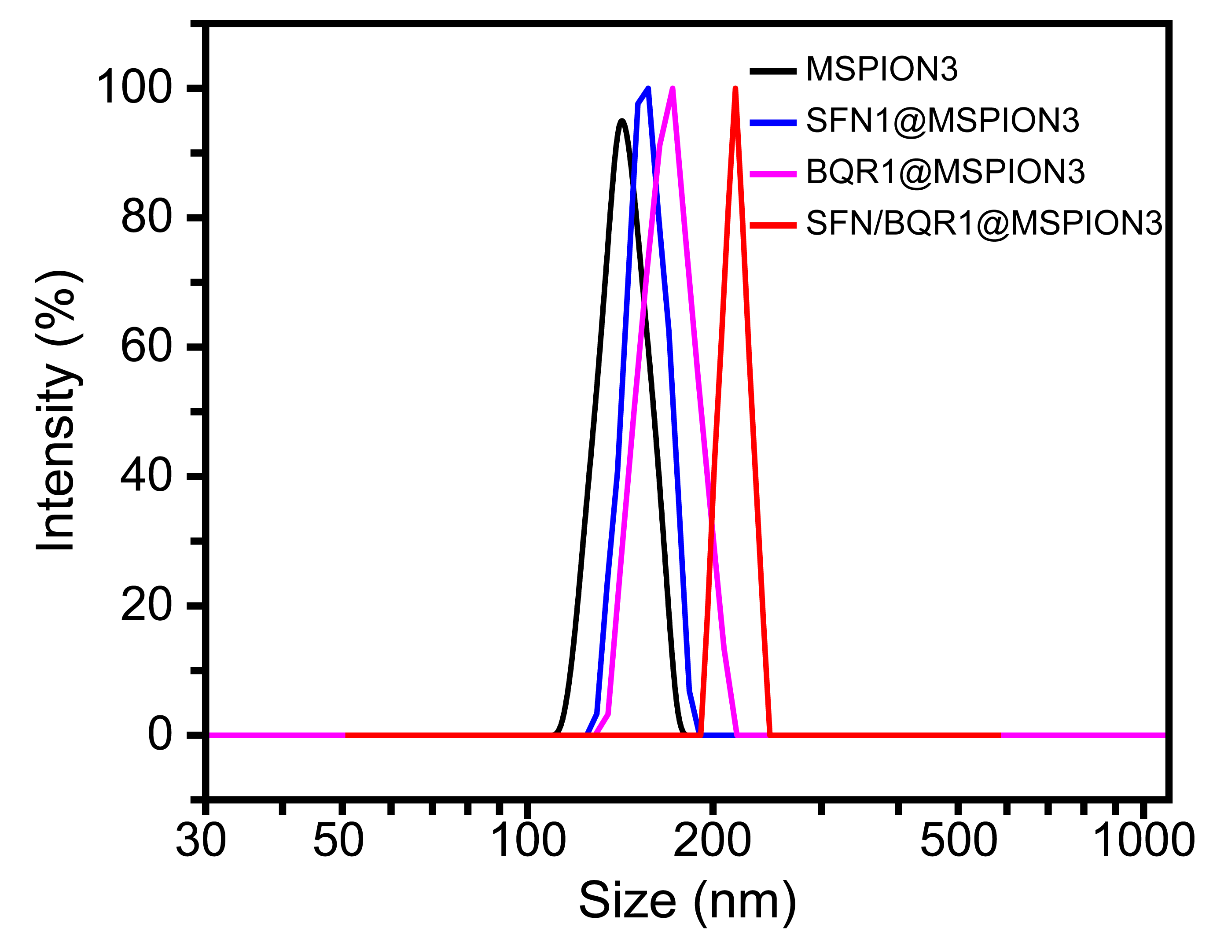


**Fig. S11.** Size distributions of MSPION3, SFN1@MSPION3, BQR1@MSPION3 and SFN/BQR1@MSPION3 measured by dynamic light scattering (DLS) in ultrapure water.


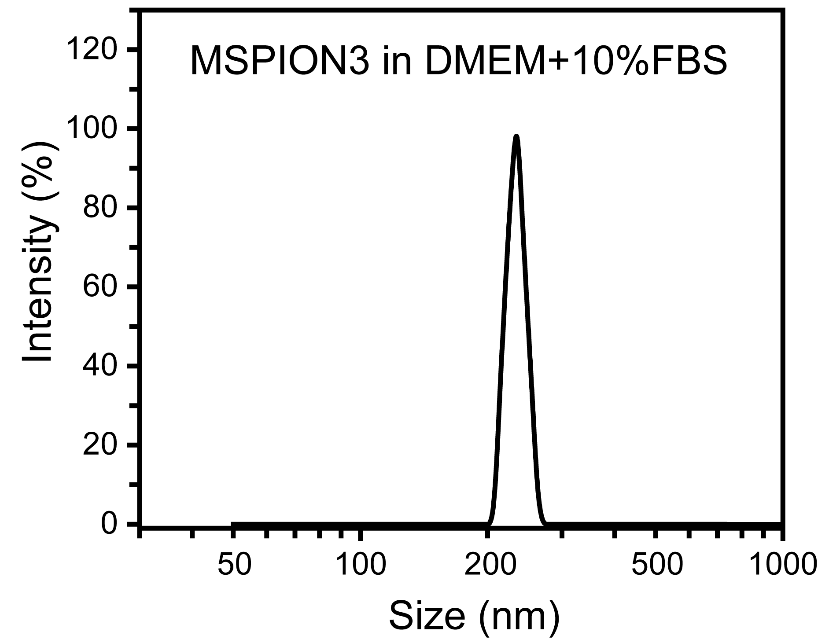


**Fig. S12.** Size distributions of MSPION3 measured by dynamic light scattering (DLS) in DMEM medium (containing 10% FBS).


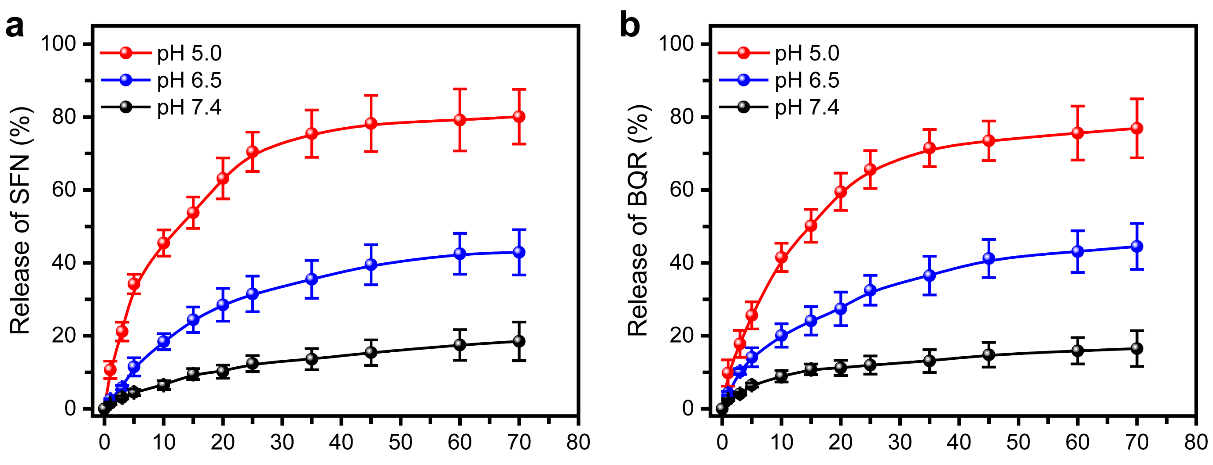


**Fig. S13.** (A, B): Release curves of SFN (A), or BQR (B) in PBS with pH 5.0, 6.5, or 7.4 at 37 ^o^C from SFN/BQR1@MSPION3.


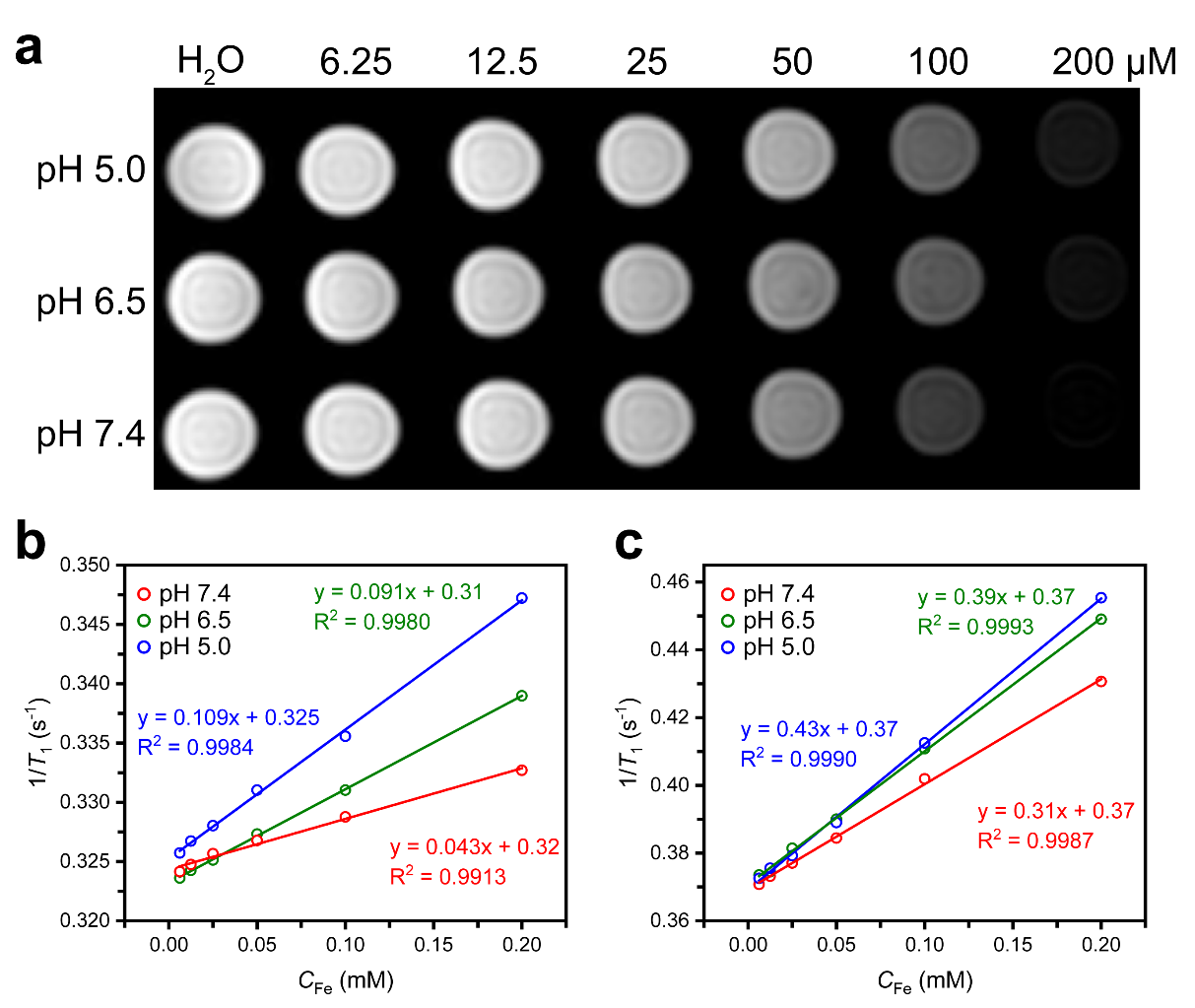


**Fig. S14.** *T*_2_-weighted MR image of SFN1/BQR1@MSPION3 solutions with various *C*_Fe_ (0 ~ 200 μM) incubated at different pH values (5.0, 6.5, or 7.4) for 24 h observed by a 3.0 T clinical MRI system (TE = 400 ms, TR = 7.30 ms). (B, C): *T*_1_ relaxation rate (1/*T*_1_) plotted as a function of *C*_Fe_ for SFN/BQR1@MSPION3 in magnetic field of 7.0 T (B) and 3.0 T (C). For *T*_1_ relaxation rates in magnetic field 7.0 T: TR = 75.8 ms, TE = 6.0 ms. For *T*_1_ relaxation rates in magnetic field 3.0 T: TR = 200 ms, TE = 8.2 ms.


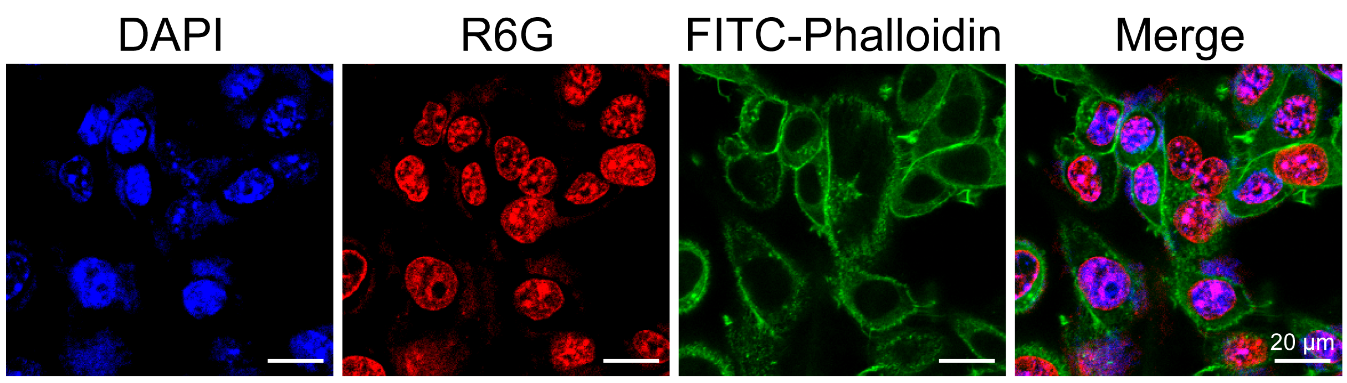


**Fig. S15.** CLSM images of 4T1 cells showing intracellular uptake of pure R6G at 4.0 h. Green fluorescence: FITC-Phalloidin for cytoskeleton. Red fluorescence: R6G for pure R6G. Blue fluorescence: DAPI for nuclei.


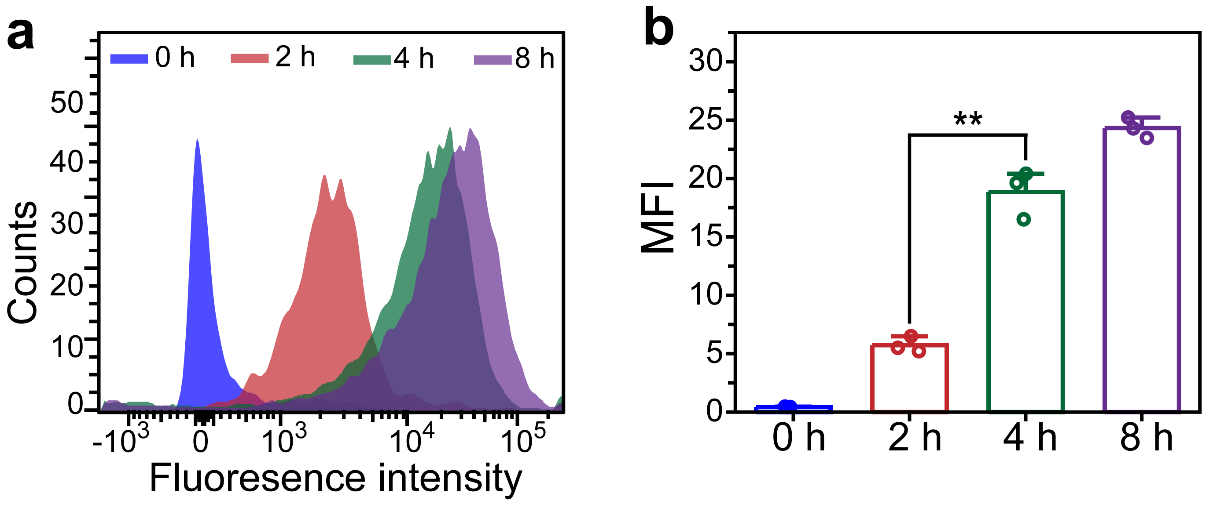


**Fig. S16**. (A, B): Fluorescence distributions (A) and the corresponding quantitative analysis (B) of 4T1 cells treated with R6G@SFN/BQR1@MSPION3 for 0, 2.0, 4.0, and 8.0 h measured by flow cytometry.


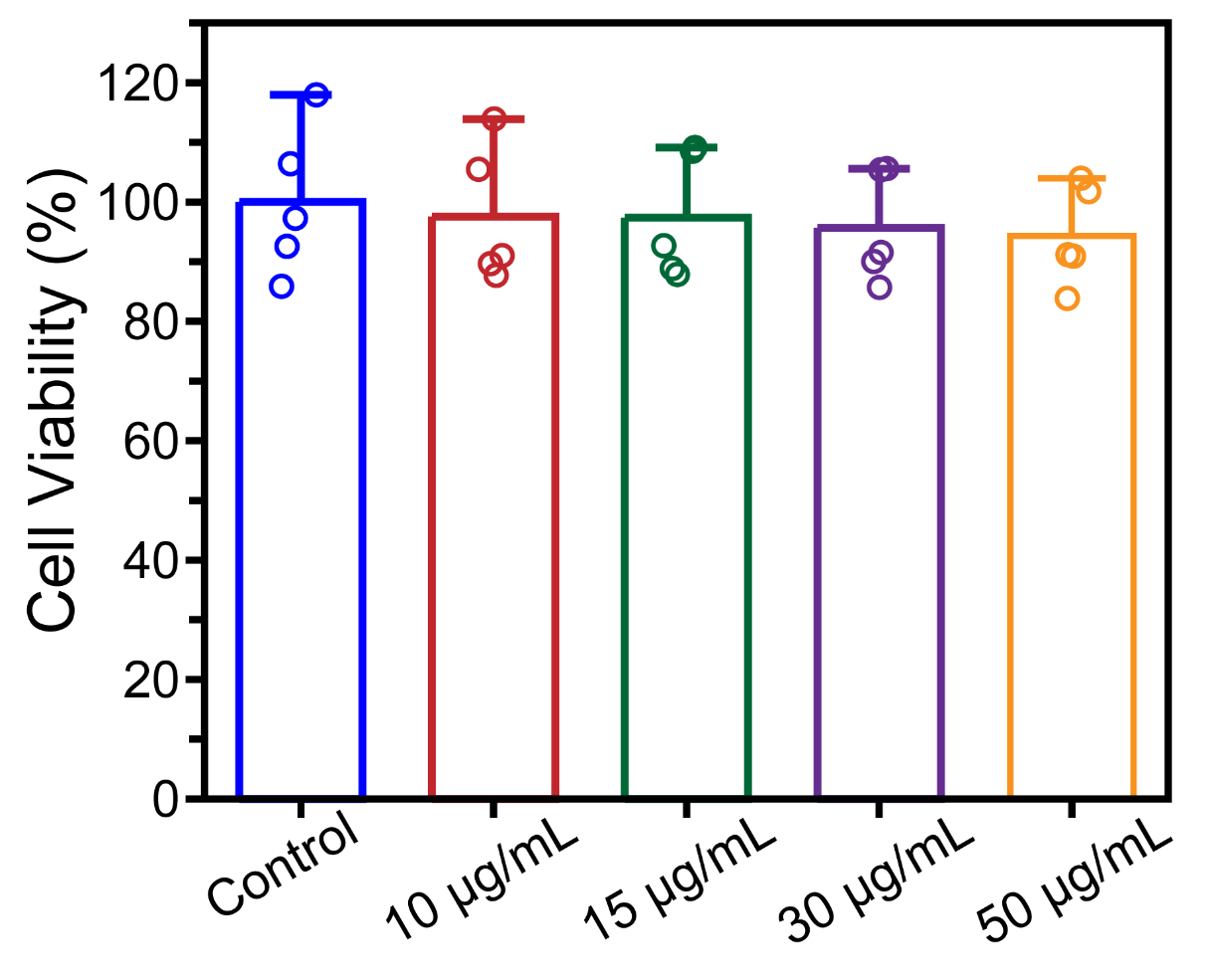


**Fig. S17.** 4T1 cell viabilities incubated with PBS (control), or MSPION3 (*C*_Fe_ = 10, 15, 30, or 50 μg/mL) for 24 h.


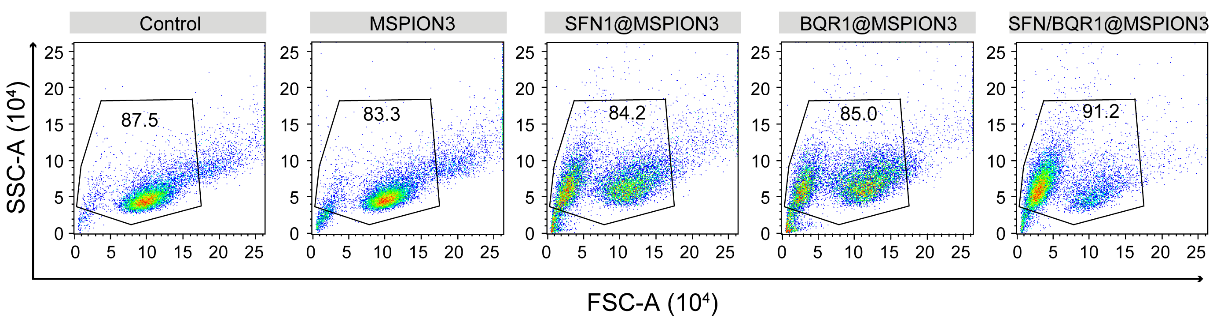


**Fig. S18.** The detailed gating conditions using SSC/FSC strategy to select intact cells and exclude debris and cell aggregates.


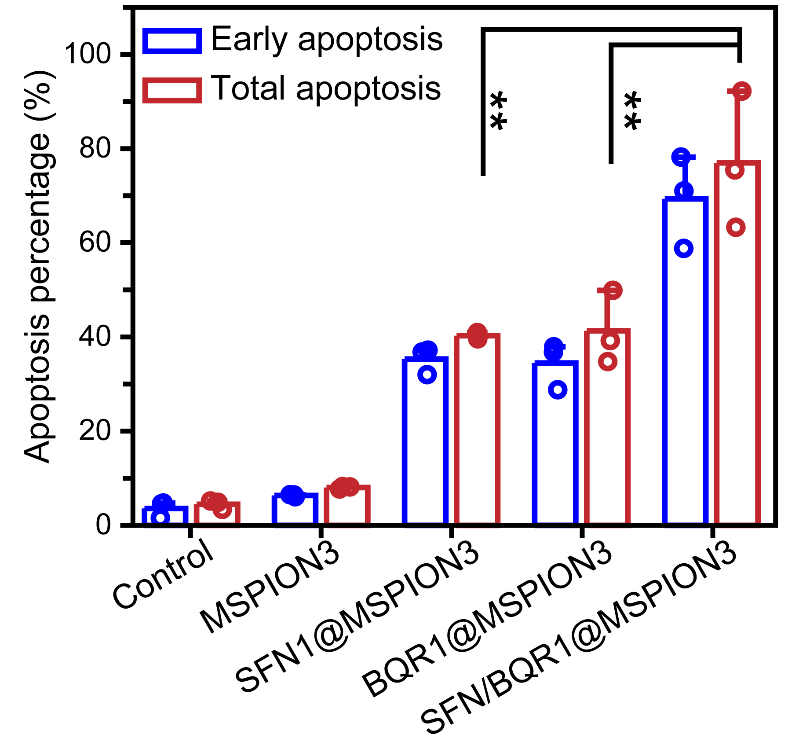


**Fig. S19.** The early and total apoptosis statistics of 4T1 cells after incubation with PBS (control), MSPION3, SFN1@MSPION3, BQR1@MSPION3, or SFN/BQR1@MSPION3 ([Fe] equivalent to 10 μg/mL) for 24 h measured by flow cytometry. ***P* < 0.01.


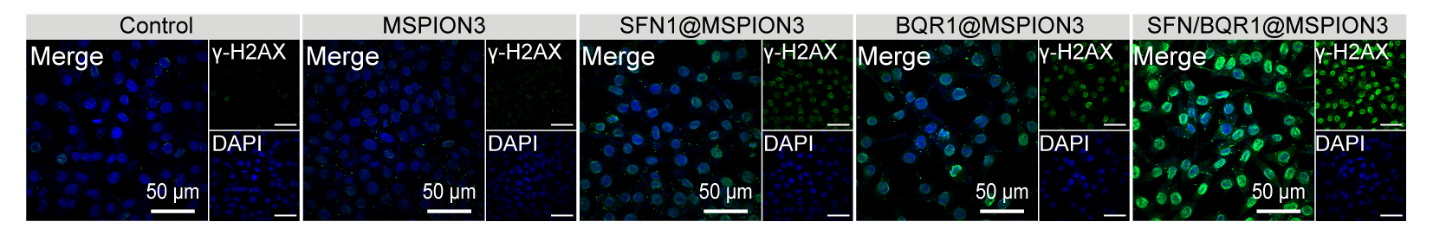


**Fig. S20.** CLSM images of 4T1 cells treated with PBS (control), MSPION3, SFN1@MSPION3, BQR1@MSPION3, or SFN/BQR1@MSPION3 (*C*_Fe_ = 10 μg/mL) for 24 h, showing the degree of DNA damage. Green fluorescence: γ-H2AX for damaged DNA. Blue fluorescence: DAPI for nuclei.


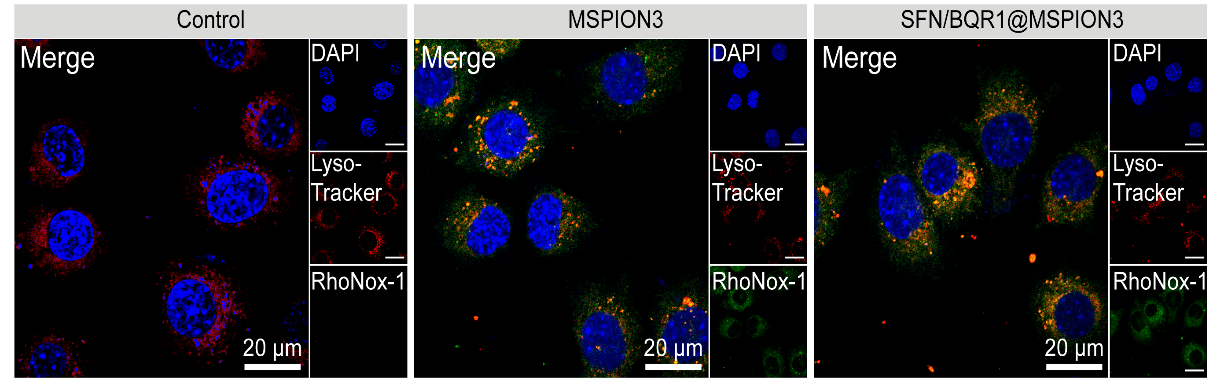


**Fig. S21.** CLSM images of 4T1 cells treated without or with MSPION3 or SFN/BQR1@MSPION3 for 6.0 h, showing intracellular Fe^2+^ outside lysosomes. Green fluorescence: RhoNox-1 for Fe^2+^. Red fluorescence: Lyso-Tracker for lysosomes. Blue fluorescence: DAPI for nuclei.


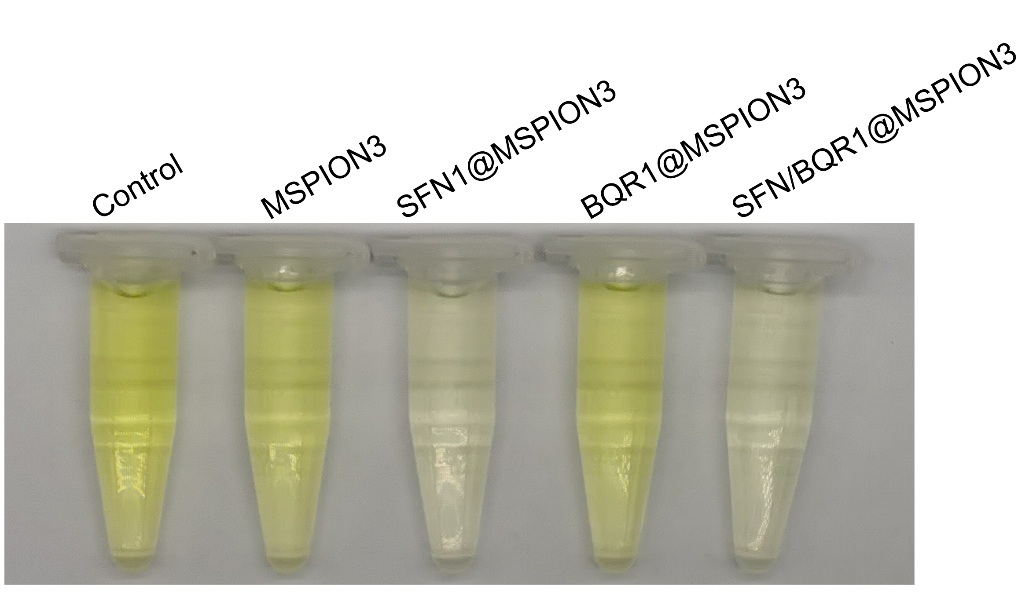


**Fig. S22.** The photo of 4T1 cell solutions treated with PBS (control), or the PBS containing nanoparticles (MSPION3, SFN1@MSPION3, BQR1@MSPION3, and SFN/BQR1@MSPION3) for 24 h using 5,5’-Dithiobis (2-nitrobenzoic acid) (DTNB) method.


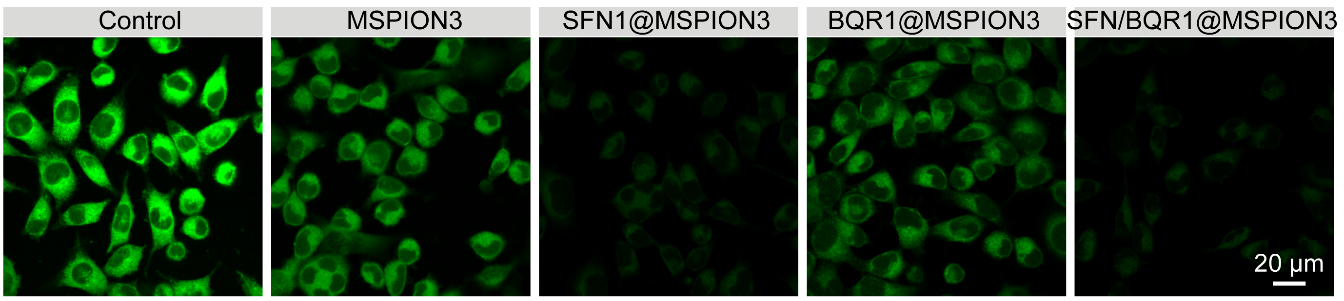


**Fig. S23.** CLSM images of 4T1 cells after treatment with PBS, MSPION3, SFN1@MSPION3, BQR1@MSPION3, or SFN1/BQR1@MSPION3 for 24 h, and staining with Thiol Tracker Violet, showing intracellular GSH with green fluorescence.


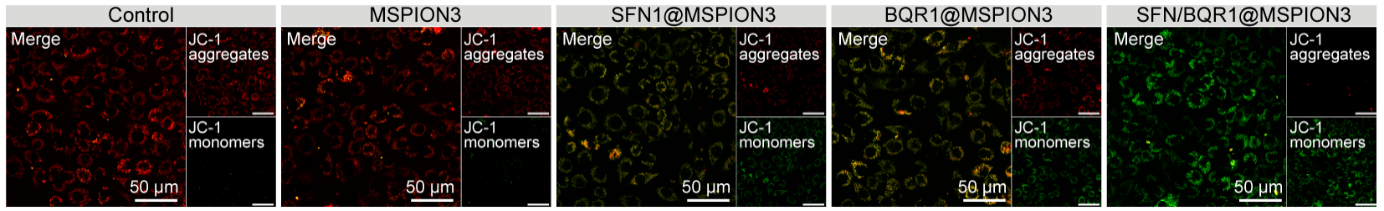


**Fig. S24.** CLSM images of the 4T1 cells after treatment with PBS (control), MSPION3, SFN1@MSPION3, BQR1@MSPION3, or SFN/BQR1@MSPION3 for 24 h, showing the changes of the mitochondrial membrane potential of 4T1 cells. Red fluorescence: JC-1 aggregates for healthy mitochondrial. Green fluorescence: JC-1 monomers for damaged mitochondrial.


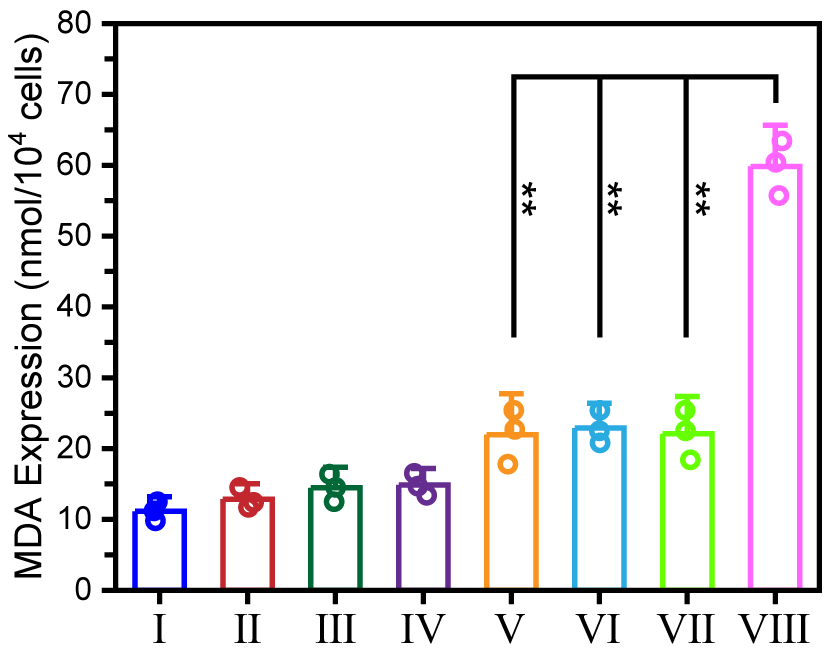


**Fig. S25.** Relative MDA level of 4T1 cells after treatment with PBS (I), MSPION3 (II), SFN (III), BQR (IV), SFN + BQR (V), SFN1@MSPION3 (Ⅵ), BQR1@MSPION3 (Ⅶ), or SFN/BQR1@MSPION3 (Ⅷ) for 24 h.


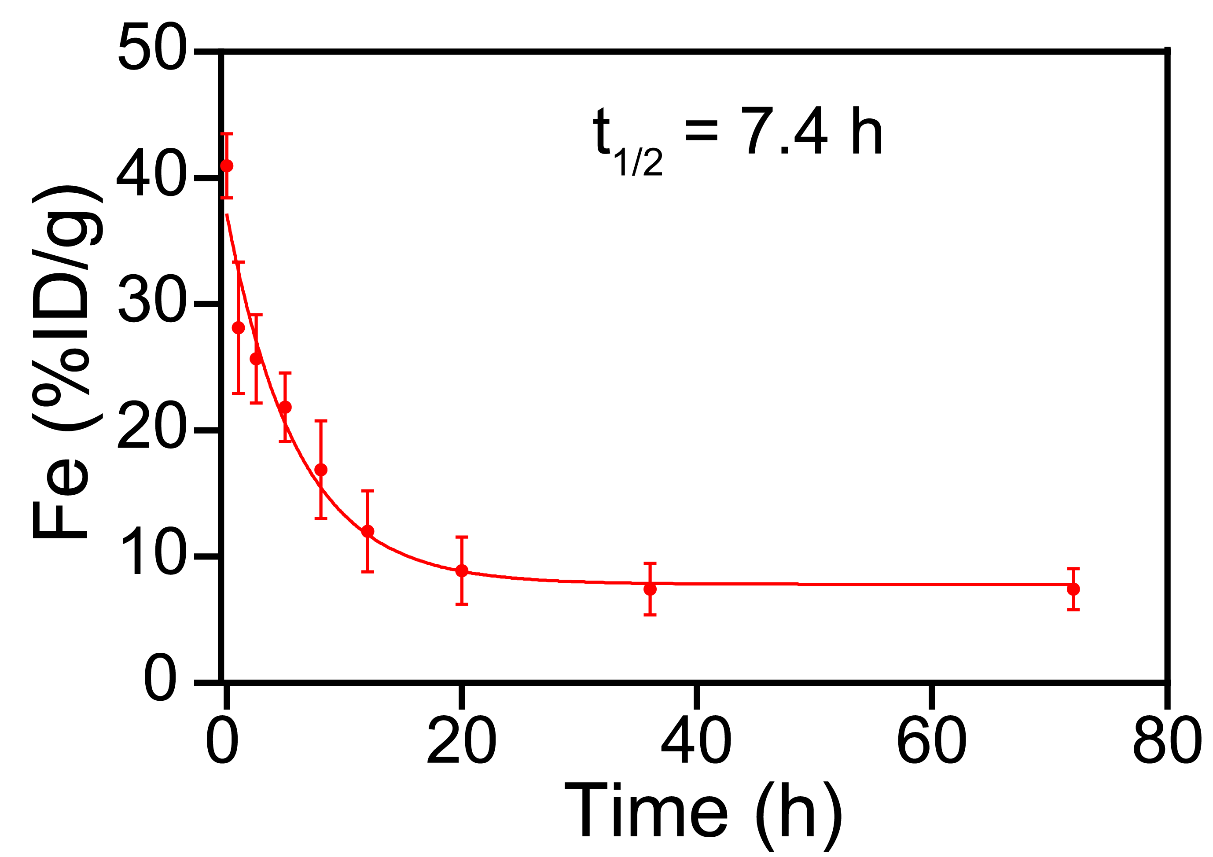


**Fig. S26.** Blood clearance profile of SFN/BQR1@MSPION3 (C*_Fe_* = 5.0 mg/kg) in mice after i.v. injection.


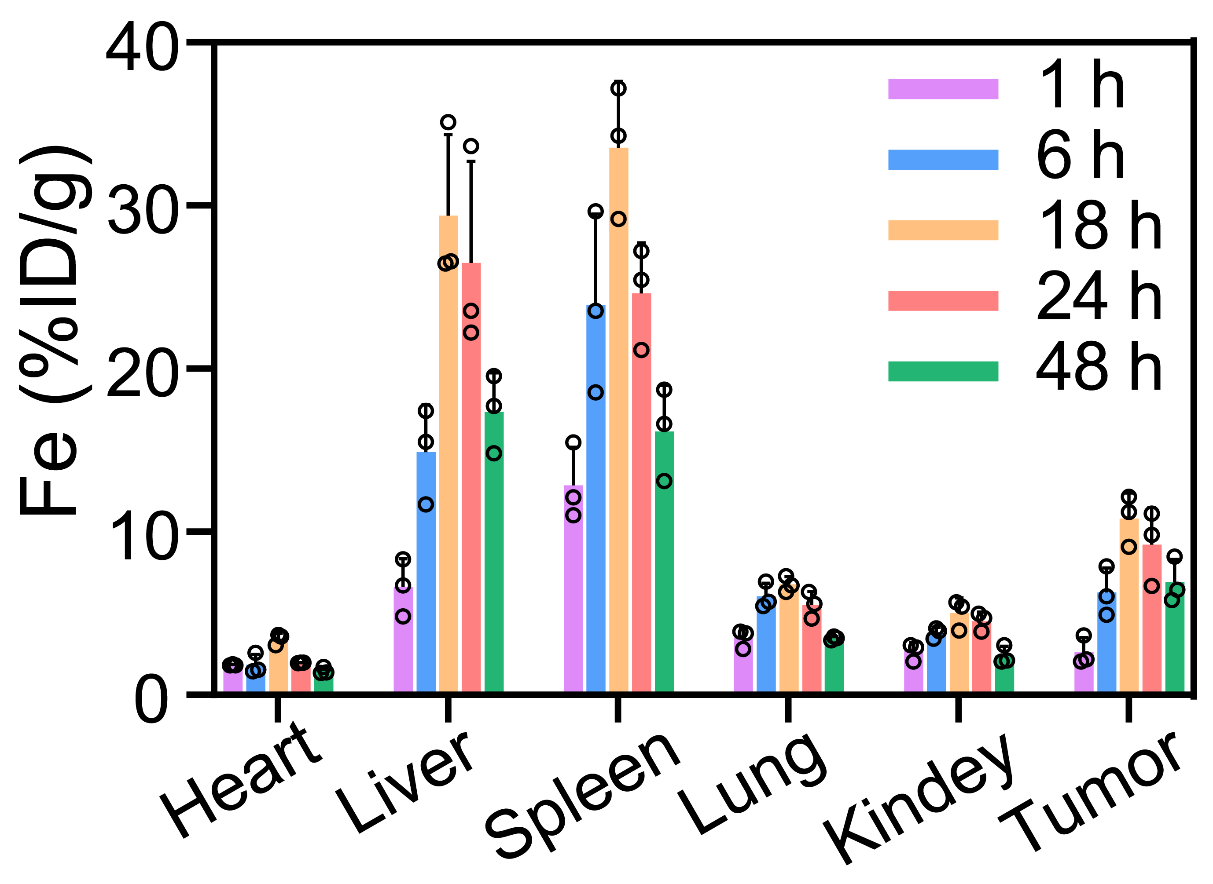


**Fig. S27.** Biodistribution of Fe at 1.0, 6.0, 18, 24, and 48 h post-injection (*i.v.*) of SFN/BQR1@MSPION3 (C*_Fe_* = 5.0 mg/kg).


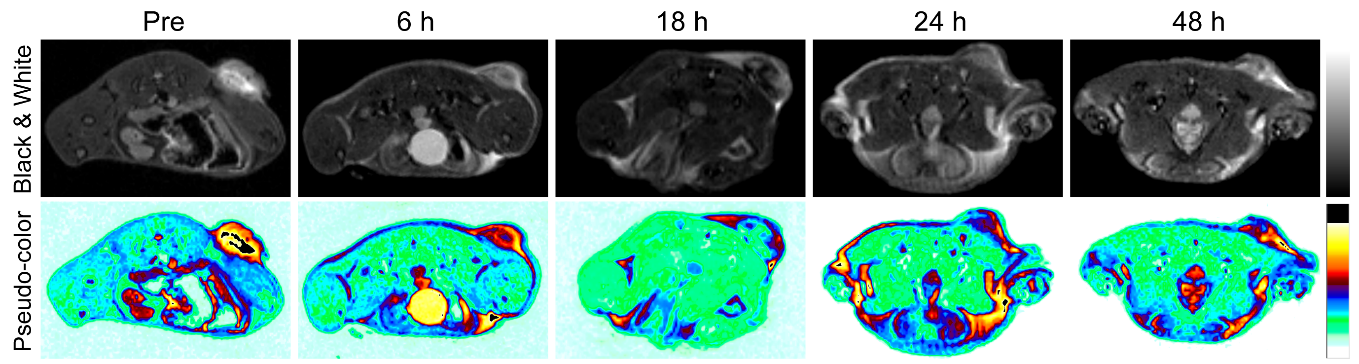


**Fig. S28.** Axial orientation of *T*_2_-weighted MR images for 4T1 tumor-bearing mice pre- and post-injection of SFN/BQR1@MSPION3 (Fe dosage is 5.0 mg/kg) at different time intervals (6, 18, 24, and 48 h).


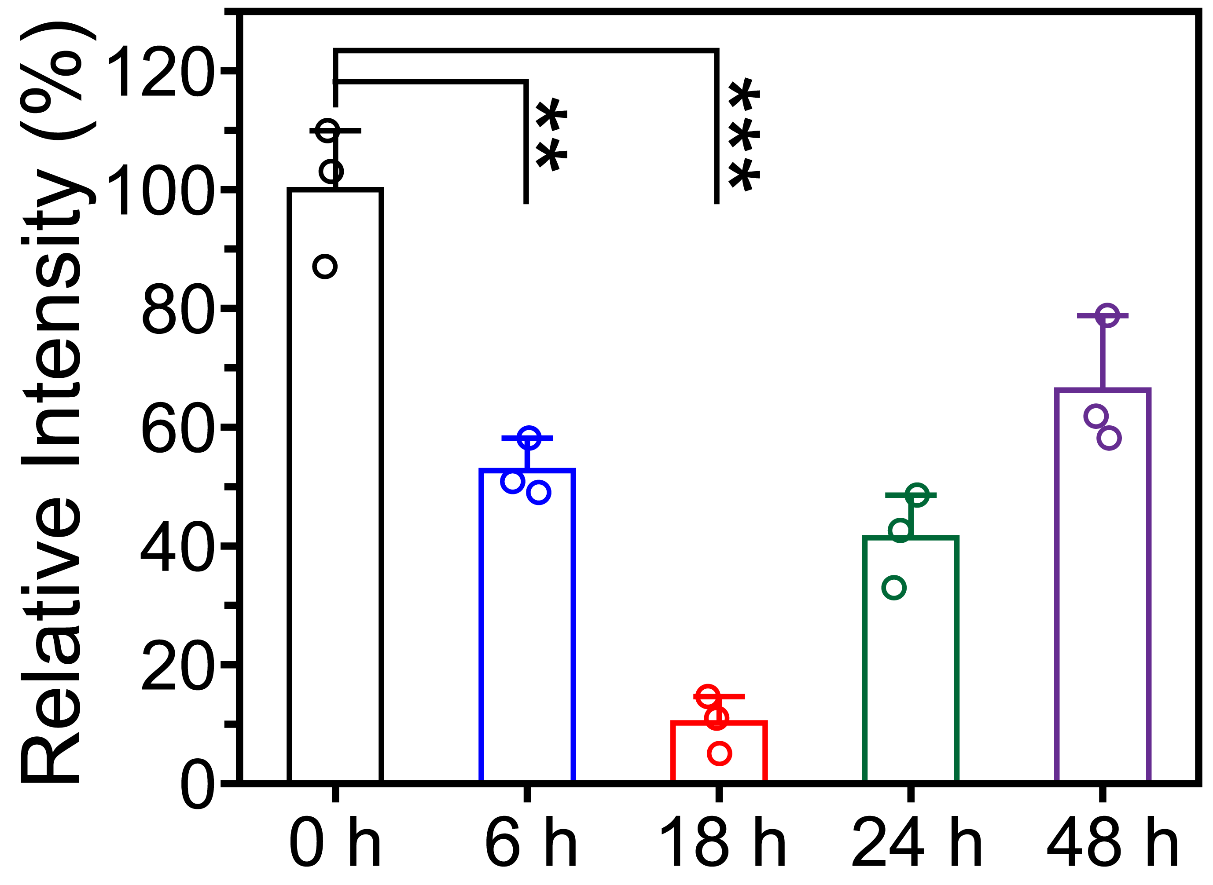


**Fig. S29.** The corresponding quantitative analysis of the tumor MR images in Fig. S26, using relative intensity.


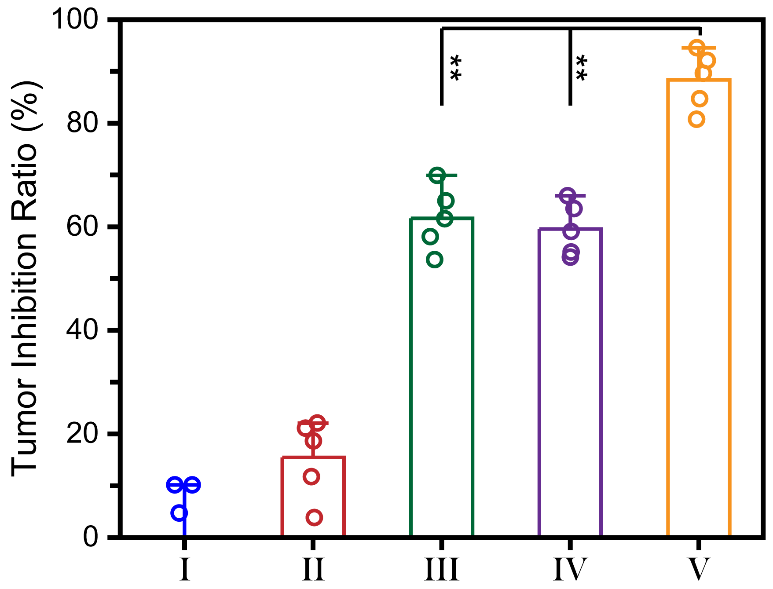


**Fig. S30.** The tumor inhibition ratio for the 4T1 tumor-bearing mice after treatments with PBS (I), MSPION3 (II), SFN1@MSPION3 (III), BQR1@MSPION3 (IV), or SFN/BQR1@MSPION3 (V) for 14 days. ***P* < 0.01.


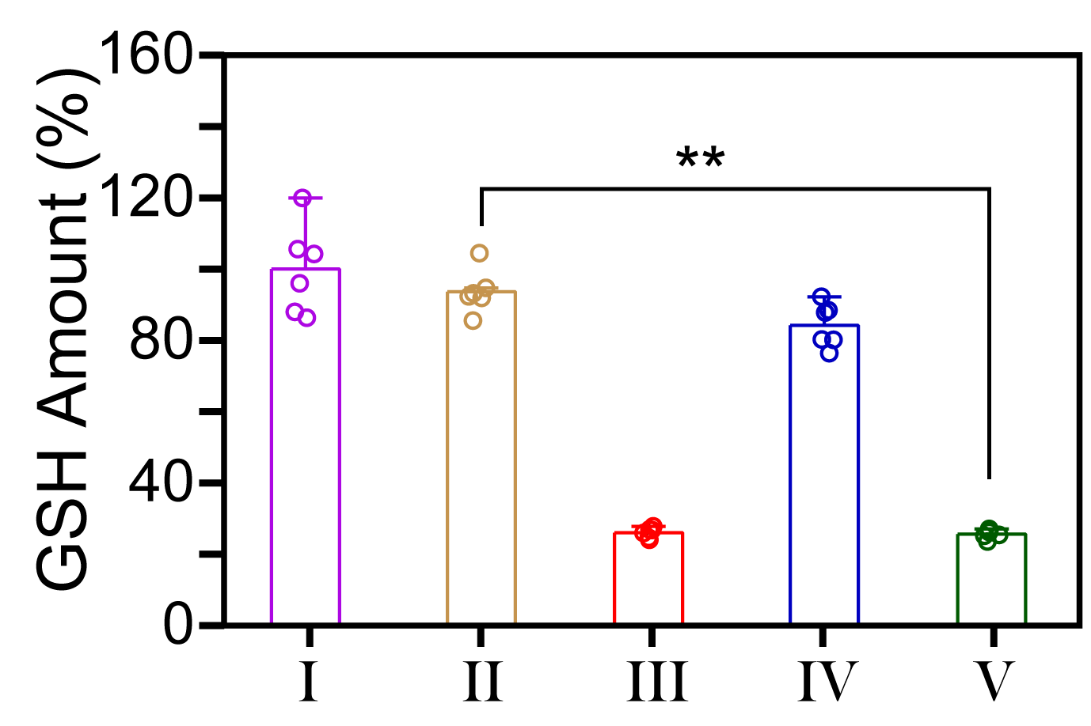


**Fig. S31.** Evaluations of the GSH level in tumors on day 14 after treatments with PBS (Ⅰ), MSPION3 (Ⅱ), SFN1@MSPION3 (Ⅲ), BQR1@MSPION3 (Ⅳ), or SFN/BQR1@MSPION3 (Ⅴ) by intravenous injection on day 0 and 7. Fe dosage = 5.0 mg/kg. ***P* < 0.01.


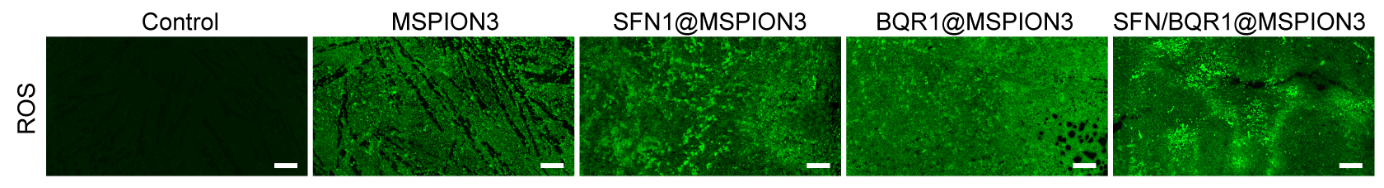


**Fig. S32.** Representative fluorescence microscopic pictures of sectioned tumor tissues from 4T1 tumor-bearing mice on day 14 post-treatment with PBS (control), MSPION3, SFN1@MSPION3, BQR1@MSPION3, or SFN/BQR1@MSPION3 after ROS staining with DCFH-DA.


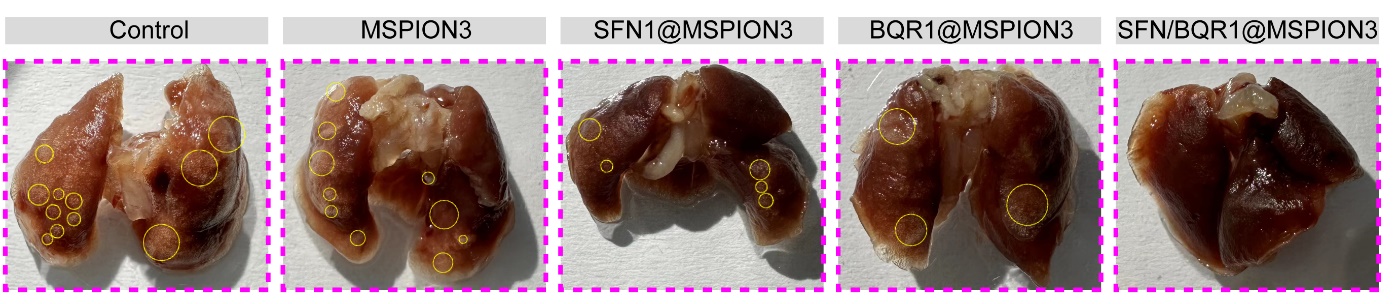


**Fig. S33.** Representative lung photographs in 4T1 tumor-bearing mice on day 14 post-treatment with PBS (control), MSPION3, SFN1@MSPION3, BQR1@MSPION3, or SFN/BQR1@MSPION3.


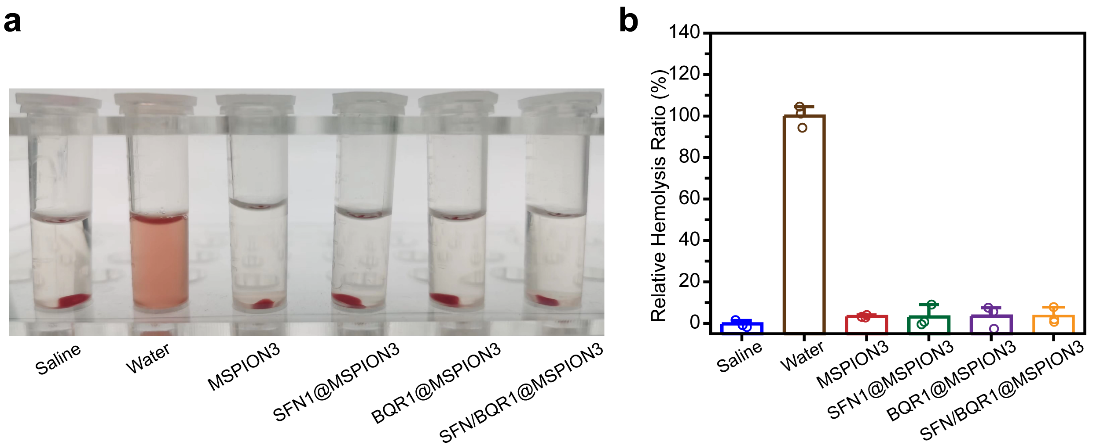


**Fig. S34.** (A, B): Hemolysis (A), and qualification of the hemolysis rates (B) of Saline, MSPION3, SFN1@MSPION3, BQR1@MSPION3, and SFN/BQR1@MSPION3 with the Fe concentration of 300 μg/mL compared with ultrapure water.


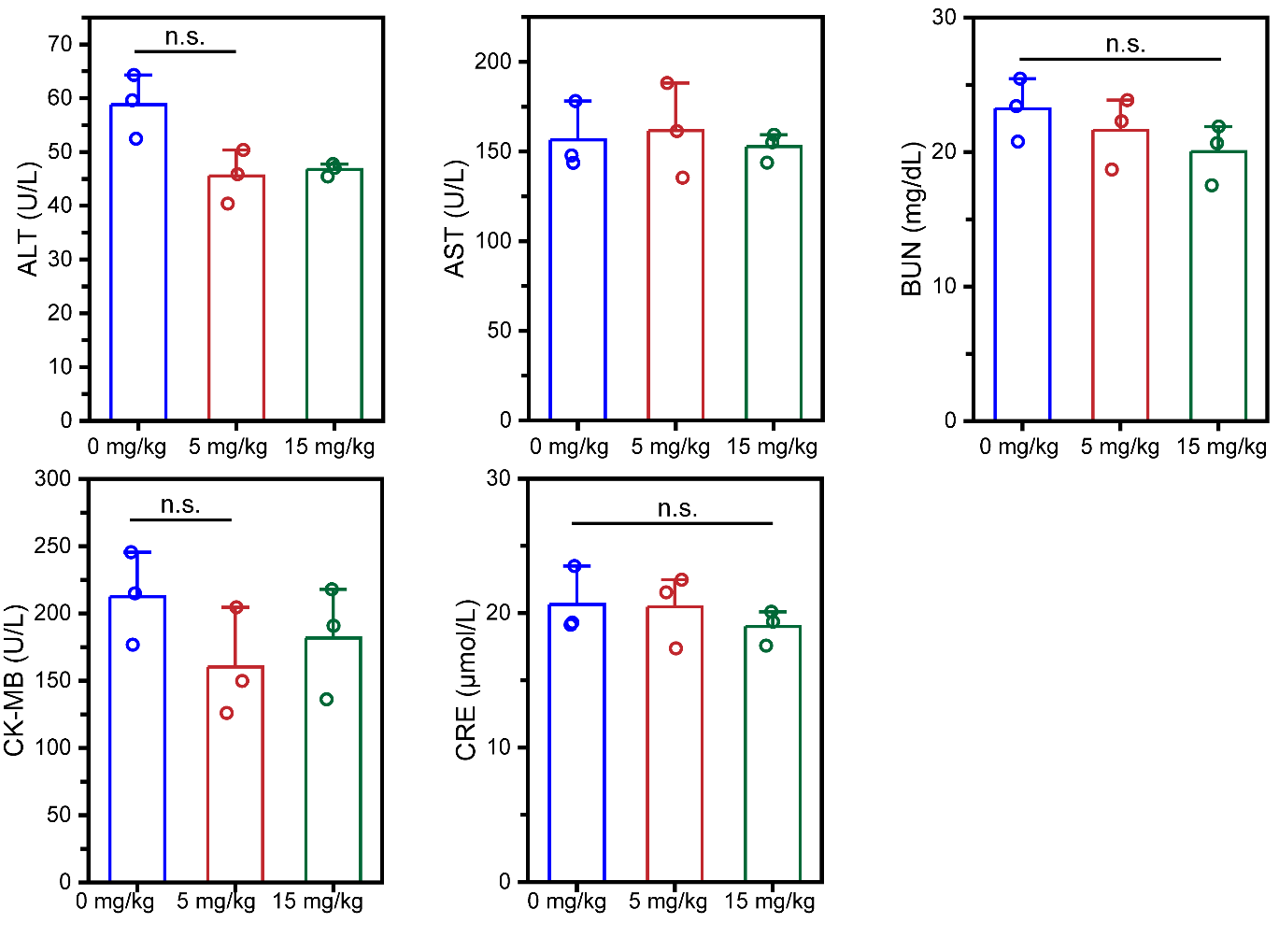


**Fig. S35.** Blood biochemistry analysis of healthy mice after treatments with saline (control), or SFN/BQR1@MSPION3 (*C*_Fe_ = 5 or 15 mg/kg).


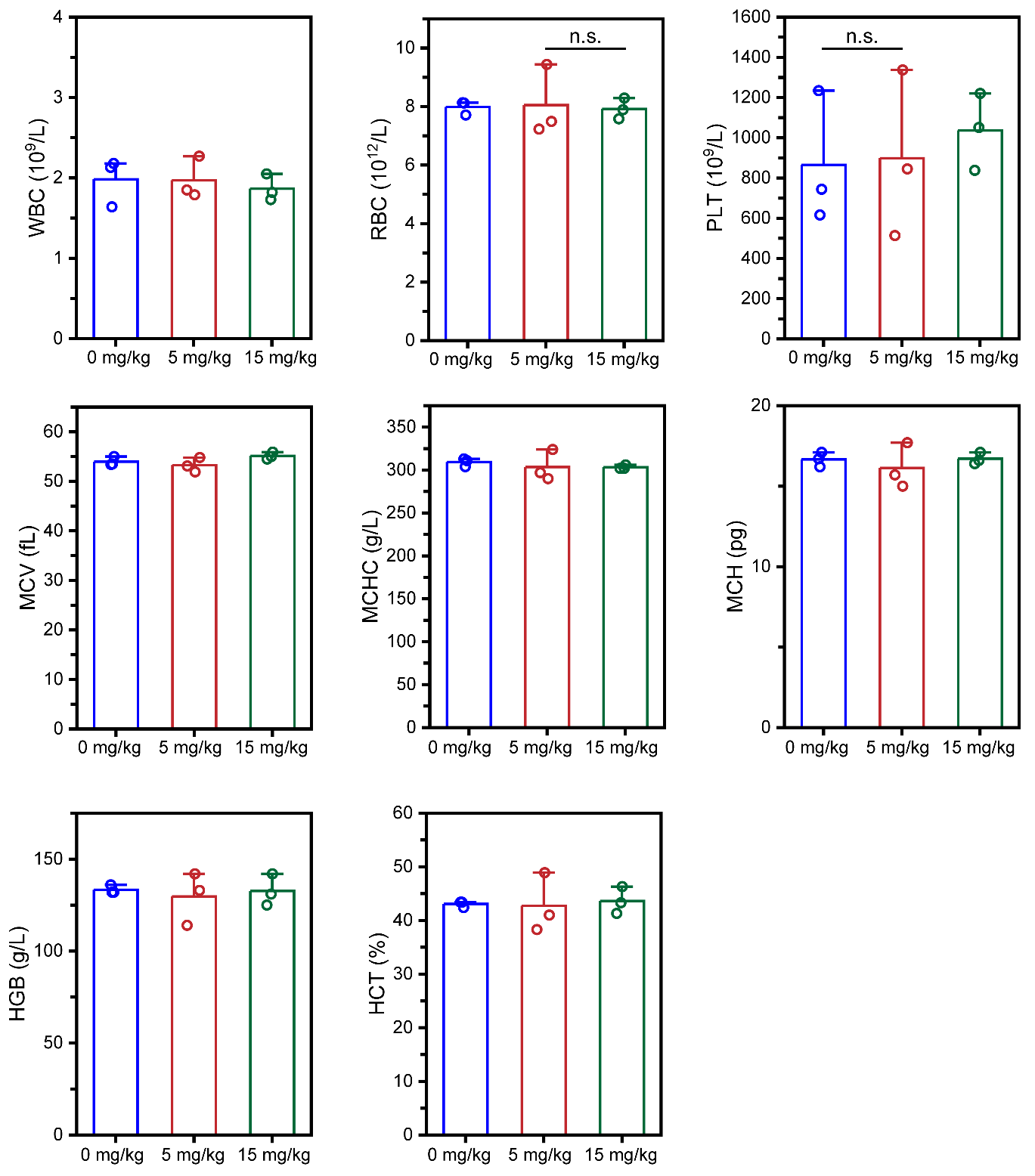


**Fig. S36.** Hematology analysis of healthy mice after treatments with saline (control) or SFN/BQR1@MSPION3 (*C*_Fe_ = 5 or 15 mg/kg).


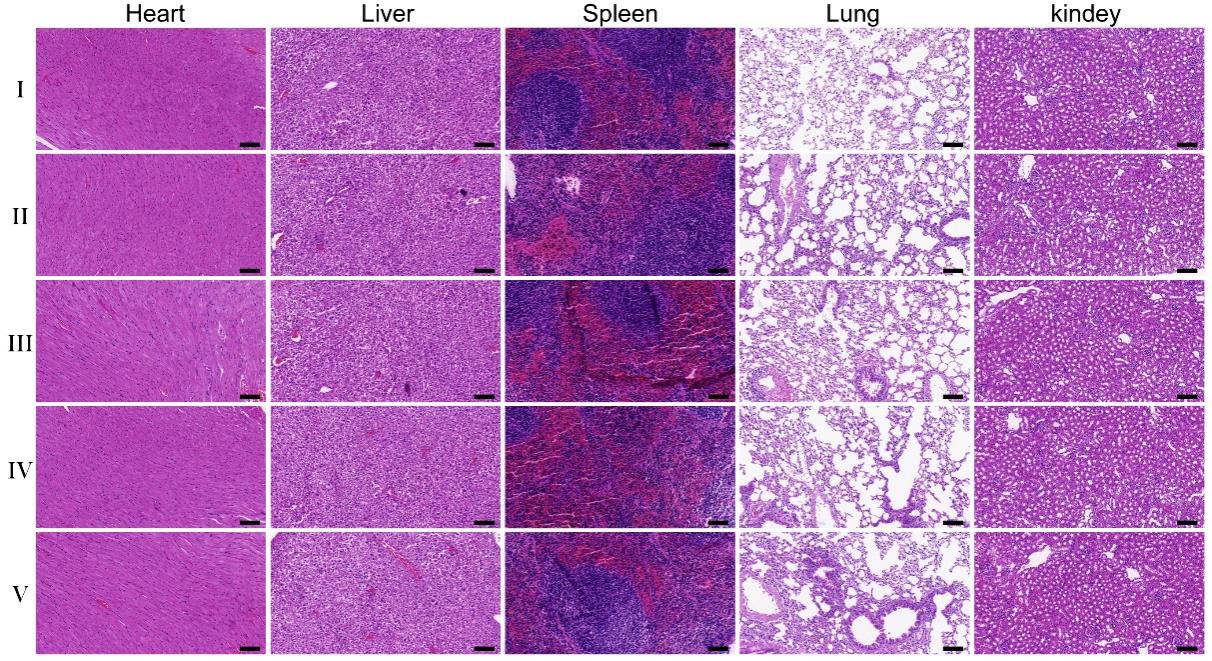


**Fig. S37**. Representative optical microscopic pictures of the H&E-stained main organs (liver, heart, spleen, lung and kidney) from the 4T1 tumor-bearing mice treated with PBS (I), MSPION3 (II), SFN1@MSPION3 (III), BQR1@MSPION3 (IV), or SFN/BQR1@MSPION3 (V) for 14 days.
